# Light-Regulated Agonists Spatiotemporally Activating the Vitamin D Receptor Mitigate Psoriasis-like Inflammation in Mice without Inducing Hypercalcemia

**DOI:** 10.1021/acscentsci.5c00987

**Published:** 2025-10-21

**Authors:** Xavier Rovira, Alfonso Espada, Carme Serra, Juanlo Catena, Marc Lopez-Cano, Silvia Panarello, Elisabet Perez-Albaladejo, Howard Broughton, Leticia Cano, Hans Ajieren, Sadid Khan, Paula Alvarez-Montoya, Lourdes Muñoz, Joan Font, Ana Trapero, Pablo Rivero, Yanrong Li, Donghui Ma, Xianglin Yin, Yanfei L. Ma, Jeffrey A. Dodge, Mingji Dai, Pedro Irazoqui, Francisco Ciruela, Venkatesh Krishnan, Amadeu Llebaria

**Affiliations:** † MCS, Laboratory of Medicinal Chemistry, 203230 Institute for Advanced Chemistry of Catalonia (IQAC), CSIC, Jordi Girona, 18, 08034 Barcelona, Spain; ‡ Centro de Investigación Lilly, SA, Avenida de la Industria 30, 28108 Alcobendas, Spain; § Synthesis of High Added Value Molecules (SIMChem), Institut de Química Avançada de Catalunya (IQAC-CSIC), 08034 Barcelona, Spain; ∥ Pharmacology Unit, Department of Pathology and Experimental Therapeutics, School of Medicine and Health Sciences, Institute of Neurosciences, University of Barcelona, Neuropharmacology & Pain Group, Neuroscience Program, Bellvitge Institute for Biomedical Research, 08907 L’Hospitalet de Llobregat, Spain; ⊥ Weldon School of Biomedical Engineering, 311308 Purdue University, West Lafayette, Indiana 47907, United States; □ Department of Electrical and Computer Engineering, Johns Hopkins University, Baltimore, Maryland 21218, United States; # Department of Chemistry, 311308 Purdue University, West Lafayette, Indiana 47907, United States; ∇ 1539 Eli Lilly and Company, Lilly Corporate Center, Lilly Research Laboratories, Indianapolis, Indiana 46285, United States

## Abstract

Vitamin D receptor (VDR) activation has demonstrated beneficial effects on psoriasis. However, its crucial role in calcium metabolism limits clinical applications due to the risk of health-threatening dysregulation in serum calcium. In the present study, we have designed, synthesized, and biologically tested highly potent light-controllable VDR agonists containing a photoswitchable azobenzene moiety in the drug scaffold. The optimized molecule **PhotoVDRM** is inactive in the dark and can be selectively activated with light using specific wavelengths, including nonphototoxic visible blue light and UVB that is currently used in skin treatments. We used a modified hydrogen/deuterium exchange method to identify the binding site and study VDR dynamics upon ligand binding. Importantly, testing **PhotoVDRM** in a psoriasis mouse model demonstrates that it can spatiotemporally activate VDR in localized diseased areas. Strikingly, this targeted activation results in a robust therapeutic effect without systemic hypercalcemia, thereby addressing at the preclinical level a major historic impediment to VDR agonist treatments in clinical trials. This photopharmacology-based strategy enables the discovery of innovative targeted medicines using light-controlled agonists to spatiotemporally activate the VDR at pathological sites.

## Graphical abstract

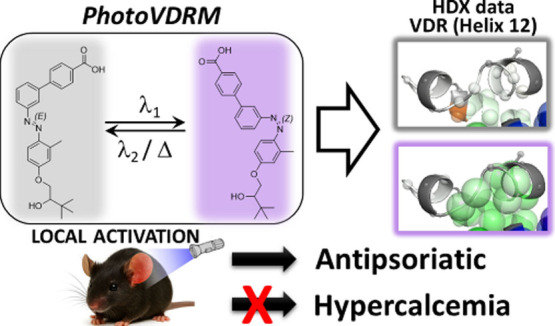

## Introduction

The development of light-regulated drugs represents a promising approach for achieving spatiotemporal precision in targeting therapeutic proteins, minimizing off-target effects, and addressing diseases with unmet clinical needs.^1^ Photopharmacology is one such approach that uses small light-sensitive molecules that can be optimized using traditional drug discovery and development processes.^2^ Therefore, this strategy can be applied to wild type organisms, including humans, without the major concerns arising from other proposed light-based therapies, such as optogenetics.^3^ Among the different photopharmacological strategies, photoswitching drugs can be reversibly photoconverted between biologically active and inactive forms when illuminated with different light wavelengths. This strategy is based on the development of molecules containing a photochromic moiety in their structure, among which azobenzene is commonly used due to its appropriate physicochemical properties and synthetic accessibility. Azobenzene-based photoswitchable drugs have been developed targeting membrane receptors and channels, enzymes, the main components of the cytoskeleton, and nuclear hormone receptors, among others.^4–13^ This targeting technology has demonstrated great potential for the generation of new biological knowledge at both the molecular and physiological level.^14–18^ In addition, therapeutic applications are proposed based on photopharmacology for several diseases with current unmet clinical needs.^19–22^

The implementation of photopharmacological approaches for therapeutic development is hindered by several challenges, with limited light penetration being one of the most significant. Advancements in bioengineering are addressing this challenge through the development of implantable and wearable devices.^23,24^ At the same time, medicinal chemists are developing new strategies to design molecules suitable for photoswitching using light with better tissue penetration.^25–27^ However, with the available technology and knowledge, clinical applications directed to exposed tissues or natural cavities are possible today.^19,28,29^ This is the case of current clinical trials starting phase II in 2025 focused on novel therapeutics for the treatment of orphan retinal diseases (compound kio-301, Kiora Pharmaceuticals).^30^ Probably the most accessible tissue in humans for light-based therapies is the skin, for which a number of diseases still need suitable and economically available treatments. Among skin diseases, psoriasis is one of the most prevalent and debilitating, with 3% of the human population affected and 30% still without appropriate solutions (Global Psoriasis Atlas). New drugs have recently been approved for moderate to severe psoriasis, many of them based on biologicals directed to cytokines producing a significant reduction in inflammation associated with psoriasis.^31,32^ Although these new treatments have demonstrated significant efficacy and minor side effects for certain types of psoriasis, the cost per dose may prevent their general use. In addition, localized psoriasis which falls under the category of mild psoriasis precludes the use of expensive biologicals and subsets of patients suffer from these lesions at sensitive areas such as genital organs.^33^ Therefore, new strategies are being proposed, such as photopharmacological approaches directed to A3 adenosine receptors, which have been found to be overexpressed in the skin and peripheral blood mononuclear cells of psoriasis patients.^19,34,35^ Alternatively, therapies directed to basic mechanisms of the disease may help tackle patients with noncovered types of psoriasis or help improve current therapies.^31^

The vitamin D receptor (VDR) is a member of the nuclear receptor family of transcription factors that plays an important physiological role upon activation by the steroid hormone calcitriol (1α,25-dihydroxyvitamin D3).^36^ These include the regulation of calcium absorption and bone remineralization, endocrine and immune processes, and other functions.^37,38^ Indeed, the alteration of the VDR function and the deficiency of Vitamin D3, the precursor of calcitriol, leads to a variety of serious pathologies such as bone malformations or osteoporosis, and has been suggested to be involved in cancer, diabetes, and autoimmune and cardiovascular diseases.^39,40^ The effects of vitamin D on the immune system in psoriasis are complex. Vitamin D promotes the differentiation of naïve T-cell differentiation into T regulatory cells, thus enhancing the production of anti-inflammatory cytokines (TGF-β, IL-4, and IL-10), and suppressing the production of proinflammatory cytokines (TNFα, INFγ, IL-17A, and IL-2).^41,42^ Calcipotriol, a synthetic vitamin D derivative, decreased the frequency of CD8+ IL-17+ T-cells in psoriatic lesions.^43^ Light is essential to interconvert 7-dehydrocholesterol into vitamin D3 in skin cells, which is subsequently hydroxylated to the active natural derivative calcitriol in the liver and kidney. Upon binding to calcitriol, VDR is translocated from the cytosol to the nucleus where it binds to the retinoid-X receptor (RxR), forms a heterodimer, and interacts with the DNA. Upon recruitment of several cofactors, the whole complex exerts regulatory functions on a large number of genes. Despite recent advances in developing agonist clinical candidates with reduced calcemic effects, the distinction between therapeutic efficacy and calcium-elevating activity remains inadequate to permit oral administration for the treatment of conditions, such as osteoporosis, cancer, leukemia, and psoriasis.

Photopharmacological agents have been demonstrated for several nuclear receptors.^11–13,44–46^ In this study, we present molecules that in *trans* configuration show null activity as VDR agonists for a wide range of concentrations from 1 nM to 10 μM. Significantly, photoisomerization to *cis* with UV and visible light activates the drug producing potent VDR activation *in vitro* and *in vivo*. We elucidate the binding mode by molecular modeling and the dynamics of the receptor using modified hydrogen/deuterium exchange mass spectrometry (HDX-MS) method adapted to photopharmacology. HDX-MS was previously used to monitor the photoisomerization of azobenzene-conjugated peptides but never to study the activation mechanism of the target upon photoactivation.^47^ Remarkably, the therapeutic potential of the molecule is evidenced in animal models of psoriasis, where it effectively resolves disease manifestations without inducing systemic hypercalcemia, which is a critical adverse effect commonly associated with this class of treatments. Overall, in this report, we present evidence of a disease-directed drug development targeting VDR with prospects for clinical application in skin diseases.

## Results and Discussion

### Molecular Design and Synthesis of Vitamin D Receptor Agonists

To design photoswitchable VDR ligands, we aimed at maintaining the essential determinants for the agonist interaction. Regarding conventional ligands, two main VDR ligand structural families can be distinguished, the secosteroidal ligands, related to vitamin D and the active calcitriol hormone, and the diarylmethane nonsecosteroidal family that can be represented by LSN2148936 (Figure 1). Extensive efforts to derive selective agonists from these two main structural scaffolds have been attempted, leading to helpful structural and activity information. To design the library of azobenzene photoswitchable ligands, we analyzed the ligand–receptor contacts of structurally diverse agonists. There are more than one hundred structures in the PDB with the coordinates of VDR-ligand complexes, including some with the natural agonist calcitriol. After grouping and analyzing the most interesting chemotypes, we searched for structure–activity relationship studies of the most interesting candidates. We defined the molecular surface of the VDR bound ligands (Figure 1). The active agonists are characterized by a U-shape rigid structure with a central hydrophobic scaffold bearing two substituents having hydroxyl or other groups that interact with the polar residues in the VDR binding domain. All of this information provided the basis for the development of the first series of compounds containing those groups critical for hydrogen bonding with specific amino acids in the VDR binding pocket, while maintaining the hydrophobicity of the rigid backbone of calcitriol and other agonists. These hypotheses were initially developed by classical medicinal chemistry strategies, evaluated using the structures available in the PDB, and later validated using computational methods for rational drug design (Figure 1). The design process included the introduction of azobenzene as a photochromic moiety, which has the capacity to photoisomerize from the *trans* (E) to the *cis* (Z) states and vice versa when illuminated with light of the appropriate wavelength. The objective was to develop molecules with the ability to interact with the receptor only in the *cis* configuration, resembling the active agonists whereas the *trans* flat structure was more distant from the active ligand geometries. We initially decided to maintain the *t*-butyl phenoxymethyl carbinol side chain present in LSN2148936 and selected the use of a biphenylcarboxylate moiety for the second substituent connected by a N=N central linker. This would provide the candidate molecule with the desired photopharmacological features for future clinical developments, such as negligible activity in the initial dark conditions and activation of the drug upon illumination.

**Figure 1:**
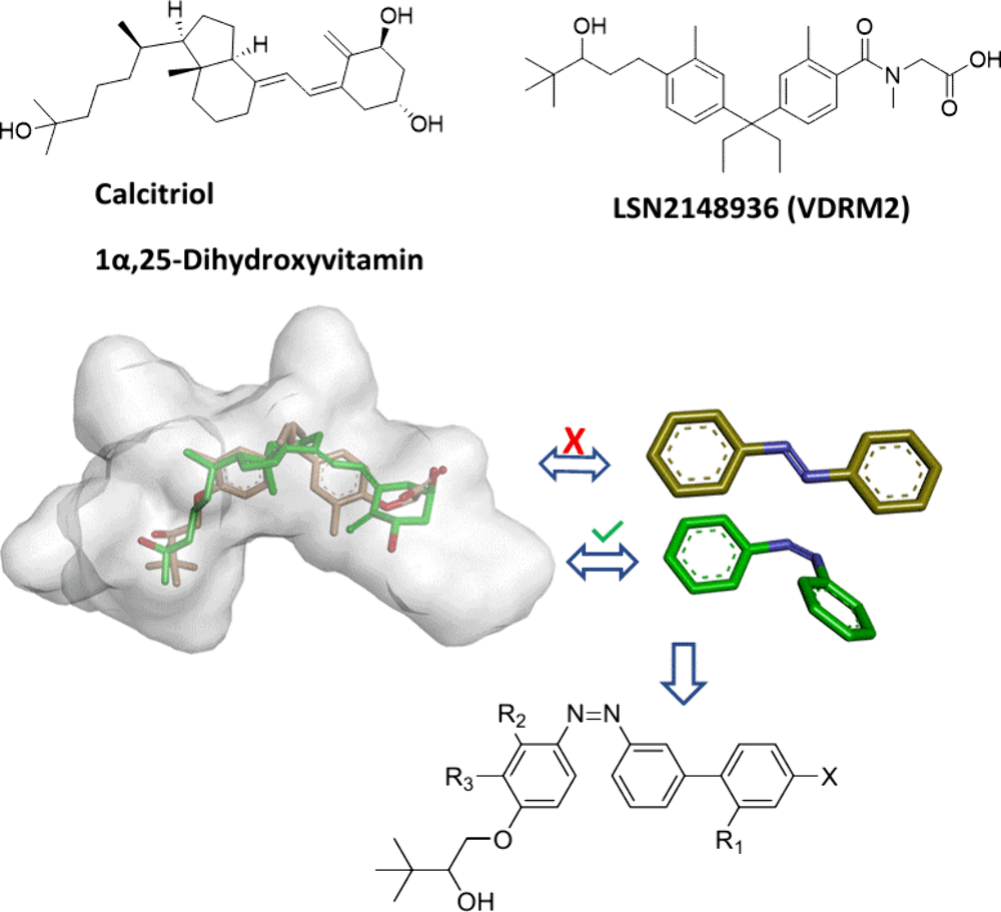
Azobenzene ligand design.

The synthesis of photoswitchable VDR agonists was planned using the Mills reaction of aromatic nitroso compounds with anilines to build up the azobenzene compounds, starting from *p*-bromobenzoic ester **1**, nitrophenyl boronic acid **2**, and *p*-nitrophenols **3a**,**b** (Scheme 1). Accordingly, the Suzuki reaction between boronic acid **2** and bromobenzoate **1** gave nitrobiphenyl ester intermediate **5**, which was reduced by catalytic hydrogenation to aniline **6b**. Key nitroso compounds **7a**,**b** were obtained from **6b** and commercially available aniline **6a** by oxidation with Oxone. On the other hand, racemic aminoalcohols **9a**,**b** were prepared by hydrogen reduction of racemic nitrobenzenes ,**b** (Scheme 1), which were obtained by a nucleophilic epoxide opening reaction between 4-nitrophenols **3a**,**b** and racemic 2-*tert*-butyloxirane **4**. Enantiomeric compounds **10b** and **11b**, with an *S* or *R* specific stereochemistry, were obtained by Mills reaction of nitroso **7b** with homochiral (*S*)- or (*R*)*-* hydroxyanilines **9b** (Scheme 1). The chiral nitroalcohol precursors **8b** were synthesized from phenol **3b** and tosylates (*R*)-**13** or (*S*)-**13**, prepared from (*S*)- or (*R*)*-tert*-leucine as described by Barrett et al.^48^ (Scheme 1 and Supporting Information).

**Scheme 1:**
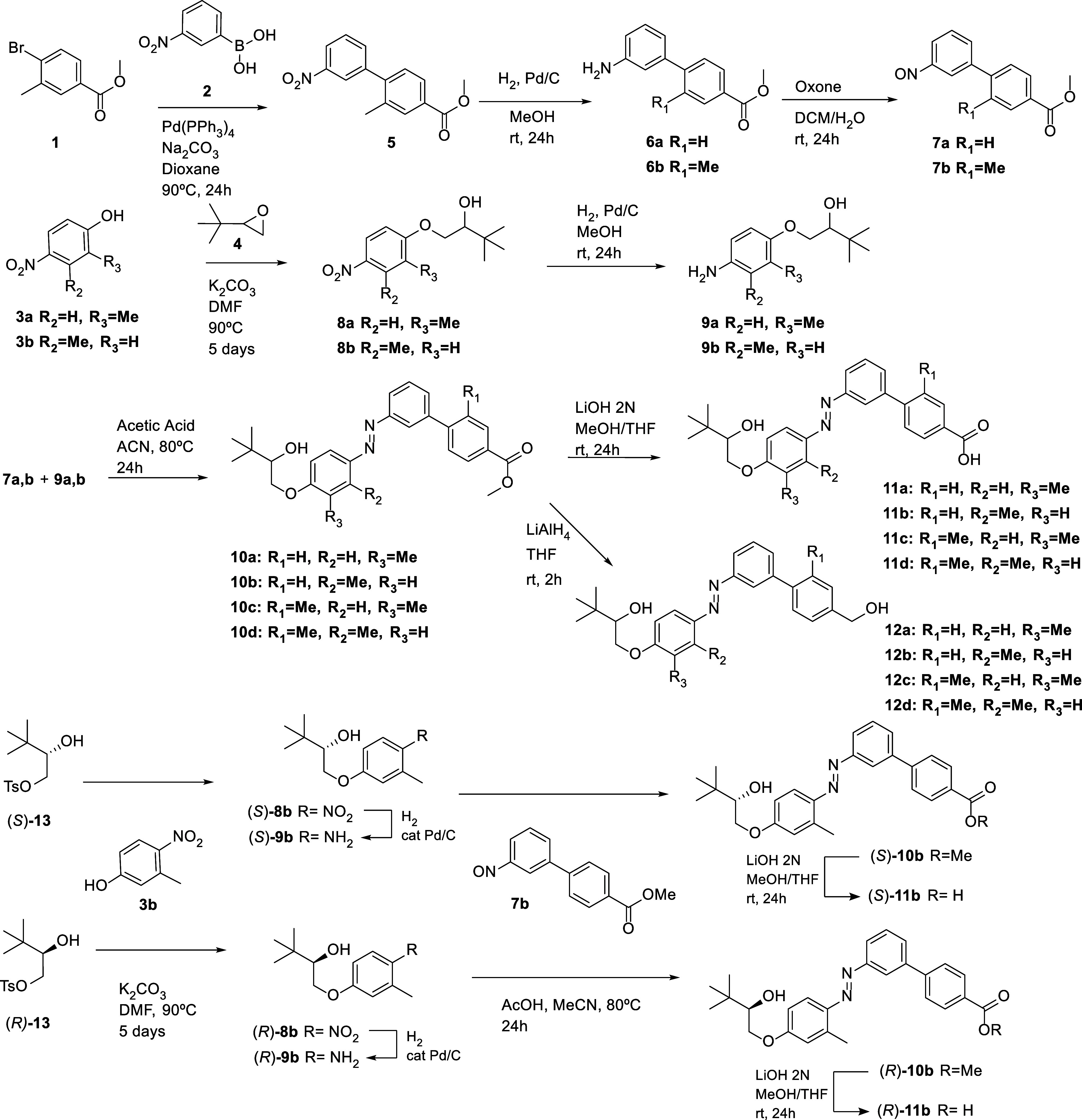
Synthesis of Azobenzene Compounds **10**–**12**

### Photochemistry

The photoisomerization properties of the azobenzene-based molecules developed in this study were evaluated by using spectroscopy methods. *Cis* and *trans* azobenzene isomers are known to have significantly different absorbance spectra in the UV–visible range. The distinct spectroscopic properties between *cis* and *trans* allow for the stabilization of different photoisomeric proportions upon illumination reaching a wavelength-dependent photostationary state (PSS). By introducing modifications in the azobenzene rings, the photochemical properties may be tuned. Therefore, in photopharmacology, light-regulated drugs containing the azobenzene moiety must be characterized to determine the optimal photoisomerization wavelengths and the relaxation times. These photochemical properties will ultimately define the level of control that can be exerted over the biological system.

The UV–vis absorbance spectra of all molecules were measured in the dark and after the application of light of different wavelengths ranging from 365 to 770 nm (Figure 2A and Figures S1–S3). The resulting spectrum in the dark, corresponding to the *trans* configuration of molecules, showed a maximum peak around 363 nm (Table 1). Consequently, violet/UV light below 405 nm efficiently promoted photoisomerization, which produced a dramatic decrease in the π-π* band and a significant increase in the n-π* band at around 445 nm (Table 1). Back-isomerization to the most stable *trans* configuration could be achieved by blue to yellow illumination above 435 nm. The proportion of molecules at the PSS in *trans* isomer upon constant illumination with light at 525 nm and *cis* at 365 nm was in general above 50%, reaching a remarkable >99% for compound **11d** (Table 1 and Figures S4 and S5). Of note, photoisomerization *trans* to *cis* was found to be more efficient in all molecules. The thermal relaxation to the most stable isomer in the dark was measured after illumination of the sample with violet light (Figure 2B and Figures S6–S8). The thermal half-life recorded for these molecules at 37 °C in 100% DMSO is higher than 6 h, demonstrating a long thermal stability in the *cis* state (Table 1). The greater stability of the *trans* isomer was consistent with previous quantum mechanical and molecular mechanics predictions. Measuring the photoisomerization properties of compound **PhotoVDRM** allowed us to determine the photoisomerization quantum yields of the compound by solving a differential equation for photoisomerization.^49^ We used MATLAB to perform regression and arrive at the following photoisomerization quantum yields: ϕ_365 nm (trans)_ = 0.0427, ϕ_365 nm (cis)_ = 0.0501, ϕ_470 nm (trans)_ = 0.1857, and ϕ_470 nm (cis)_ = 0.2281 (Figure S9). Compound **PhotoVDRM** was also evaluated in aqueous buffer used for pharmacological experiments (0.5% DMSO) showing a thermal relaxation of 3 h, almost 7 times faster than in pure DMSO (Figure 2B). To demonstrate reversibility, the absorbance of **PhotoVDRM** was recorded after illumination of the samples with a sequence of 365 and 460 nm lights for six cycles (Figure 2C). The results suggest very efficient interconversion between the two configurations and the absence of photodegradation. A deeper photocharacterization of **PhotoVDRM** allowed us to measure the kinetics of photoconversion upon constant illumination with two wavelengths and three light intensities (Figure 2D and Figure S10). These results demonstrate that the photoisomerization rates for **PhotoVDRM** from *trans* to *cis* states are fast with low intensity light sources. Indeed, with LED devices producing light at intensities as low as 0.05 mW/mm^2^ we were able to transform *trans* to *cis* in around 15.5 s. This time was reduced to 6 and 2 s when the light intensity was increased to 0.15 and 0.75 mW/mm^2^. Overall, the photochemical properties of ligands, in particular the high and fast efficiency of conversion, were found to be suitable for the development of drugs acting in localized areas of an organism. If these molecules demonstrate pharmacological activity only in their *cis* state, we could administer a completely inert agent (100% *trans*) to rapidly and efficiently enrich the area to be treated with an active drug using low intensity light of a wide range of wavelengths. Therefore, we proceeded to test the ability of the chemical library to activate the VDR under different light conditions.

**Table 1:** Photochemical Data of the VDR Agonists

Compound	λ_π–π*_ (*trans*)^a^ (nm)	λ_n−π*_ (*cis*)^a^ (nm)	*t*_1/2_ ^a^ (h)	PSS_365_ ^b^ (% *cis*)	PSS_525_ ^b^ (%*trans*)
**10a**	366	446	9.3	83	86
**10b**	360	440	7.6	78	73
**10c**	366	444	12.5	73	83
**10d**	360	438	8.2	88	81
**11a**	366	440	9.1	94	74
**11b (PhotoVDRM)**	362	442	20.5	95	55
**11c**	364	444	25.4	96	84
**11d**	362	444	17.7	>99	61
**12a**	364	444	9.1	80	87
**12b**	362	444	6.8	84	84
**12c**	364	444	23.2	87	86
**12d**	362	446	8.2	88	82

a Determined at 60 μM in 100% DMSO, 37 °C.

b PSS state areas were determined at 21 °C by HPLC-MS after 3 min illumination (365/525 nm) of a 60 μM sample with a CoolLED pE-4000 in the aqueous buffer used for the pharmacological experiments (0.6% DMSO) without DTT and BSA (Supporting Information). Ratios were determined at the isosbestic point.

**Figure 2:**
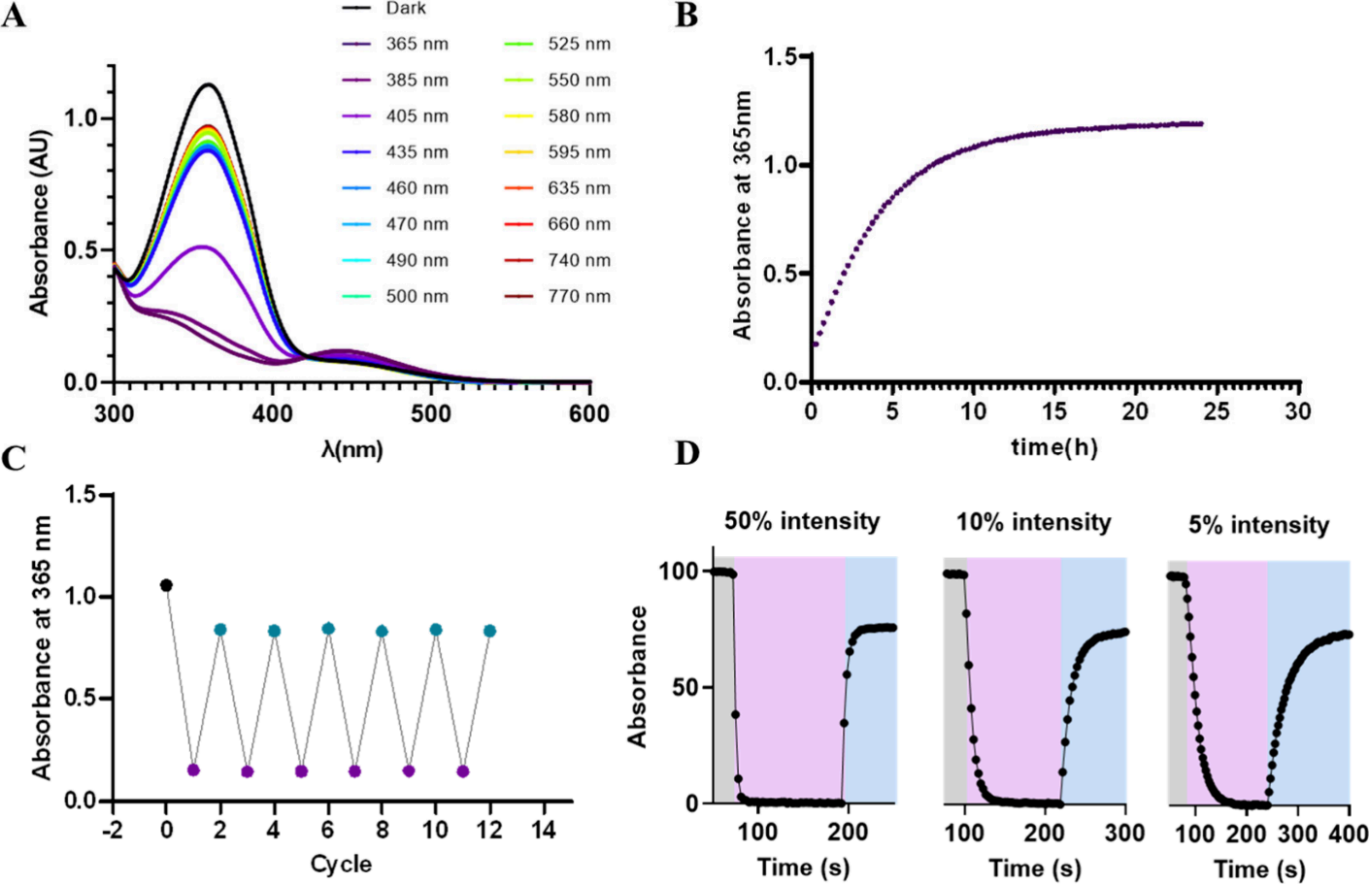
Photochemical characterization of compound **PhotoVDRM**. (A) UV–vis absorption spectra of compound **PhotoVDRM** under different light conditions. (B) Half-lifetime estimation of compound **PhotoVDRM**, absorbance was measured at 365 nm. (C) Multiple *cis*/*trans* isomerization cycles (365/460 nm) show the stability of the compound over 6 cycles of light application. (D) Photoisomerization under constant illumination with light at 365 nm (violet shade) and 470 nm (blue shade). Light was applied with a CoolLED pe-4000 at 50% intensity that corresponded to 0.75 mW/mm^2^ for the light at 365 nm and 0.56 mW/mm^2^ for the light at 470 nm; 10% intensity corresponded to 0.15 mW/mm^2^ for the light at 365 nm and 0.12 mW/mm^2^ for the light at 470 nm; and 5% intensity corresponded to 0.06 mW/mm^2^ for the light at 365 nm and 0.05 mW/mm^2^ for the light at 470 nm. The photoisomerization rates (τ) using light at 365 nm were measured as 2, 6, and 15.5 s with 50%, 10% and 5% of intensity. The measurements were performed at 25 °C in the aqueous buffer used for pharmacological experiments (0.6% DMSO).

### Photopharmacological Activity of the Azobenzene Library

The activity of all molecules was evaluated using a luminescent proximity homogeneous assay to study interactions between purified VDR, RxR and a fragment of the transcriptional coactivator TRAP220 (Supporting Information). This method allowed us to monitor the interaction between TRAP220 and the activated VDR-RxR heterodimer, which is of mechanistic and physiological significance. Both the activation of VDR and the VDR-RxR heterodimer formation are necessary to promote the whole complex association. This system efficiently includes conformational events of the receptor upon activation that go beyond a simple binding measurement.

A first single-dose screening was performed for the rapid detection of the most interesting candidates. This investigation was designed to assess the ability of compounds to bind and activate VDR, with the objective of identifying drugs that are inactive in the *trans* configuration and active in the *cis* configuration (Figure 2A). The chemical library was pharmacologically evaluated at 10 uM in the dark and after illumination with light at 365 nm (Figure 3B). In these experiments, all compounds remained mostly inactive when kept in the dark, whereas significant activity could be measured when illuminated. Since all compounds demonstrated the desired photopharmacological profile with better activity in their *cis* configuration at the concentration assayed, we subsequently performed dose–response experiments in which the potency (EC_50_) and the maximum response (*E*_max_) could be accurately determined (Table 2, Figure 3C, and Figures S11–S13). As expected, all molecules demonstrated activity upon illumination with a 365 nm light. In contrast, the activity in the dark was found to be negligible for a range of concentrations, with the exception of **11a**, which showed a small increase in the activity at 10 μM. Of note, several derivatives demonstrate submicromolar potencies.

**Table 2:**
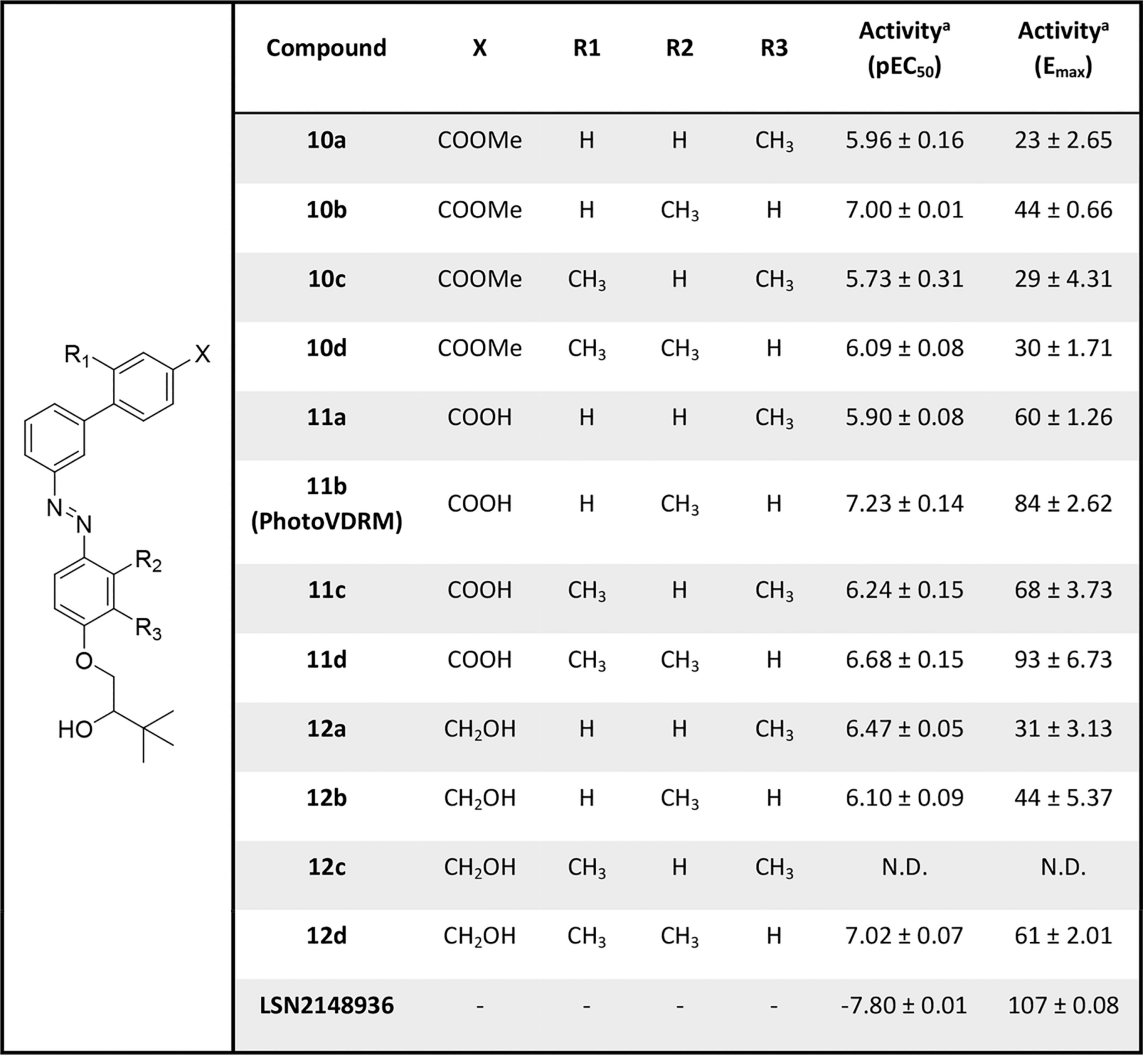
Pharmacological Data of Photoswitchable VDR Agonists

a Activity after illumination (365 nm) for 3 min. All compounds except LSN2148936 we found inactive in the dark with a pEC_50_ < 5.00 (Supporting Information). All data are presented as mean ± SEM of at least *n* = 3 experiments. For compound **12c**, the pEC_50_ and *E*_max_ values could not be determined (N.D.).

**Figure 3:**
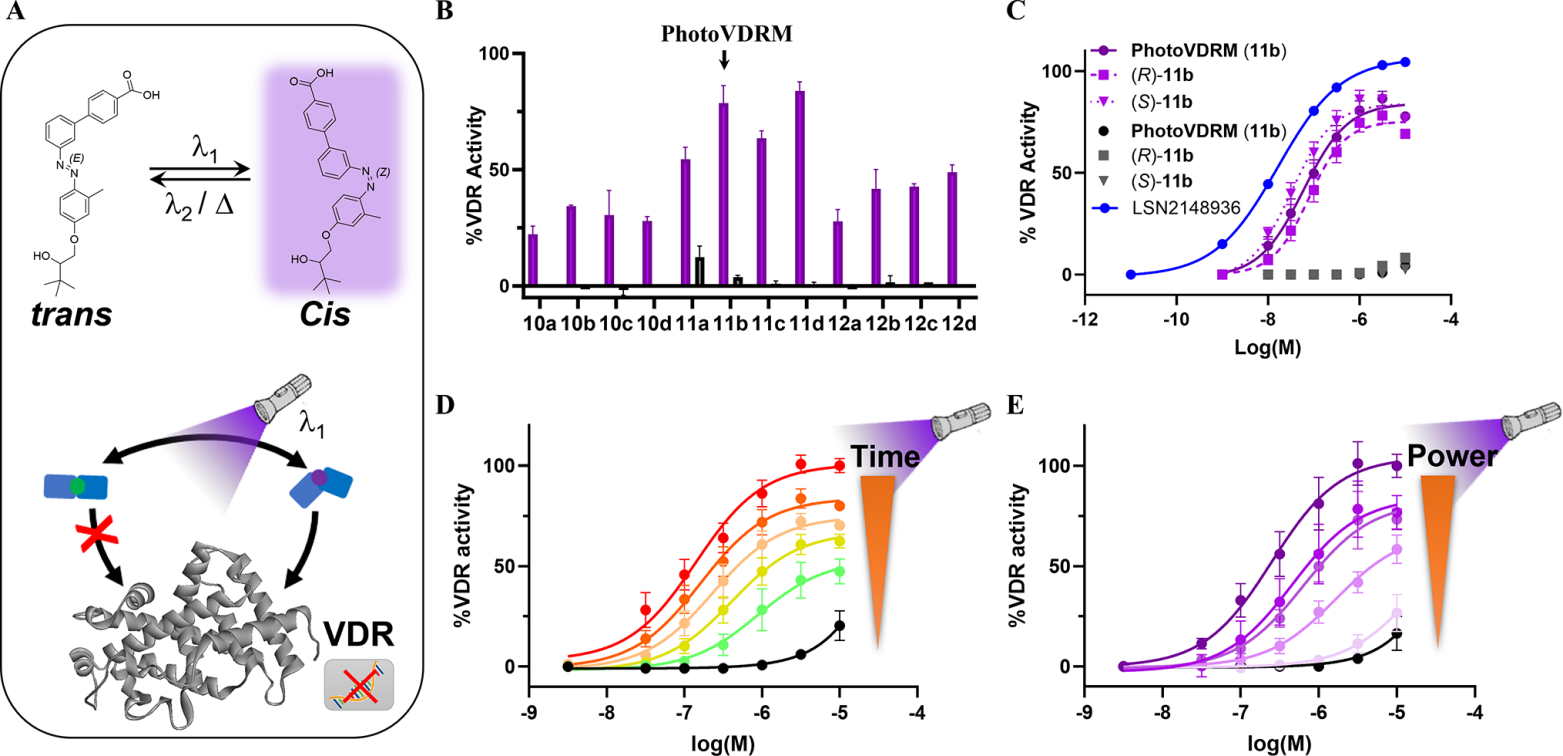
Screening of the compound library and concentration–response curves of racemic and enantiomeric **PhotoVDRM**. (A) Representation of the photopharmacological behavior observed for the chemical library (*cis*-on). (B) Screening of the drug activity at a single dose (10 μM) in the dark (gray bars) and after 365 nm illumination (violet bars) for 3 min. (C) Dose–response curves of compound **PhotoVDRM** racemic and enantiomerically pure in the dark and after illumination (365 nm). (D) Dose–response curves of compound **PhotoVDRM** in dark (black line) and after illumination (365 nm) for 1, 5, 10 20 and 100 s with an intensity of 0.06 mW/mm^2^ (green to red lines, respectively). (E) Dose–response curves of compound **PhotoVDRM** in the dark (black line) and after illumination with increasing light power (2, 5, 10, 20 and 100%) of the CoolLED pe-4000 at 365 nm for 5 s (intensity gradient of violet indicates increasing light power). The experiments were performed at 25 °C in aqueous buffer (Supporting Information). Data are shown as the mean ± SEM of three to four independent experiments in duplicate.

Within the library, the compound **11b**, which we designate as **PhotoVDRM**, was found to be the most potent with a pEC_50_ of 7.23, in the same range of the VDR reference compound LSN2148936 (pEC_50_ = 7.80) and an *E*_max_ greater than 80% (Table 2). It is worth mentioning that control experiments were performed to ensure that the activity of LSN2148936 was not affected by the application of light. In contrast, **PhotoVDRM** artificially decreased the signal of the measurement at high concentrations above 3 μM in the absence of proteins (Figure S14). These results could explain the decrease in the *E*_max_ observed for the**PhotoVDRM** agonist in comparison to that of LSN2148936. Finally, it is well-known that proteins may interact in an enantioselective manner. Since the molecules described here contain a stereogenic center, we obtained individual enantiomers by independent synthesis (Scheme S1) to assign the configuration of **PhotoVDRM**. Upon evaluation of their activity on VDR, it was determined that none of the enantiomers of compound **PhotoVDRM** exhibited remarkable different activities from each other (pEC_50_: 7.10 and 7.48; Table S1) or from the racemic mixture (pEC_50_: 7.22). The maximum response elicited by both pure enantiomers and a racemic mixture was also similar (Figure 3C).

One outstanding advantage of photopharmacology lies in the ability of light-regulated compounds to precisely modulate the activity of proteins through variations in light conditions. To demonstrate this property, we assessed the activity of our hit compound **PhotoVDRM** under various light conditions by modifying both the power and the exposure duration (Figure 3D and 3E). A remarkable control of the activity was observed with illumination times ranging from 1 to 100 s and the modification of light intensities. Of note, we also demonstrated that UVB narrowband light (315 nm), used for phototherapy, and visible illumination at 420 nm modulated the activity of compound **PhotoVDRM** but not that of LSN2148936 (Figure S15). These results highlight the interesting light-regulated pharmacological properties of the chemical library developed and, in particular, those of compound **PhotoVDRM**. To further investigate how this novel VDR drug candidate exerts its precise light-control at the molecular level, we developed experimental methods based on HDX-MS and conducted computational studies to analyze the molecular dynamics of the protein upon binding and activation under different light conditions.

### Molecular Determination of the Light-Controlled Activity of Compound **PhotoVDRM**

We examined the binding mode of the light-regulated **PhotoVDRM** molecule presented in this work using the crystal structure of the human VDR (PDB code: 3B0T). This structure was chosen because it displays a very high resolution (1.30 Å), thus assuring the correct positioning of the amino acid side chains.

A conventional rigid docking protocol was used to introduce the *cis* and *trans* states of **PhotoVDRM** and the control molecule LSN2148936 in the VDR binding pocket (Figure 4). The results show that all molecules and configurations can interact with the receptor. However, only the *cis* isomer accomplishes the well-established interactions of the natural hormone calcitriol, which engages the amino acids W286, L233, R274, H397 and Y401.^50^ In particular, although some of these residues are close enough to establish nonionic molecular interactions with the *trans* state, only the *cis* photoisomer of both enantiomers positions hydrophobic bulk close to the indole ring of W286, with the central ring being planar and at a suitable distance from W286 to form attractive staggered stacking interactions (Figure 4 and Figure S16). The establishment of interactions with residue W286 is a very important molecular determinant for the binding and function of VDR agonists, which is conserved in most of the structures published to date. Moreover, mutations in this position lead to congenital bone malformations and other pathologies,^51,52^ thus highlighting the importance of this residue for the molecular recognition of Calcitriol and the function of the protein. Finally, by the examination of the docking results of both enantiomers, we can conclude that although there are subtle differences between enantiomers, in general terms, very similar interactions with the receptor are satisfied.

**Figure 4:**
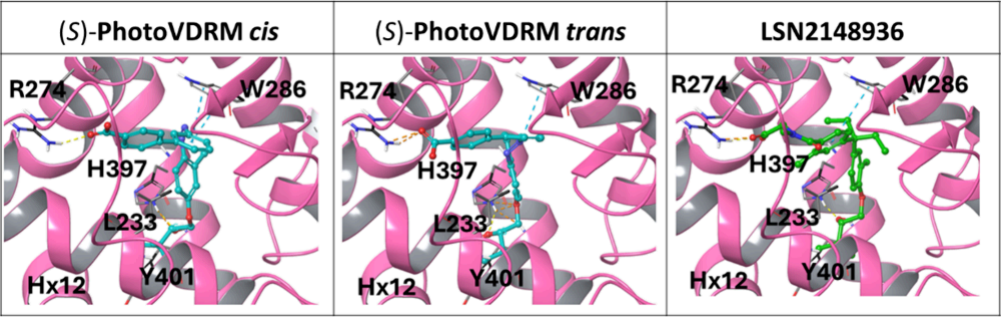
Binding mode of *cis* and *trans* (*S*)-**PhotoVDRM** and reference compound LSN2148936. Key interacting residues are shown as sticks, illustrating how *cis* (*S*)-**PhotoVDRM** and LSN2148936 occupy the space close to residue W286 while *trans* (*S*)-**PhotoVDRM** is more distant.

Subsequent MM/GBSA energy analysis suggested that the *cis* isomer of (*S*)-**PhotoVDRM** would bind more tightly to the VDR than the *trans* one, with both docking scores and MMGBSA binding energies favoring the *cis* isomer (Tables S2–S5). Molecular mechanics and DFT quantum mechanical energy calculations showed that the *cis* isomer was higher in energy by at least 4.5 kcal/mol than the *trans*, suggesting that photoisomerization would promote the ligand from the presumably less active *trans*, to the more active *cis*, form. Visual analysis of the residue-by-residue interaction energies and MMGBSA calculation (Figure 4, Figure S16, and Table S2) show that, as expected,^53^ residues that form characteristic interactions with VD3 ligands such as R274, H305 and H397 are detected as contributing significantly (←5.0 kcal/mol each) to the binding of both forms and to the reference ligand LSN2148936. However, it is notable that particularly H397 and another interesting residue, W286, show weaker interactions with *trans* (S)-**PhotoVDRM** than with the other ligands (). Ligand interaction diagrams (Figure S16) show the residues within 4 Å of the ligand in each case along with the most significant interactions. Thus, all of the molecular modeling studies described here supported our hypothesis that this ligand would bind weakly in *trans* and that the *cis* configuration would be essentially absent in the dark. The difference in EC_50_ and maximum observed effect between the *cis* and *trans* forms of **PhotoVDRM** given the relatively small difference in the docking pose and various energetic scores merit further comment. We believe that the *trans* isomer may bind to the receptor, albeit more weakly than the *cis* isomer, and that it is also less capable of activating the receptor, as can be confirmed in the HDX data (vide infra). The combination of reduced affinity and efficacy likely contributes to the substantial rightward and downward shift of the activity curve. Furthermore, the distinction between the (*S*)- and (*R*)-**PhotoVDRM** isomers is much less pronounced, aligning with the pharmacological data that indicate only minor differences in their activity. Overall, the results of our calculations propose a reasonable binding mode for each enantiomer, which may explain the activity found in the functional assays. Importantly, the lack of hydrophobic interactions observed between the aromatic rings of *trans* **PhotoVDRM** and W286 vs the clear stacking interaction observed for *cis* **PhotoVDRM**, may explain the activity differences found between the dark *trans* state and the light-triggered *cis* state.

### Mapping the Conformational Dynamics of VDR with HDX-MS

Hydrogen/deuterium exchange coupled to mass spectrometry has proved to be a rapid and sensitive approach to interrogate protein-drug interactions and, in particular, it has been shown to be a repeatable and precise method of studying the vitamin D receptor activation dynamics.^54^ Although previously used to study the effect of a cyclic azopeptide in its activated *cis* state,^47^ here we studied the target dynamics upon direct application of the light to the sample. To this aim, HDX-MS was applied for the characterization of the binding mode and modulation of receptor dynamics of the racemic compound **PhotoVDRM**. VDR-LBD was incubated alone (to provide an apo control for each experiment) or with an excess of compound **PhotoVDRM** and exposed to deuterium labeling for various periods of time, from 10 s to 2 h, in the dark and illuminated with light at 365 nm. Strikingly, HDX-MS experiments revealed changes in protection to exchange backbone NH protons (typically observed when ligand interaction modulate the conformational state and dynamic behavior of the protein)^55^ that were significantly stronger in the experiment with light, and also showed some interesting qualitative differences vs the dark state (Figure 5 and Figure S17). As an additional control experiment, we also evaluated the HDX-MS of VDR-LBD in the presence and absence of a standard agonist, compound LSN2148936, in the dark and under illumination conditions. As expected, the HDX behavior of LSN2148936 showed no significant light-dependent changes (Figure S17), and no significant changes were observed in the apo state of the protein, as recorded in any of these experiments (data not shown). In order to permit direct comparison, including mathematical manipulation of the data in all these experiments, the observed deuterium incorporation percentages were converted to rate constants (expressed as −log_10_(k)) using our previously published Bayesian approach.^56^

**Figure 5:**
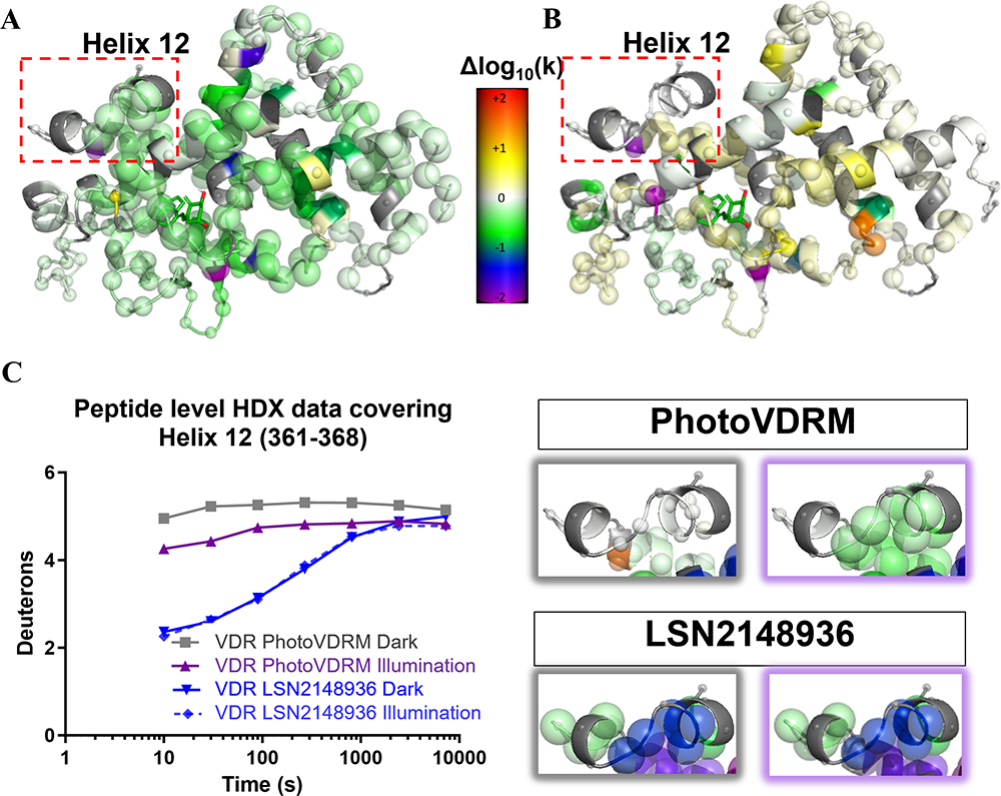
VDR-LBD molecular dynamics was determined by HDX-MS upon ligand binding. Difference between the impact of ligand binding on H/D exchange rates with and without application of light for (A) *cis* **PhotoVDRM** or (B) LSN2148936. The difference between the light and dark experiments after subtracting the equivalent apo experiment is represented for each compound. Note the larger regions with green coloring for **PhotoVDRM** and the lack of any significant difference for LSN2148936. (C) Hydrogen/deuterium exchange protection by ligand and light treatments for the peptide covering Helix 12 (full protein in Figure S17). Note the greater protection afforded by the photoisomerized *cis* **PhotoVDRM** (right panel, violet box) vs the *trans* (right panel, gray box), while no differences are seen for LSN2148936 between illumination conditions. All panels show the VDR molecule modeled on the basis of the ligand present in 3B0T purely as an illustration of the location of the canonical binding pocket. In each panel, the larger the spheres, the higher the confidence that the delta −log_10_(k) value is different from zero.

From the results of these experiments, it is immediately clear that the protection to H/D exchange offered by **PhotoVDRM** in the dark is much lower than that observed under light, where it is comparable with the reference agonist LSN2148936. A number of other significant differences can be observed throughout the ligand binding region, showing that the residues interacting with the protein are different in the dark and under illumination.

A closer examination shows greater protection of Helix 12 in the experiment with the compound **PhotoVDRM** under illumination conditions, lower in magnitude but following a similar trend to LSN2148936 and significantly higher than the protection induced by *trans* **PhotoVDRM** in the absence of light. This suggests that Helix 12 may exhibit reduced structural order and protection due to its proximity to the rest of the protein when the *trans* **PhotoVDRM** is bound, compared to its state in the presence of the *cis* **PhotoVDRM** or the reference compound LSN2148936. This observation is consistent with the hypothesis that *trans* **PhotoVDRM** not only exhibit weaker binding affinity to the protein but may also be less effective in activating the receptor.

### *In Vivo* Light-Dependent Therapeutic Effects of **PhotoVDRM** in a Mouse Model of Psoriasis

Once the activity of **PhotoVDRM** under illumination was validated and the molecular mechanism studied, we aimed to assess its efficacy in a well-established animal model of psoriasis disease.^34^ In this model, we used the visible 420 nm light to induce photoconversion since we demonstrated that this wavelength could produce up to 60% PhotoVDRM *cis* activating VDR (Table S6 and Figure S15) and slightly improve tissue penetrability over shorter wavelengths. Each mice was administered interlueukin 23 (IL-23) once a day for a total of 3 days by intradermal injection (i.d.) into one of the mouse ears. Contralateral ear i.d. injected with PBS was used as control. Subsequently, ear thickness was measured daily, by using a digital caliper, as indicative of psoriasis-course progression (Figure 6A). As previously reported, an i.d. mouse ear injection of IL-23 induced marked swelling in the ear of rodents normalized by its PBS-injected counterpart (Figure 6B, vehicle). Noticeably, ip administration of the LSN2148936 derivative prevented IL-23-associated inflammatory response despite its illumination regime (Figure 6B, LSN). Conversely, under the same time-course experimental conditions, compound **PhotoVDRM** only partially reduces the IL-23-induced psoriatic-like inflammation in its 520 nm photoinduced inactive state (Figure 6B, **PhotoVDRM**). Interestingly, the same racemic **PhotoVDRM** i.p. administered dose showed a significant reduction of the severity of IL-23-induced ear inflammation upon the 420 nm light-irradiation regime. Importantly, those pharmacological effects were observed to begin on the third day (day 4 of the experiment) after its first administration (day 2 of the experiment; Figure 6C and Figure S18). Thus, the *in vivo* 420 nm photoinduced isomerization of **PhotoVDRM** appeared to produce robust anti-inflammatory responses in a physiological environment. Finally, an evaluation of the effects of the treatments on the calcium homeostasis revealed a null impact for compound **PhotoVDRM**, in contrast to the significant effects observed with the reference compound LSN2148936 (Figure 6D). It is important to note that hypercalcemia has been a major side effect for drugs targeting VDR leading to limitations in treatment paradigms for multiple disease states. Overall, these results are indicative that photoconversion of **PhotoVDRM** occurred on a biological tissue upon nontoxic, noninvasive topical irradiation, thus promoting a comparable pharmacological response as reference VDR agonists (i.e., LSN2148936) without signs of hypercalcemia. LSN2148936 is highly selective for VDR and the pharmacological responses are attributed to the activation of VDR and not off-target effects of other steroid receptors, such as the glucocorticoid. Future experiments using enantiomerically pure **photoVDRM** will be conducted to determine whether each isomer exhibits specific effects. Finally, one question that deserves attention is the in vivo decrease in activity upon illumination at 520 nm, despite the remaining *cis* isomer fraction observed in the 520 nm photostationary state during the photochemical characterization in solution­(Table 1 and Figure 2). Multiple factors may underlie this unresolved phenomenon. One possibility is that light may affect the ligand isomerization differently when it is in solution compared to when it is bound to the receptor. Another, potentially concurrent explanation is that the solvent used for the photochemical characterization may not accurately represent the biological environment encountered by the molecule and light in biological tissues. Further studies will be necessary to uncover the mechanisms underlying the activity of **photoVDRM** in vivo, including this and other relevant questions.

**Figure 6:**
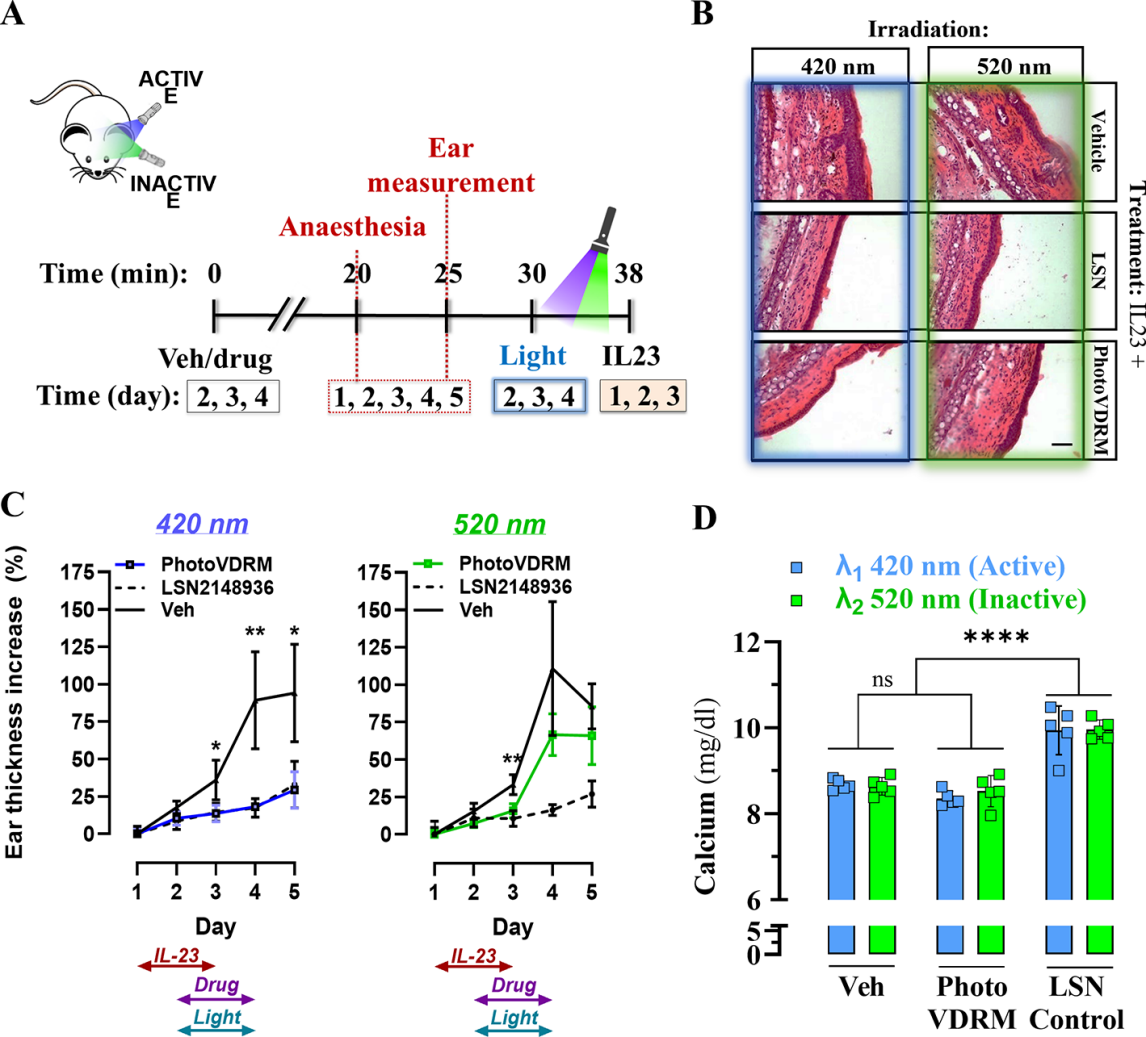
*In vivo* photoinduced antipsoriatic activity of **PhotoVDRM**. (A) Temporal scheme for IL-23-induced mouse model of psoriasis. Animals were treated with IL-23 (i.d.) in the left ear during three consecutive days (days 1, 2 and 3). In addition, a contralateral control was performed consisting of PBS administration in the right ear. At day 2, 3, and 4 animals were previously intraperitoneally (i.p.) administered with vehicle (saline) and drugs (i.e., LSN2148936; 0.005 mg/kg and **PhotoVDRM**; 0.5 mg/kg). Both ears were irradiated with 420 or 520 nm light at day 2, 3, and 4 during 8 min. (B) Representative H&E-stained ear sections of IL-23-treated mice from the indicated experimental group. (C) Inflammatory ear response measurements. Mice ear thickness determination in animals administrated (ip) on days 2, 3, and 4 with vehicle, LSN2148936 or **PhotoVDRM** treated with 420 or 520 nm light. Each day, ear thickness was measured in millimeters and represented as percentage of ear thickness increase using the first measurement of PBS-administrated ear as control. Data is shown as mean ± SD, *n* = 5 mice per group. Statistical analysis between **PhotoVDRM** and vehicle. *P < 0.05 and **P < 0.01 Student’s *t* test. (D) Total calcium measurements. Mice serum was collected after experiments to evaluate increase produced by the treatment. Data are shown as mean ± S.E.M., *n* = 5 mice per group. Statistical analysis between groups treated with 420 vs 520 nm and pharmacological treatments is indicated. ****P < 0.0001 Student’s *t* test.

## Conclusions

We have developed a library of molecules that represent the first photopharmacological toolbox to control VDR activity. The design was based on the introduction of the photochromic azobenzene moiety in a ligand, which allows for photocontrol of the pharmacological activity. Compounds such as **PhotoVDRM,** and analogs, demonstrate activity in the *cis* configuration only, which is obtained when the dark-stable *trans* azobenzene is illuminated. Significantly, this form of light-induced tissue selectivity potentially allows for application to a broader range of diseases mediated by VDR. Remarkably, the most potent molecule **PhotoVDRM** is inactive in the dark for concentrations up to 10 μM and is active with a nanomolar potency after illumination in a functional assay that is mechanistically relevant. This large gain of activity provides a range of concentrations for the complete control of the receptor activity with light. Here, we demonstrate that both **PhotoVDRM** enantiomers exhibit equivalent activity in our *in vitro* assay. Using newly developed HDX-MS methodologies adapted to photopharmacology and computational approaches, we define a binding mode and analyze protein dynamics, offering a plausible explanation for the molecular determinants underlying agonist binding and function. To the best of our knowledge, this is the first report on the application of HDX to evaluate target dynamics in photopharmacology. In a mouse model of skin inflammation, we demonstrate that only the light-activated form of **PhotoVDRM** produces a robust anti-inflammatory response. Indeed, the molecule shows full antipsoriatic activity upon illumination with nonphototoxic visible blue light and no activity with green light. Significantly, the application of a localized and temporally restricted treatment using **PhotoVDRM** and light *in vivo* occurs without concomitant hypercalcemia. This is the first demonstration of the systemic administration of the inactive form of a drug targeting VDR that can be precisely and spatiotemporally activated in the skin region to be treated by photopharmacology, thus avoiding detrimental calcemic effects. As such, this approach may offer a noninvasive regio-specific intervention in psoriasis patients that carry skin lesions, including those in sensitive areas, such as the genitalia. Overall, this strategy has the potential to regulate physiology with high spatiotemporal precision and can be applied for the development of future therapies targeting VDR and other nuclear receptors.

## Supplementary Material





## Notes

Additional experimental details, materials, and methods, including synthetic routes, general methods and analytical results; photochemical and pharmacological characterization plots; quantum yield calculations; HDX-MS analysis; molecular modeling; and animal experiments (PDF)
Transparent Peer Review report available (PDF)

## ○

Enantia, S. L. U., Carrer Baldiri Reixac, 10, 08028 Barcelona, Spain

## ●

Department of Chemistry, Emory University, Atlanta, Georgia 30322, USA.

## △

Evozyne Inc., Chicago, Illinois 60614, USA.
