# Supplementary material for: Light-Regulated Agonists Spatiotemporally Activating the Vitamin D Receptor Mitigate Psoriasis-like Inflammation in Mice without Inducing Hypercalcemia
Source: ACS Cent Sci. 2025 Oct 21;11(12):2340–52. doi: 10.1021/acscentsci.5c00987 (PMC12746157; doi:10.1021/acscentsci.5c00987)
Supplement: Supplementary file 1 [file oc5c00987_si_001.pdf]

Supporting Information

Light-regulated agonists spatiotemporally activating the Vitamin D receptor mitigate psoriasis-like inflammation in mice without inducing hypercalcemia

Xavier Rovira [1] *, Alfonso Espada [2]; Carme Serra [1,3], Juanlo Catena [1,3]; Marc Lopez-Cano [4], Silvia Panarello [1,#]; Elisabet Perez-Albaladejo [1], Howard Broughton [2]; Leticia Cano [2], Hans Ajieren [5], Sadid Khan [5], Paula Alvarez-Montoya [4]; Lourdes Muñoz [1,3], Joan Font [1], Ana Trapero [1], Pablo Rivero [1], Yanrong Li [6,$]; Donghui Ma [6,$], Xianglin Yin [6,$], Yanfei L. Ma [7], Jeffrey A. Dodge [7], Mingji Dai [6,$], Pedro Irazoqui [5], Francisco Ciruela [4], Venkatesh Krishnan [7,&*], Amadeu Llebaria [1*].

[1] MCS, Laboratory of Medicinal Chemistry, Institute for Advanced Chemistry of Catalonia (IQAC), CSIC, Jordi Girona, 18, 08034, Barcelona, Spain.

[2] Centro de Investigación Lilly, SA, Avenida de la Industria 30, 28108, Alcobendas, Spain.

[3] Synthesis of High Added Value Molecules (SIMChem), Institut de Química Avançada de Catalunya (IQAC-CSIC), 08034, Barcelona, Spain

[4] Pharmacology Unit, Department of Pathology and Experimental Therapeutics, School of Medicine and Health Sciences; Institute of Neurosciences, University of Barcelona; Neuropharmacology & Pain Group, Neuroscience Program, Bellvitge Institute for Biomedical Research, 08907 L'Hospitalet de Llobregat, Spain.

[5] Weldon School of Biomedical Engineering, Purdue University, West Lafayette, Indiana 47907, USA; Department of Electrical and Computer Engineering, Johns Hopkins University, 21218 Baltimore, MD, USA.

[6] Department of Chemistry, Purdue University, West Lafayette, Indiana 47907, USA.

[7] Eli Lilly and Company, Lilly Corporate Center, Lilly Research Laboratories, Indianapolis, IN, 46285, USA

* Corresponding authors.

Present addresses:

[#] Enantia, S. L. U., Carrer Baldiri Reixac, 10, 08028, Barcelona, Spain

[$] Department of Chemistry, Emory University, Atlanta, Georgia 30322, USA.

[&] Evozyne Inc., Chicago, IL 60614.

TABLE OF CONTENTS

Experimental Procedures

1. Synthetic General Methods
2. Photochemical characterization
3. Pharmacological characterization
4. Hydrogen/deuterium exchange coupled to mass spectrometry (HDX-MS)
5. Photoisomerization Quantum Yield
6. Molecular Modeling and MM/GBSA and quantum mechanical calculation
7. IL23-induced psoriatic-like animal model

Supplementary Tables

Table S1. Pharmacological activity of **11b** isòmers
Table S2. MMGBSA residue-based interaction energies (kcal/mol) for key residues in the best MMGBSA pose found for various ligands
Table S3. Relative energies of *cis* and *trans* **11b** using various models
Table S4. GLIDE docking conditions applied.
Table S5. GLIDE Docking scores and MMGBSA dG Bind (binding energy, OPLS4) values for the best poses found according to each score
Table S6. Proportinons of cis and trans at the photostationary state in DMSO

Supplementary Figures

Scheme S1 Synthesis of enantiomeric nitroalcohols 8
Figure S1. Photochemical properties of ester derivatives
Figure S2. Photochemical properties of carboxylic acid derivatives
Figure S3. Photochemical properties of alcohol derivatives
Figure S4. Photostationary state areas of **11b** determined by HLPC in the dark and after illumination
Figure S5. Photostationary state areas of the compound series determined by HLPC in the dark and after illumination at 365 nm and 525nm
Figure S6. Thermal relaxation of ester derivatives
Figure S7. Thermal relaxation of carboxylic acid derivatives
Figure S8. Thermal relaxation of alcohol derivatives
Figure S9. Photoisomerization Quantum Yield calculation.
Figure S10. Photophysical properties of **11b** under constant illumination.
Figure S11. Concentration-response curves of ester derivatives
Figure S12. Concentration-response curves of carboxylic acid derivatives
Figure S13. Concentration-response curves of alcohol derivatives
Figure S14. Pharmacological controls. Light does not affect LSN and **11b** does not act as antagonist

Figure S15. Pharmacological activity of **11b** and LSN2148936 upon irradiation with different light wavelengths
Figure S16. MMGBSA optimal binding modes of various ligands to a model of VDR derived from 3B0T.pdb
Figure S17. Differential deuterium uptake of VDR-LBD in the presence of ligand and different light conditions
Figure S18. Controls of mouse psoriasis model.

Experimental Procedures

1. Synthetic General Methods. All the Chemicals and solvents are from commercial suppliers and used without purification, except the anhydrous solvents such as DMF which were treated previously through a system of solvent purification (PureSolv), degasified with inert gases and dried over alumina or molecular sieves. Reactions were monitored by thin layer chromatography (60 F, 0.2 mm, Macherey-Nagel) by visualisation under 254 and/or 365 nm lamp. Purification was made by Flash column chromatography by using Merck Silica Gel 60, 40-63 microns RE or by Phase Reverse with an Isolera-Biotage equipment (SNAP KP-C18-HS; A: Water/Formic acid (0.05%), B: ACN/Formic Acid (0.05%): 5%B 3CV, 5%B-100%B 18CV, 100%B 5CV). NMR were performed in a Brucker 400 MHz. Chemical shifts δ are reported in parts per million (ppm). Azo compounds existed in two isomeric forms, but in NMR spectra, only was assigned to the *trans* configuration based on abundance. HPLC-MS were obtained in a HPLC 2795 Alliance Waters Aquity coupled to Detector DAD Agilent 1100 and Detector MS Waters ESI triple quadrupole Quattro micro, after injection of 10 μL of sample in ACN, using a ZORBAX Extend-C18 3.5 μm 2.1x50mm (Agilent) column. The mobile phase used was a mixture of A = water + 0.05 formic acid and B = ACN + 0.05 formic acid with method described as follows: flow 0.5 mL/min; 5% B for 0.5 min; 5% to 100% B in 5 min, 100% B for 2 min. High Resolution Mass Spectroscopy (HRMS) were analysed by FIA with Ultra-High-Performance Liquid Chromatography (UPLC) Acquity (Waters) coupled to LCT Premier Orthogonal Accelerated Time of Flight Mass Spectrometer (TOF-MS) (Waters). Data from mass spectra was analyzed by electrospray ionization in positive mode using MassLynx software version 4.1 (Waters), which provides experimental and calculated mass as neutral species. Spectra have been scanned between 50 and 1500 Da with values every 0.2 seconds and peaks are reported as m/z (% of basis peak). The enantioselective analyses and purifications were conducted using standard normal mobile phase comprised of n-hexane in combination with IPA and formic acid or trifluoroacetic acid as additives. UV analyses were performed at fixed wavelength (360 nm) for all compounds. The chiral analytical HPLC system consisted of a Dionex LPG-3400SD, equipped with a Dionex ACC-3000 autosampler, a Dionex VWD-3400-RS UV detector and collection data was performed using Chromeleon software from Thermofisher. Enantiomeric purifications were conducted on a waters 1525P semi-prep HPLC coupled to a waters 2489 UV detector, a waters fraction collector III and collection data was performed using Empower software from Waters. Three general methods were used for separation of enantiomer from racemic samples and

enantiomeric excess analysis of chiral compounds: Method Chiral 1. Enantiomeric separation of racemic sample was accomplished using a Chiralpak IB column (10x250mm, 5µm) (Daicel), and an isocratic mixture of hexane and IPA at 97:3, containing a 0.1% of formic acid. Flow was set at 5ml/min for 30 min and fractions containing pure enantiomer were evaporated together to afford each enantiomer separately. Enantiomeric excess was determined using a Chiralpak IB column (4.6x250mm, 5µm) (Daicel), and an isocratic mixture of hexane and IPA at 97:3, containing a 0.1% of formic acid at 1ml/min; Method Chiral 2. Enantiomeric excess was determined using a Lux Amylose-2 column (4,6x250mm, 5µm) (Phenomenex) and a mixture of hexane-EtOH in an 85:15 ratio at a flow rate of 1ml/min; Method Chiral 3. Enantiomeric excess was determined using a Lux Amylose-2 column (4,6x250mm, 5µm) (Phenomenex) and a mixture of hexane-EtOH in an 85:15 ratio containing a 0.1% of trifluoroacetic acid at a flow rate of 1ml/min. Optical rotations were measured on a Perkin Elmer 341 polarimeter using a Na/Hal lamp set at 589 nm.

Methyl 2-methyl-3'-nitro-[1,1'-biphenyl]-4-carboxylate (5)

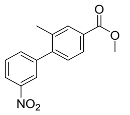

A suspension of 1.31g of methyl 4-bromo-3-methylbenzoate **(1)**, 1g of (3-nitrophenyl)boronic acid **(2)**, Na_2CO_3 (4 eq) and $Pd(Ph_3)_4$ (0.05 eq) in dioxane (0.5 M) was refluxed overnight. Then was concentrated under reduced pressure. The residue was purified by silica gel chromatography eluting with hexane: EtOAc (10:1) and 533 mg of **(5)** were obtained (34% yield) as a white solid. ^{1}H-NMR (400 MHz, $CDCl_3$) δ 8.23 (dt, *J* = 7.6, 1.5 Hz, 1H), 8.18 (t, *J* = 1.5 Hz, 1H), 7.97 (s,1H), 7.91 (d, *J* = 8 Hz,1H), 7.65 (dt, *J* = 7.6, 1.5 Hz, 1H), 7.60 (t, *J* = 7.6 Hz, 1H), 7.28 (d, *J* = 8 Hz, 1H), 3.93 (s, 3H), 2.30 (s, 3H). ^{13}C-NMR (101 MHz, $CDCl_3$) δ 166.9, 148.3, 143.9, 142.6, 135.7, 135.1, 131.9, 130.1, 129.9, 129.4, 127.4, 124.0, 122.5, 52.4, 20.4. HRMS (ESI+): m/z calcd for $C_{15}H_{14}NO_4^+$ $[M + H]^+$ = 272.0923; found 272.0903.

General Procedure for Ether formation (Method A): Synthesis of intermediate type (8): To a stirred solution of intermediates **(3)** (1 eq) in DMF (0.1 M), K_2CO_3, (2 eq) was added, in small portions or dropwise, and the reaction mixture was stirred at r.t. for 10 min. Then, the compound **(4)** was added (5 eq), and the mixture was warmed at 90°C for 5 days. Then, the mixture was treated with EtOAc/water, the organic layer was dried over anhydrous $MgSO_4$, filtered, and

concentrated in vacuum. The residue was purified by flash chromatography (silica gel) to afford compounds type **(8).**

(+/-)-3,3-Dimethyl-1-(2-methyl-4-nitrophenoxy)butan-2-ol ((+/-)-8a)

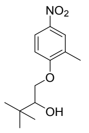

According to method A, from 450 mg of 2-methyl-4-nitrophenol **(3a)**, 290 μL of 2-(*tert*-butyl)oxirane **(4)** and 370 mg of K_2CO_3 in 10 mL of DMF, 360 mg of **((+/-)-8a)** were obtained (48% yield) once it was purified by using (hexane-EtOAc, 3:1) as solvent. ^{1}H NMR (400 MHz, $CDCl_3$) δ 8.06 (d, *J* = 9 Hz, 1H), 8.01 (s, 1H), 6.85 (d, *J* = 9.0 Hz, 1H), 4.20 (dd, *J* = 9.2, 2.6 Hz, 1H), 3.99 (t, *J* = 9.2 Hz, 1H), 3.75 (dd, *J* = 9.2, 2.6 Hz, 1H), 2.27 (s, 3H), 1.02 (s, 9H). ^{13}C-NMR (101 MHz, $CDCl_3$) δ 162.03, 141.27, 127.95, 126.17, 123.66, 110.34, 77.29, 70.47, 33.88, 26.10, 16.51. HRMS (ESI+): m/z calcd for $C_{13}H_{20}NO_4^+$ $[M + H]^+$ = 254.1378; found 254.1392.

(+/-)-3,3-Dimethyl-1-(3-methyl-4-nitrophenoxy)butan-2-ol ((+/-)-8b)

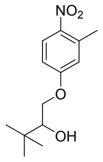

According to method A, from 560 mg of 3-methyl-4-nitrophenol **(3b)**, 490 □L of 2-(*tert*-butyl)oxirane **(4)** and 371 mg of K_2CO_3, 12 mL of DMF, 770 mg of **((+/-)-8b)** were obtained (39% yield) once it was purified by using (Hexane-EtOAc, 7:3) as solvent.^{1}H-NMR (400 MHz, $CDCl_3$) δ 8.06 (d, *J* = 8.7 Hz, 1H), 6.79 (dd, *J* = 8.7, 2.8 Hz, 1H), 6.78 (d, *J* = 2.8 Hz, 1H), 4.15 (dd, *J* = 9, 2.4 Hz, 1H), 3.92 (t, *J* = 9 Hz, 1H), 3.68 (dt, *J* = 9, 2.4 Hz, 1H), 2.61 (s, 3H), 1.00 (s, 9H). ^{13}C-NMR (101 MHz, $CDCl_3$) δ 162.20, 142.41, 137.12, 127.57, 118.02, 112.41, 77.16, 62.67, 33.74, 26.02, 21.66.

(-)-(*R*)-3,3-Dimethyl-1-(3-methyl-4-nitrophenoxy)butan-2-ol ((-)-8b)

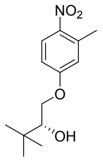

According to method A, from 2.89 g of 3-methyl-4-nitrophenol **(3b)**, 6.17g of (*R*)-2-hydroxy-3,3-dimethylbutyl 4-methylbenzenesulfonate (prepared as is described in US2005/0043368 for the S-enantiomer) and 5.22 g of K_2CO_3, 63 mL of DMF, 172 mg of **((-)-8b)** were obtained (4% yield) once it was purified by using (Hexane-EtOAc, 7:3) as solvent. [α] D_{20} = -20 (c 1.1, $CHCl_3$)

(+)-(*S*)-3,3-Dimethyl-1-(3-methyl-4-nitrophenoxy)butan-2-ol **((+)-8b)**

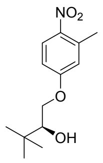

According to method A, from 2.04 g of 3-methyl-4-nitrophenol **(3b)**, 5.43 g of (*S*)-2-hydroxy-3,3-dimethylbutyl 4-methylbenzenesulfonate (prepared as is described in US2005/0043368) and 3.67 g of K_2CO_3, 44 mL of DMF, 550 mg of **((+)-8b)** were obtained (16% yield) once it was purified by using (Hexane-EtOAc, 7:3) as solvent. [α] D_{20} = +28 (c 0.3, $CHCl_3$)

General Procedure for Nitro Reduction (Method B): Synthesis of intermediates (6) and (9): To a solution of nitro derivatives **(5)** or **(8)** (1 eq) in MeOH (0.1 M) or MeOH/THF (1:1) (0.05 to 0.015 M) was added Pd-C (10% W/W) and the mixture was stirred for 24 h at r.t. under a hydrogen atmosphere. The reaction mixture was then filtered by using celite, and the filtrate was evaporated under vacuum yielding the amines **(6)** or **(9)** respectively, and they were used without further purification.

Methyl 3'-amino-2-methyl-[1,1'-biphenyl]-4-carboxylate **(6b)**

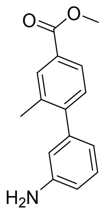

According to method B, from 250 mg of compound **(5)** in MeOH, 220 mg of **(6b)** (99% yield) were obtained.^{1}H-NMR (400 MHz, $CDCl_3$) δ 7.91 (s, 1H), 7.85 (dd, *J* = 8.0, 2 Hz, 1H), 7.26 (d, *J* = 8.0 Hz, 1H), 7.19 (t, *J* = 8.0 Hz, 1H), 6.68 (dd, *J* = 8.0, 2.0 Hz, 2H), 6.60 (t, *J* = 2 Hz, 1H), 3.91 (s, 3H), 3.71 (brs, 2H), 2.30 (s, 3H). ^{13}C-NMR (101 MHz, $CDCl_3$) δ 167.37, 146.98, 146.35, 142.28, 135.81, 131.52, 129.85, 129.27, 128.91, 126.99, 119.46, 115.69, 114.22, 52.21, 20.56.

(+/-)-1-(4-Amino-3-methylphenoxy)-3,3-dimethylbutan-1-ol ((+/-)-9b)

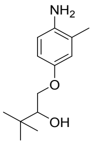

According to method B, from 770 mg of compound ((+/-)-8b) in MeOH (0.3 M), 635 mg of ((+/-)-9b) (94% yield) were obtained.^{1}H NMR (400 MHz, $CDCl_3$) δ 6.88 (d, *J* = 8.6 Hz, 1H), 6.71 (d, *J* = 2.9 Hz, 1H), 6.66 (dd, *J* = 8.6, 2.9 Hz, 1H), 4.03 (dd, *J* = 9.1, 2.5 Hz, 1H), 3.78 (t, *J* = 9.1 Hz, 1H), 3.63 (dd, *J* = 9.1, 2.5 Hz, 1H), 2.26 (s, 3H), 0.98 (s, 9H).

(-)-(*R*)-1-(4-Amino-3-methylphenoxy)-3,3-dimethylbutan-2-ol ((-)-9b)

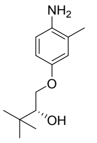

According to method B, from 172 mg of compound ((-)-8b) in MeOH (0.05 M), 150 mg of ((-)-9b) (99% yield) were obtained.

^{1}H NMR (400 MHz, CD_3OD) δ 6.69 (d, *J* = 8.6 Hz, 1H), 6.67 (*d*, *J* = 2.8 Hz, 1H), 6.62 (dd, *J* = 8.6, 2.8 Hz, 1H), 4.03 (dd, *J* = 9.9, 2.7 Hz, 1H), 3.77 (dd, *J* = 9.9, 8.2 Hz, 1H), 3.55 (dd, *J* = 8.2, 2.7 Hz, 1H), 2.15 (s, 3H), 0.97 (s, 9H). [α] D_{20} = -32 (c 0.07, MeOH).

(+)-(*S*)-1-(4-Amino-3-methylphenoxy)-3,3-dimethylbutan-2-ol ((+)-9b)

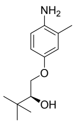

According to method B, from 550 mg of compound ((+)-8b) in MeOH (0.05 M), 450 mg of ((+)-9b) (93% yield) were obtained.

^{1}H NMR (400 MHz, CD_3OD) δ 6.69 (d, *J* = 8.6 Hz, 1H), 6.67 (d, J = 2.8 Hz, 1H), 6.62 (dd, J = 8.6, 2.8 Hz, 1H), 4.04 (dd, *J* = 9.9, 2.7 Hz, 1H), 3.77 (dd, *J* = 9.9, 8.2 Hz, 1H), 3.54 (dd, *J* = 8.2, 2.7 Hz, 1H), 2.16 (s, 3H), 0.98 (s, 9H). [α] D_{20} = +37 (c 0.2, MeOH)

(+/-)-1-(4-Amino-2-methylphenoxy)-3,3-dimethylbutan-2-ol ((+/-)-9a)

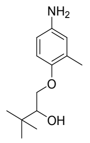

According to method B, from 360 mg of compound (**(+/-)-8a**) in MeOH (0.05 M), 317 mg of (**(+/-)-9a)** (100% yield) were obtained.

^{1}H NMR (400 MHz, $CDCl_3$) δ 6.67 (d, *J* = 8.5 Hz, 1H), 6.57 (d, *J* = 2.8 Hz, 1H), 6.52 (dd, *J* = 8.5, 2.8 Hz, 1H), 4.02 (dd, *J* = 9.0, 2.6 Hz, 1H), 3.79 (t, *J* = 9.0 Hz, 1H), 3.68 (dd, *J* = 9.0, 2.6 Hz, 1H), 2.18 (s, 3H), 1.00 (s, 9H).^{13}C NMR (101 MHz, $CDCl_3$) δ 150.1, 140.0, 128.0, 118.61, 113.5, 113.3, 77.4, 70.5, 33.6, 26.1, 16.4. HRMS (ESI+): m/z calcd for $C_{13}H_{22}NO_2^+$ $[M + H]^+ = 224.1643$; found 224.1651.

General Procedure for Nitrosation (Method C): Synthesis of intermediates (7): Amino compounds **(6)** (1 eq) were dissolved in DCM-Water (1.5-2) (0.1 M) and OXONE (2 eq) were added. The reaction mixture was stirred vigorously at r.t. overnight. The mixture was extracted twice with DCM, the organic layer was dried over anhydrous $MgSO_4$, filtered and concentrated in vacuum. The residue was purified by flash chromatography (silica gel) to afford pure nitroso compounds **(7).**

Methyl 3'-nitroso-[1,1'-biphenyl]-4-carboxylate (7a)

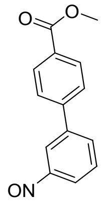

According to method C, from 2.53 g of commercial methyl 3'-amino-[1,1'-biphenyl]-4-carboxylate **(6a)**, 869 mg of **(7a)** were obtained (32% yield). ^{1}H-NMR (400 MHz, DMSO-□6) δ 8.16 (d, *J* = 8.4 Hz, 2H), 8.13 (t, *J* = 1.9 Hz, 1H), 7.98 (ddd, *J* = 7.8, 1.9, 1.1 Hz, 1H), 7.94 (ddd, *J* = 7.8, 1.9, 1.1 Hz, 1H), 7.73 (d, *J* = 8.4 Hz, 2H), 7.73 (t, *J* = 7.8 Hz, 1H), 3.96 (s, 3H). ^{13}C-NMR

(101 MHz, $CDCl_3$) δ 166.85, 165.96, 143.69, 141.56, 133.95, 130.53, 130.12, 130.08, 127.33, 120.66, 119.71, 52.42.

Methyl 2-methyl-3'-nitroso-[1,1'-biphenyl]-4-carboxylate (7b)

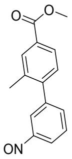

According to method C, from 322 mg of **(6b)**, 72 mg of **(7b)** were obtained (21% yield).^{1}H-NMR (400 MHz, $CDCl_3$) δ 8.01 (t, *J* = 1.8 Hz, 1H), 7.97–7.92 (m, 2H), 7.85 (t, *J* = 1.2 Hz, 1H), 7.73–7.66 (m, 2H), 7.34 (d, *J* = 7.9 Hz, 1H), 3.95 (s, 3H), 2.34 (s, 3H). ^{13}C-NMR (101 MHz, $CDCl_3$) δ 167.05, 165.52, 144.53, 142.48, 135.88, 135.81, 131.93, 130.00, 129.96, 129.49, 127.45, 121.38, 120.18, 52.36, 20.49.

General Procedure for Azocompounds formation (Method D): Synthesis of compounds (10): A solution of a nitroso compounds **(7)** (1.1 eq) and an amines **(9)** (1 eq) in AcOH:DCM (0.1 M), was stirred at rt overnight. Then was concentrated under reduced pressure, and the residue was purified by reverse phase to provide the azocompounds **(10)**.

(+/-)-Methyl 3'-((4-(2-hydroxy-3,3-dimethylbutoxy)-2-methylphenyl)diazenyl)-[1,1'-biphenyl]-4-carboxylate ((+/-)-10b)

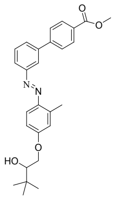

According to method D, from 112 mg of nitroso (**7a**) and 94 mg of amine (**(+/-)-9b**) in AcOH:DCM (0.04 M), 92 mg of (**(+/-)-10b**) (49% yield) were obtained. ^{1}H NMR (400 MHz, $CDCl_3$) δ 8.14 (d, *J* = 8.5 Hz, 2H), 8.14 (t, *J* = 1.9 Hz, 1H), 7.90 (ddd, *J* = 7.8, 1.9, 1.1 Hz, 1H), 7.75 (d, *J* = 8.5 Hz, 2H), 7.74 (d, *J* = 9.2 Hz, 1H), 7.69 (ddd, *J* = 7.8, 1.9, 1.1 Hz, 1H), 7.58 (t, *J* = 7.8 Hz, 1H), 6.87 (d, *J* = 2.8 Hz, 1H), 6.82 (dd, *J* = 8.9, 2.8 Hz, 1H), 4.18 (dd, *J* = 9.2, 2.4 Hz, 1H), 3.95 (s, 3H), (t, *J* = 9.2 Hz, 4H), 3.72 (dd, *J* = 9.2, 2.4 Hz, 1H), 2.75 (s, 3H), 1.04 (s, 9H). ^{13}C-NMR (101 MHz, $CDCl_3$) δ 167.10, 161.39, 153.75, 145.43, 145.16, 141.28, 141.07, 130.35,

129.74, 129.38, 129.01, 127.28, 122.16, 122.06, 117.30, 116.23, 113.22, 77.37, 69.84, 52.33, 33.81, 26.21, 18.02. HRMS (ESI+): m/z calcd for $C_{27}H_{31}N_2O_4^+$ $[M + H]^+$ = 447.2284; found 447.2268.

(-)-(*R*)-Methyl 3'-((4-(2-hydroxy-3,3-dimethylbutoxy)-2-methylphenyl)diazenyl)-[1,1'-biphenyl]-4-carboxylate ((-)-10b)

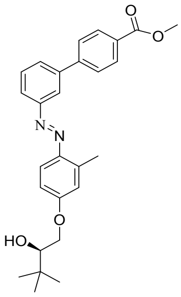

According to method D/Chiral 2, from 62 mg of nitroso **(7a)** and 52 mg of amine **((-)-9b)** in AcOH:DCM (0.04 M), 64 mg of **((-)-10b)** (62% yield) were obtained in a 89% ee

According to method Chiral-1 from 6.8 mg of racemic **((+/-)-10b)**, 1.1 mg of **((-)-10b)** (32% yield) were obtained in a 100% ee. [α] D_{20} = -12 (c 0.1, $CHCl_3$)

(+)-(*S*)-Methyl 3'-((4-(2-hydroxy-3,3-dimethylbutoxy)-2-methylphenyl)diazenyl)-[1,1'-biphenyl]-4-carboxylate ((+)-10b)

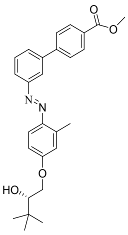

According to method D/Chiral-2, from 59 mg of nitroso **(7a)** and 50 mg of amine **((+)-9b)** in AcOH:DCM (0.04 M), 57 mg of **((+)-10b)** (57% yield) were obtained in a 89% ee.

According to method Chiral-1, from 6.8 mg of racemic **((+/-)-10b)**,1.1 mg of **((+)-10b)** (32% yield) were obtained in a 98% ee. [α] D_{20} = +8 (c 0.1, $CHCl_3$)

(+/-)-1-(4-((4'-(Hydroxymethyl)-[1,1'-biphenyl]-3-yl)diazenyl)-2-methylphenoxy)-3,3-dimethylbutan-2-ol ((+/-)-10a)

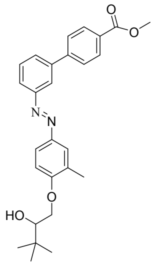

According to method D, from 31 mg of nitroso **(7a)** and 26 mg of amine **((+/-)-9a)** in AcOH:DCM (0.04 M), 57 mg of **((+/-)-10a)** (56% yield) were obtained. ^{1}H NMR (400 MHz, $CDCl_3$) δ 8.02 (d, *J* = 8.5 Hz, 2H), 8.02 (t, *J* = 1.9 Hz, 1H), 7.78 (ddd, *J* = 7.9, 2.0, 1.1 Hz, 1H), 7.71 (dd, *J* = 8.4, 2.4 Hz, 1H), 7.69 (d, *J* = 2.4 Hz, 1H), 7.64 (d, *J* = 8.5 Hz, 2H), 7.58 (ddd, *J* = 7.8, 1.9, 1.1 Hz, 1H), 7.47 (t, *J* = 7.8 Hz, 1H), 6.84 (d, *J* = 8.4 Hz, 1H), 4.10 (dd, *J* = 9.2, 2.6 Hz, 1H), 3.87 (t, *J* = 9.2 Hz, 1H), 3.83 (s, 3H), 3.65 (dt, *J* = 9.2, 2.6 Hz 1H), 2.21 (s, 3H), 0.93 (s, 9H). ^{13}C-NMR (101 MHz, Chloroform-d) δ 166.98. 159.70, 153.43, 146.88, 145.11, 141.08, 130.32, 129.77, 129.37, 129.12, 127.77, 127.30, 124.39, 124.08, 122.39, 121.53, 111.00, 77.36, 69.96, 52.33, 33.85, 26.22, 16.67. HRMS (ESI+): m/z calcd for $C_{27}H_{31}N_2O_4^+$ $[M + H]^+$ = 447.2284; found 447.2279.

(+/-)-Methyl 3'-((4-(2-hydroxy-3,3-dimethylbutoxy)-2-methylphenyl)diazenyl)-2-methyl-[1,1'-biphenyl]-4-carboxylate ((+/-)-10d)

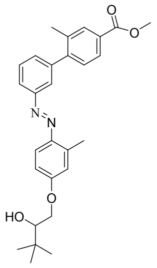

According to method D, from 72 mg of nitroso **(7b)** and 57 mg of amine **((+/-)-9b)** in AcOH:DCM (0.04 M), 39 mg of **((+/-)-10d)** (33% yield) were obtained.^{1}H NMR (400 MHz, $CDCl_3$) δ 7.99 (s, 1H), 7.93 (dd, *J* = 8.0, 1.2 Hz, 1H), 7.89 (ddd, *J* = 8.0, 1.6, 1.2 Hz, 1H), 7.84 (t, *J* = 1.6 Hz, 1H), 7.73 (d, *J* = 9.0 Hz, 1H), 7.56 (t, *J* = 8.0 Hz, 1H), 7.39 (dt, *J* = 8.0, 1.6 Hz, 2H), 7.38 (d, *J* = 8.0, 1H), 6.85 (d, *J* = 2.8 Hz, 1H), 6.81 (dd, *J* = 9.0, 2.8 Hz, 1H), 4.17 (dd, *J* = 9.0, 2.8 Hz, 1H), 3.95 (s, 3H), 3.94 (t, *J* = 9.0 Hz, 1H), 3.72 (dd, *J* = 9.0, 2.4 Hz, 1H), 2.72 (s, 3H), 2.37 (s, 3H), 1.04 (s, 9H). ^{13}C-NMR (101 MHz, $CDCl_3$) δ 167.28, 161.33, 153.16, 146.12, 145.34, 142.03. 141.27, 135.92, 131.74, 130.07, 129,35, 129.10, 127.24, 123.32, 121.87,

117.75, 117.17, 117.25, 113.20, 77.37, 52.28, 33.80, 26.59, 26.21, 20.63, 17.98. HRMS (ESI+): m/z calcd for $C_{28}H_{33}N_2O_4^+$ $[M + H]^+$ = 461.2435; found 447.2440.

<u>(+/-)-Methyl 3'-((4-(2-hydroxy-3,3-dimethylbutoxy)-3-methylphenyl)diazenyl)-2-methyl-[1,1'-biphenyl]-4-carboxylate</u> ((+/-)-10c)

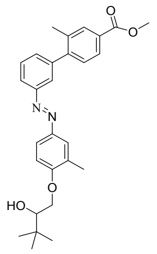

According to <u>method D</u>, from 62 mg of nitroso **(7b)** and 49 mg of amine **((+/-)-9a)** in AcOH:DCM (0.04 M), 37 mg of **((+/-)-10c)** (33% yield) were obtained.

^{1}H NMR (400 MHz, $CDCl_3$) δ 7.98 (d, *J* = 1.8 Hz, 1H), 7.92 (dd, J = 8.0, 1.2 Hz, 1H), 7.89 (ddd, *J* = 8.0, 1.8, 1.2 Hz, 1H), 7.84 (t, *J* = 1.8 Hz, 1H), 7.81 (dd, *J* = 8.4, 2.8 Hz, 1H), 7.79 (d, *J* = 2.8 Hz, 1H), 7.56 (t, *J* = 8.0 Hz, 1H), 7.39 (dt, *J* = 8.0, 1.2 Hz, 1H), 7.37 (d, *J* = 8.0 Hz, 1H), 6.95 (d, *J* = 8.4 Hz, 1H), 4.21 (dd, *J* = 9.2, 2.4 Hz, 1H), 3.99 (t, J = 9.2 Hz, 1H), 3.95 (s, 4H), 3.77 (brd, *J* = 9.2 Hz, 1H), 2.36 (s, 3H), 2.32 (s, 3H), 1.05 (s, 9H). ^{13}C NMR (101 MHz, $CDCl_3$) δ 167.30, 159.63, 152.88, 146.88, 146.06, 142.03, 135.90, 131.70, 130.87, 130.06, 129.32, 129.10, 127.74, 127.21, 124.33, 124.06, 123.01, 121.85, 110.98, 77.36, 69.94, 52.28, 33.84, 26.22, 20.61, 16.66. . HRMS (ESI+): m/z calcd for $C_{28}H_{33}N_2O_4^+$ $[M + H]^+$ = 461.2440; found 461.2432

General Procedure for Ester Hydrolysis (Method E): Synthesis of Final compound type (11): To a solution of ester compound type **(10)** (1 eq) in THF-MeOH (3:1) (0.05 M) was added a solution of LiOH 1M (10 eq). The mixture was stirred at r.t. for overnight, then was acidified with HCl 1N to pH~4-5. Finally, it was concentrated in vacuo and purified by Reverse Phase yielding the acid compound type **(11)**.

<u>(+/-)-3'-((4-(2-Hydroxy-3,3-dimethylbutoxy)-2-methylphenyl)diazenyl)-[1,1'-biphenyl]-4-carboxylic acid</u> ((+/-)-11b)

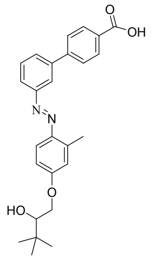

According to <u>method E</u>, from 49 mg of ester **((+/-)-10b)**, 36 mg of **((+/-)-11b)** (76% yield) were obtained

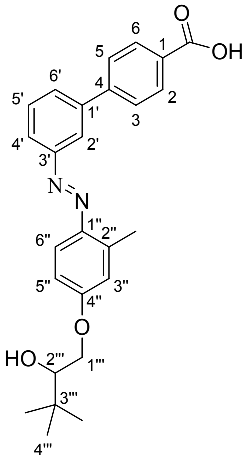

^{1}H NMR (400 MHz, $CDCl_3$) δ 8.28 – 8.20 (m, 2H, C(2)H, C(6)H), 8.16 (t, *J* = 1.9 Hz, 1H, C(2')H), 7.92 (dt, *J* = 8.0, 1.4 Hz, 1H, C(4')H), 7.83 – 7.77 (m, 2H, C(3)H, C(5)H), 7.75 (d, *J* = 8.9 Hz, 1H, C(6'')H), 7.71 (dt, *J* = 7.7, 1.3 Hz, 1H, C(6')H), 7.60 (t, *J* = 7.8 Hz, 1H, C(5')H), 6.87 (d, *J* = 2.7 Hz, 1H, C(3'')H), 6.83 (dd, *J* = 8.9, 2.8 Hz, 1H, C(5'')H), 4.19 (dd, *J* = 9.2, 2.5 Hz, 1H, C(1''')H), 3.96 (t, *J* = 9.0 Hz, 1H, C(2''')H), 3.73 (dd, *J* = 8.8, 2.4 Hz, 1H, C(1''')H'), 2.76 (s, 3H, CH_3), 1.05 (s, 9H, C(4''')H_3, 2CH_3). **^{13}C NMR** (101 MHz, $CDCl_3$) δ 171.34 (COOH), 161.43 (C(4'')), 153.74 (C(3')), 146.02 (C(1')), 145.41 (C(2'')), 141.33 (C(1'')), 140.94 (C(4)), 130.99 (C(2), C(6)), 129.79 (C(5')), 129.04 (C(6')), 128.45 (C(1)), 127.41 (C(3), C(5)), 122.28 (C(4'), 122.14 C(2'), 117.34 (C(6'')), 116.25 (C(3'')), 113.24 (C(5'')), 77.41 (C(2''')), 69.83 (C(1''')), 33.82 (C(3''')), 26.22(C(4'''), 2CH_3), 18.03 (CH_3). HRMS (ESI+): m/z calcd for $C_{26}H_{29}N_2O_4^+$ [M + H]$^+$ = 433,2122; found 433.2124

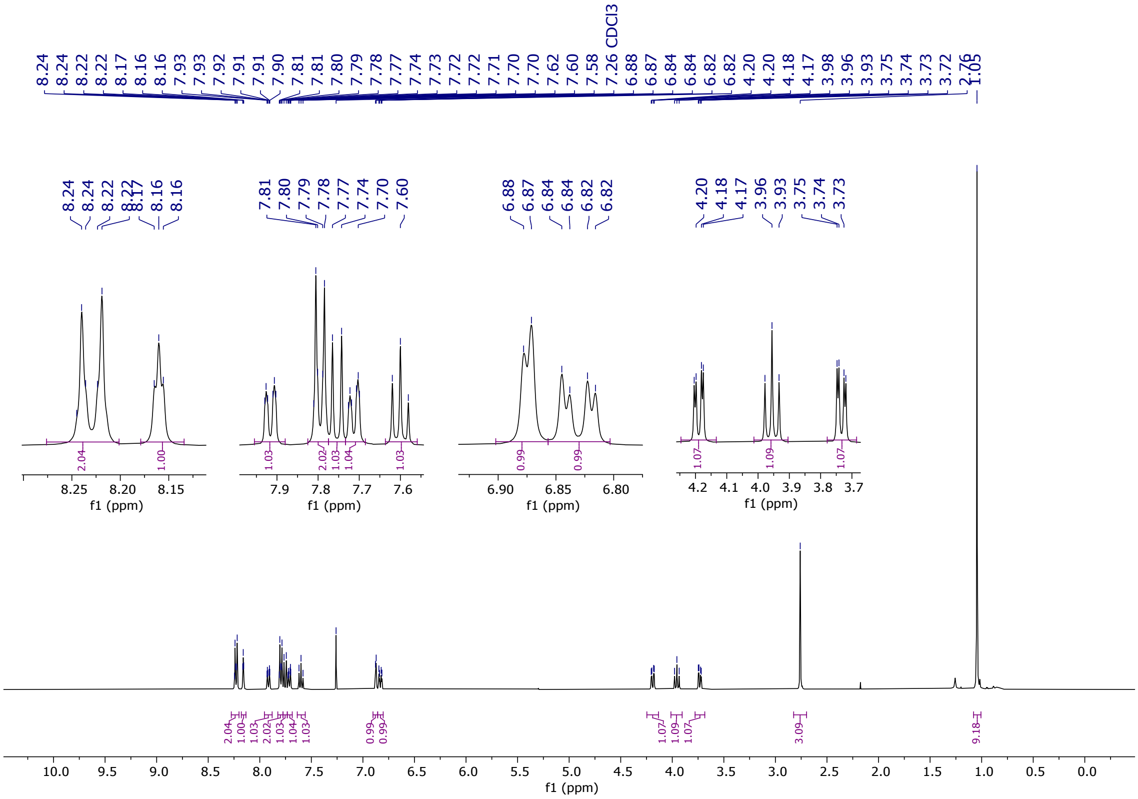

^{1}**H NMR** (400 MHz, $CDCl_3$) of compound **(+/-)-11b**.

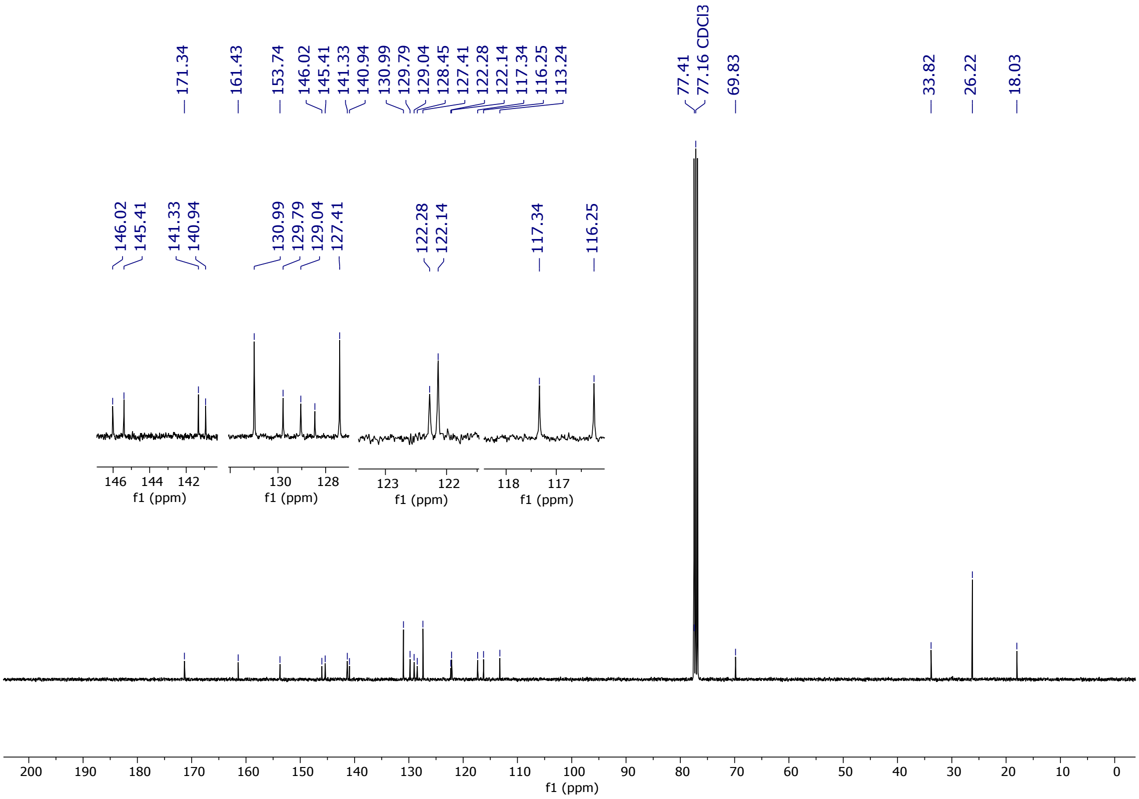

^{13}**C NMR** (101 MHz, $CDCl_3$) of compound **(+/-)-11b**.

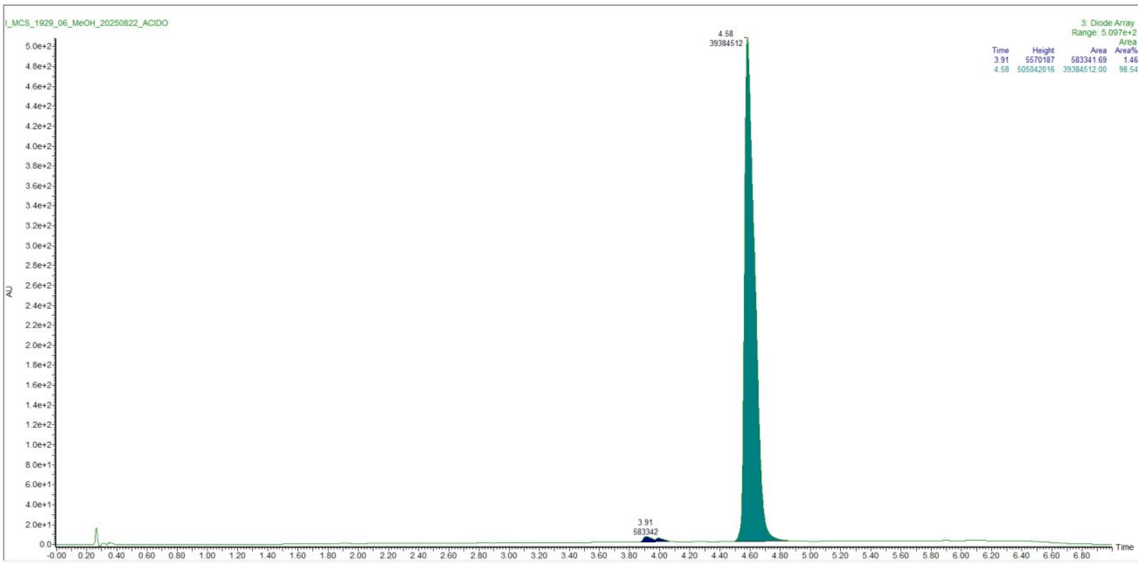

Time (min)	Height	Area	Area(%)	identification
3.91	5570187	583341.69	1.46	*trans*-(+/-)-11b
4.58	505042016	39384512.00	98.54	*cis*-(+/-)-11b

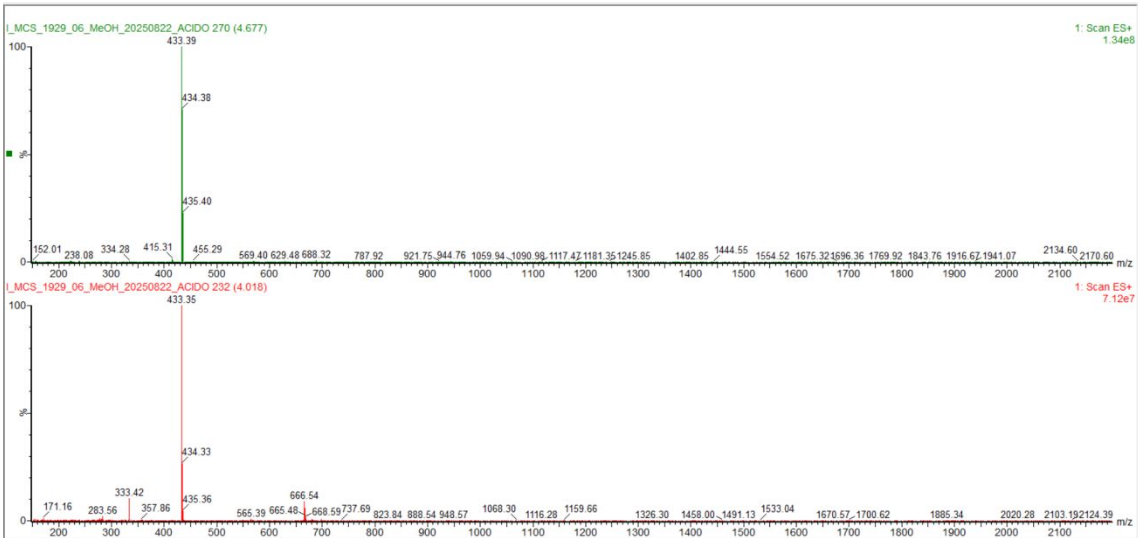

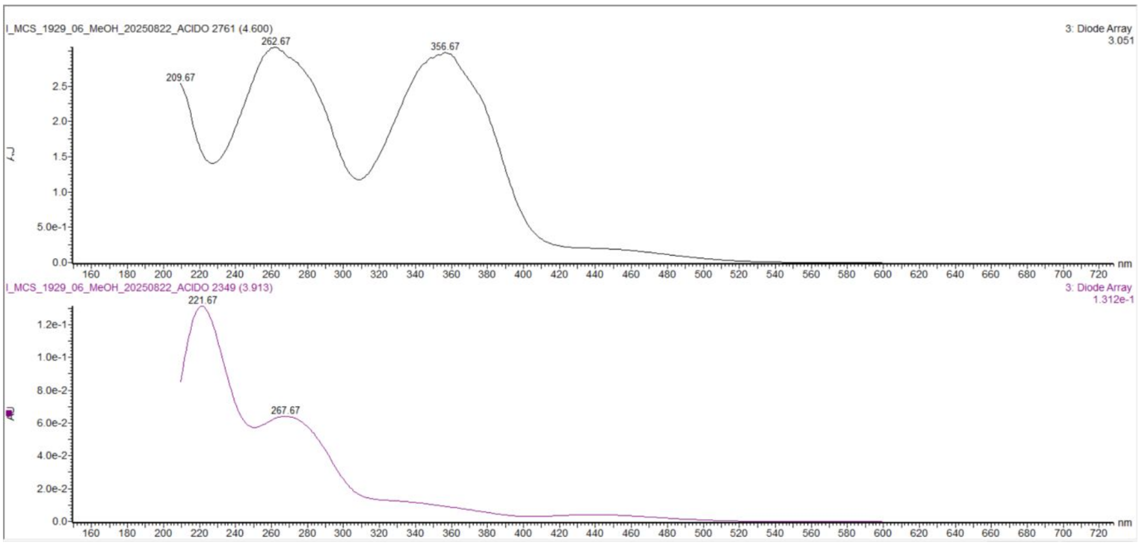

(-)-(*R*) 3'-((4-(2-Hydroxy-3,3-dimethylbutoxy)-2-methylphenyl)diazenyl)-[1,1'-biphenyl]-4-carboxylic acid((-)-11b)

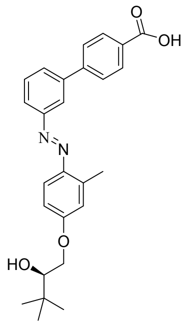

According to method E/Chiral 3, from 3.98 mg of ester **((-)-10b)**, 3.17 mg of **((-)-11b)** (82% yield) were obtained 98% ee. [α] D_{20} = -15 (c 0.2, MeOH).

(+)-(*S*) 3'-((4-(2-Hydroxy-3,3-dimethylbutoxy)-2-methylphenyl)diazenyl)-[1,1'-biphenyl]-4-carboxylic acid ((+)-11b)

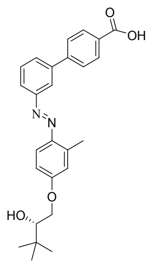

According to method E/ Chiral 3, from 3.5 mg of ester **((+)-10b)**, 2.66 mg of **((+)-11b)** (78% yield) were obtained. 98% ee. [α] D_{20} = +9 (c 0.2, MeOH).

(+/-)-3'-((4-(2-Hydroxy-3,3-dimethylbutoxy)-3-methylphenyl)diazenyl)-[1,1'-biphenyl]-4-carboxylic acid ((+/-)-11a)

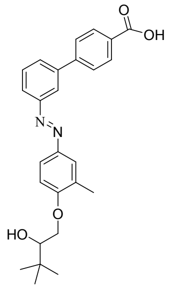

According to method E, from 7.0 mg of ester **((+/-)-10a)**, 6.5 mg of **((+/-)-11a)** (96% yield) were obtained. ^{1}H NMR (400 MHz, CD_3OD) δ 8.16-8.14 (m, 1H), 8.14 (d, *J* = 8.4 Hz, 2H), 7.89 (ddd, *J* = 8.0, 1.6, 1.2 Hz, 1H), 7.85-7.80 (m, 2H), 7.82 (d, *J* = 8.4 Hz, 2H), 7.79 (ddd, *J* = 8.0, 1.6, 1.2 Hz, 1H), 7.63 (t, *J* = 8.0 Hz, 1H), 7.08 (d, *J* = 8.6 Hz, 1H), 4.27 (dd, *J* = 10.1, 2.8 Hz, 1H), 4.02 (dd, *J* = 10.1, 7.8 Hz, 1H), 3.69 (dd, *J* = 7.8, 2.8 Hz, 1H), 2.34 (s, 3H), 1.04 (s, 9H). ^{13}C NMR (101 MHz, CD_3OD) δ 169.7, 161.7, 154.7, 147.8, 146.1, 142.4, 131.5, 131.3, 130.9, 130.1, 129.0, 128.1, 125.2, 124.9, 123.1, 122.1, 112.0, 78.5, 71.4, 35.2, 26.6, 16.7. HRMS (ESI+): m/z calcd for $C_{26}H_{29}N_2O_4^+$ $[M + H]^+$ = 433.2127; found 433.2142.

(+/-)-3'-((4-(2-Hydroxy-3,3-dimethylbutoxy)-2-methylphenyl)diazenyl)-2-methyl-[1,1'-biphenyl]-4-carboxylic acid ((+/-)-11d))

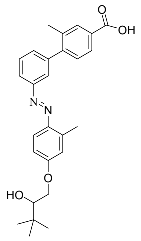

According to method E, from 7.0 mg of ester **((+/-)-10d)**, 6.5 mg of **((+/-)-11d)** (96% yield) were obtained. ^{1}H-NMR (400 MHz, CD_3OD) δ 7.97 (d, *J* = 1.8 Hz, 1H), 7.91 (dd, *J* = 7.8, 1.8 Hz, 1H), 7.87 (ddd, *J* = 7.8, 2.0, 1.2 Hz, 1H), 7.80 (t, *J* = 1.8 Hz, 1H), 7.72 (d, *J* = 9.0 Hz, 1H), 7.60 (t, *J* = 7.8 Hz, 1H), 7.43 (dt, J = 7.8, 1.2 Hz, 1H), 7.36 (d, *J* = 7.8 Hz, 1H), 6.95 (d, *J* = 2.8 Hz, 1H), 6.87 (dd, *J* = 9.0, 2.8 Hz, 1H), 4.22 (dd, *J* = 9.9, 2.6 Hz, 1H), 3.95 (dd, *J* = 9.9, 8.2 Hz, 1H), 3.63 (dd, *J* = 8.2, 2.6 Hz, 1H), 2.71 (s, 3H), 2.35 (s, 3H), 1.02 (s, 9H). ^{13}C-NMR (101 MHz, CD_3OD) δ 169.32, 161.99, 153.05, 145.36, 144.79, 142.12, 141.00, 135.29, 131.34, 130.95, 130.41, 129.36, 128.82, 126.95, 122.58, 121.18, 116.57, 115.67, 112.85, 77.04, 69.77, 33.66, 25.16, 19.23, 16.48.

(+/-)-3'-((4-(2-Hydroxy-3,3-dimethylbutoxy)-3-methylphenyl)diazenyl)-2-methyl-[1,1'-biphenyl]-4-carboxylic acid. ((+/-)-11c)

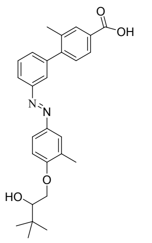

According to method E, from 10.0 mg of ester **((+/-)-10c)**, 1.5 mg of **((+/-)-11c)** (15% yield) were obtained.^{1}H-NMR (400 MHz, CD_3OD) δ 7.99 (s, 1H), 7.93 (dd, *J* = 8.0, 1.2 Hz, 1H), 7.88 (ddd, *J* = 8.0, 1.8, 1.2 Hz, 1H), 7.83–7.76 (m, 3H), 7.61 (t, *J* = 8.0 Hz, 1H), 7.45 (dt, *J* = 8.0, 1.4 Hz, 1H), 7.38 (d, *J* = 8.0 Hz, 1H), 7.07 (d, *J* = 8.6 Hz, 1H), 4.27 (dd, *J* = 10.1, 2.8 Hz, 1H), 4.01 (dd, *J* = 10.1, 7.8 Hz, 1H), 3.68 (dd, *J* = 7.8, 2.8 Hz, 1H), 2.36 (s, 3H), 2.33 (s, 3H), 1.04 (s, 9H). ^{13}C-NMR (101 MHz, CD_3OD) δ 172.38, 164.19, 156.65, 150.33, 149.76, 145.98, 139.44, 135.31, 134.42, 133.71, 133.44, 132.79, 131.47, 130.95, 127.68, 127.45, 126.24, 125.20, 114.53, 78,49, 73.91, 37.70, 29.11, 23.15, 19.22. HRMS (ESI+): m/z calcd for $C_{27}H_{31}N_2O_4^+$ $[M + H]^+$ = 447.2284; found 447.2260.

General Procedure for Ester Reduction (Method F): Synthesis of compounds (12): To a stirred solution of compounds **(10)** (1 eq) in anhydrous THF (0.1 M to 0.01M), LAH (4 eq) was added, in small portions, and the reaction mixture was stirred at r.t. for 3h. Then, the excess of LAH was destroyed with sodium sulphate decahydrate, filtered and evaporated to dryness, obtaining the compounds **(12)**.

(+/-)-1-(4-((4'-(Hydroxymethyl)-[1,1'-biphenyl]-3-yl)diazenyl)-3-methylphenoxy)-3,3-dimethylbutan-2-ol ((+/-)-12b)

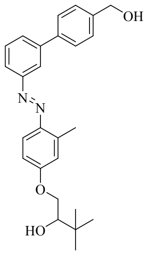

According to method F, from 15.0 mg of ester **((+/-)-10b)**, 2.2 mg of **((+/-)-12b)** (15% yield) were obtained once it was purified by reverse phase. ^{1}H-NMR (400 MHz, $CDCl_3$) δ 8.10 (t, *J* = 1.8 Hz, 1H), 7.83 (ddd, *J* = 8.0, 1.8, 1.2 Hz, 1H), 7.76 (d, *J* = 9.0 Hz, 1H), 7.72 (ddd, *J* = 8.0, 1.8, 1.2 Hz, 1H), 7.69 (d, *J* = 8.4 Hz, 2H), 7.59 (t, *J* = 8.0 Hz, 1H), 7.48 (d, *J* = 8.4 Hz, 2H), 6.96 (d, *J* = 2.8 Hz, 1H), 6.88 (dd, *J* = 9.0, 2.8 Hz, 1H), 4.68 (s, 2H), 4.58 (brs, 1H), 4.23 (dd, *J* = 9.9, 2.7 Hz, 1H), 3.96 (dd, *J* = 9.9, 8.2 Hz, 1H), 3.64 (dd, *J* = 8.2, 2.7 Hz, 1H), 2.75 (s, 3H), 1.03 (s, 9H). ^{13}C-NMR (101 MHz, CD_3OD) δ 162.00, 153.58, 145.86, 144.84, 141.98, 140.88, 139.69, 129.33, 128.57, 127.25, 126.80, 121.11, 120.58, 116.65, 115.72, 112.90, 77.18, 69.76, 63.49, 29.33, 25.16, 17.84. HRMS (ESI+): m/z calcd for $C_{26}H_{31}N_2O_3^+$ $[M + H]^+$ = 419.2335; found 419.2347.

(+/-)-1-(4-((4'-(Hydroxymethyl)-[1,1'-biphenyl]-3-yl)diazenyl)-2-methylphenoxy)-3,3-dimethylbutan-2-ol. ((+/-)-12a)

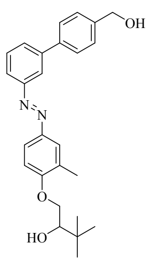

According to method F, from 2.0 mg of ester (**(+/-)-10a**), 1.9 mg of **((+/-)-12a)** (100% yield) were obtained once it was purified by reverse phase. ^{1}H NMR (400 MHz, CD_3OD) δ 8.10 (t, *J* = 1.8 Hz, 1H), 7.85–7.79 (m, 3H), 7.73 (ddd, *J* = 7.8, 1.8 1.2 Hz, 1H), 7.70 (d, *J* = 8.4 Hz, 2H), 7.59 (t, *J* = 7.8 Hz, 1H), 7.48 (d, *J* = 8.4 Hz, 2H), 7.08 (d, *J* = 8.6 Hz, 1H), 4.68 (s, 2H), 4.28 (dd, *J* = 10.1, 2.8 Hz, 1H), 4.02 (dd, *J* = 10.1, 7.8 Hz, 1H), 3.69 (dd, *J* = 7.8, 2.8 Hz, 1H), 2.03 (s, 3H), 1.04 (s, 9H). ^{13}C-NMR (101 MHz, $CDCl_3$) δ 159.59, 153.41, 146.93, 141.88, 140.40, 140.10, 129.61, 129.01, 127.73, 127.66, 127.55, 124.29, 124.06, 121.49, 121.45, 110.99, 77.36, 69.94, 65.27, 33.85, 26.22, 16.67. HRMS (ESI+): m/z calcd for $C_{26}H_{31}N_2O_3^+$ $[M + H]^+$ = 419.2335; found 419.2357.

(+/-)-1-(4-((4'-(Hydroxymethyl)-2'-methyl-[1,1'-biphenyl]-3-yl)diazenyl)-3-methylphenoxy)-3,3-dimethylbutan-2-ol ((+/-)-12d)

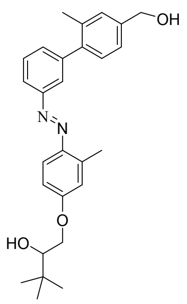

According to method F, from 14.0 mg of ester **((+/-)-10d)**, 5.3 mg of **((+/-)-12d)** (40% yield) were obtained once it was purified by reverse phase. ^{1}H-NMR (400 MHz, $CDCl_3$) δ 7.75 (ddd, *J* = 8.0, 1.8, 1.2 Hz, 1H), 7.71 (t, *J* = 1.8 Hz, 1H), 7.60 (d, *J* = 8.8 Hz, 1H), 7.42 (t, *J* = 8.0 Hz, 1H), 7.27 (ddd, *J* = 8.0, 1.8, 1.2 Hz, 1H), 7.20-7.15 (m, 3H), 6.74 (d, *J* = 2.8 Hz, 1H), 6.70 (dd, *J* = 8.8, 2.8 Hz, 1H), 4.60 (s, 2H), 4.06 (dd, *J* = 9.2, 2.4 Hz, 1H), 3.82 (t, *J* = 9.2 Hz, 1H), 3.59 (dd, *J* = 9.2, 2.4 Hz, 1H), 2.60 (s, 3H), 2.22 (s, 3H), 0.92 (s, 9H). ^{13}C-NMR (101 MHz, CD_3OD) δ 161.92, 153.00, 144.90, 142.86, 140.89, 140.74, 140.18, 134.95, 130.72, 129.30, 128.72, 128.62, 124.27, 122.81, 120.70, 116.53, 115.66, 112.83, 77.04, 69.76, 63.59, 33.66, 25.16, 19.23, 16.46. HRMS (ESI+): m/z calcd for $C_{26}H_{31}N_2O_3^+$ $[M + H]^+$ = 419.2335; found 419.2333.

(+/-)-1-(4-((4'-(Hydroxymethyl)-2'-methyl-[1,1'-biphenyl]-3-yl)diazenyl)-2-methylphenoxy)-3,3-dimethylbutan-2-ol ((+/-)-12c)

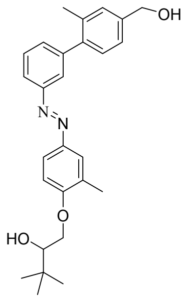

According to method F, from 10.0 mg of ester **((+/-)-10c)**, 2.4 mg of **((+/-)-12c)** (25% yield) were obtained once it was purified by reverse phase. ^{1}H NMR (400 MHz, CD_3OD) δ 7.84 (ddd, *J* = 7.8, 1.8, 1.0 Hz, 1H), 7.80 (dd, *J* = 8.6, 2.3 Hz, 1H), 7.77-7.75 (m, 2H), 7.57 (t, *J* = 7.8 Hz, 1H), 7.41 (dt, J = 7.6, 1.2 Hz, 1H), 7.31 (s, 1H), 7.27-7.23 (m, 2H), 7.07 (d, *J* = 8.6 Hz, 1H), 4.64 (s, 2H), 4.26 (dd, *J* = 10.0, 2.9 Hz, 1H), 4.01 (dd, *J* = 10.0, 7.8 Hz, 1H), 3.68 (dd, *J* = 7.8, 2.8 Hz, 1H), 2.33 (s, 3H), 2.31 (s, 3H), 1.04 (s, 9H). 13C NMR (101 MHz, CD_3OD) δ 161.57, 154.04, 147.81,

144.29, 142.13, 141.55, 136.35, 132.23, 130.72, 130.11, 130.01, 128.91, 125.65, 125.04, 124.89, 124.03, 122.01, 111.98, 78.49, 71.36, 64.99, 35.16, 26.57, 20.62, 16.68.

HRMS (ESI+): m/z calcd for $C_{27}H_{33}N_2O_3^+$ $[M + H]^+$ = 433.2486; found 433.2489.

2. Photochemical characterization.

UV-Vis spectroscopy.

A 50 μM solution of each compound in DMSO was dispensed into a transparent 96-well plate (200 μL per well). The absorption spectra were recorded from 300 to 600 nm with 2 nm fixed intervals using a Tecan Spark 20M Multimode microplate reader. Initial measurements were performed in the dark, followed by spectra acquired immediately after each 3 min period of light irradiation at different wavelengths using 96-well LED array plates (LEDA Teleopto). Theoretical absorbance spectra corresponding to the pure cis isomer (100%) of each compound were calculated in order to obtain the theoretical percentage of cis isomer at each wavelength, according to the method described by Ernst Fischer (1967). Thermal relaxation kinetics of the experimental cis isomers were assessed in quartz cuvettes (10 mm optical path) containing freshly prepared 50 μM solutions of the compounds in DMSO. Dark state absorbance at 365 nm was recorded at 37 °C using a ThermoFisher Evolution 350 UV-Vis spectrophotometer. Samples were then irradiated for 3 minutes at 365 nm with a CoolLED pE-4000 light sourceto induce trans → cis photoisomerization.. Absorbance at 365 nm was subsequently monitored over 48 h: every 15 min during the first 24 h, and every 60 min during the following 24 h. Relaxation half-lives were determined by fitting the absorbance-time data to a one-phase association exponential model using GraphPad Prism v10.4.1.

Compound 11b was further investigated to assess photostability under repeated photoswitching cycles. Wavelengths corresponding to the maximal absorption of the cis and trans isomers (365 and 460 nm) were selected for bidirectional photoisomerization. A 60 μM solution of compound in assay buffer (see Section 3) was prepared in a quartz cuvette. Absorbance at 365 nm was first recorded in the dark (integration time: 1 seg; bandwidth: 2 nm). The sample was then subjected to alternating 3 min irradiations at 365 and 460 nm for six consecutive cycles using the CoolLED pE-4000 light source to induce trans ↔ cis conversion. Absorbance was recorded immediately after each illumination cycle.

Photostationary state (PSS) determination. In a transparent vial, solutions of the compounds were prepared at a concentration of 60 μM from a 10 mM stock in DMSO, using the assay buffer of pharmacological experiments for dilution. Prior to illumination, a volume of 500 μL was transferred to an amber vial for the "dark" sample. The same procedure was repeated after exposing the transparent vial containing the 60 μM solutions to the following light conditions: 365nm for 3 minutes and 525nm for 3 minutes. Of note, longer exposure times did not elicit a difference on isomeric proportions. For each sample, 20 μL was analyzed by HPLC-MS, and the isomer ratios were determined at the isosbestic point.

Isosbestic point determination. The solvent percentage for the elution of each isomer were calculated based on their retention times from HPLC analysis. Subsequently, 50 μM solutions were prepared from the 10 mM DMSO stock solution of each compound using the previously determined HPLC solvent mixture. The UV spectrum for each solution was recorded after illumination at 365 nm (30 seconds), at 400 nm (30 seconds), and at 525 nm (30 seconds). The spectra were overlaid to identify the isosbestic point of maximum intensity.

HPLC conditions. Analytical HPLC was performed on a Thermo Ultimate 3000SD (Thermo Scientific Dionex) coupled to a PDA detector and Mass Spectrometer LTQ XL ESI-ion trap (Thermo Scientific). Cromatographic separations were performed using a ZORBAX Eclipse Plus C18 (4.6 x 150mm; 3.5μm) column. The mobile phase consisted of an isocratic mixture of solvent A (water with 0.05% of formic acid) and solvent B (acetonitrile with 0.05% formic acid) at a 3:7 ratio for compounds 10a-d and a 2:3 ratio for compounds 11a-d and 12a-d. The flow rate was 0.9 mL/min, column temperature was fixed to 35°C and wavelengths from 210-600 nm were registered.

3. Pharmacology

The activity of all molecules was evaluated using an in-vitro luminescent proximity homogeneous assay by using the Alphascreen technology (Cat No. 6760619R, Perkin Elmer, now Revvity). In a transparent 96-well preplate, 10X concentrations of the compounds were prepared in assay buffer (0.1% bovine serum albumin, 2 mM 1,4-dithiothreitol, 25 mM HEPES and 100 mM NaCl; pH 7.3). LSN2148936 was used as positive control. Purified histidine-tagged VDR (44 μM), flag-RxRa (5.7 μM), biotin tagged TRAP220 transcriptional coactivator (500 μM), and the donor and acceptor beads from the AlphaScreen kit (2 μL/mL each), were diluted in assay buffer. In the dark, 6 μL/well of each test compound were pipetted on a white, shallow 96-well ProxiPlate™

(Perkin Elmer, now Revvity, Cat.No. 6006290) in duplicate. Immediately, 54 µL of the protein solution were added and the plate was covered and incubated for 30 min at room temperature in the dark. Meanwhile, the plate with the 10X dilutions was illuminated at 365 nm for 3 min in a 96-well DED array plate (LEDA Teleopto), to allow the photoconversion of the molecules to their *cis* configuration. The illuminated compounds were mixed with the protein solution and incubated as described above for the plate in the dark. After the incubation time, the counts of both, the dark and illuminated plates, were measured in a Tecan Spark 20M Multimode plate reader with defined settings (excitation: 680 nm, emission: 520-620 nm, excitation time: 100ms, integration time: 300 ms). Biotinylated-His6 peptide (PerkinElmer) was used as a quality control reagent for AlphaScreen® assays and to evaluate compound interaction with the method. The raw data were normalized to the effect of the buffer (0%) and the maximum response obtained with LSN2148936 (100%). Dose-response curves and EC50 values were calculated with Graphpad software. Each experiment was performed in triplicate, with 2 replicates per plate.

4. Hydrogen/deuterium exchange coupled to mass spectrometry (HDX-MS)

The HDX-MS experiments were performed with an in-house automated modular system interfaced with an Orbitrap Exploris 480 mass spectrometer.[1] Two inter-day experiments were conducted: one in the dark (first day) and one illuminated with 365 nm light (second day). Sample preparation and on-exchange reactions were performed on opaque plates (VWR® Real-Time PCR plates, 96-well, reference 83009-672). A Tube UV T8 EiKO F36T8/BLB-EI (36 W 1200 mm, Part N° BC5226) light source was positioned above the Tecan system used for sample preparation. The distance between the UV light and the opaque plates was 80 cm. The 365 nm light illuminated the sample preparation process and the on-exchange reaction on the second day. A 10 µM VDR-LBD protein stock solution was prepared in 20 mM HEPES buffer (with 150 mM NaCl, 10 mM D-L Methionine and 5 mM DTT) at pH 7.5. The compound **11b** was prepared at 10 mM in DMSO. The protein: compound-complex (1:10 molar ratio) was pre-incubated for 1h before the first on-exchange reaction. The HDX reaction was initiated by a 5-fold dilution of the protein stock solution, in the presence and absence of compound **11b**, in the corresponding Deuterated buffer at room temperature. Samples were prepared in parallel with three replicates of the following exchange times: Dmin, 10s, 30s, 90s, 270s, 810s, 2430s, 7290s and Dmax. At each time point, HDX reactions were quenched with a solution of 3 M Urea and 1% TFA (pH 2.5) followed by online proteolysis with an in-house Pepsin immobilized column and desalting through a Symmetry C8 (2.1x10 mm, 3.5 µm) trap column at 4 ^{0}C. The resulting proteolytic

peptide mixtures were separated by reversed-phase liquid chromatography on an analytical Acclaim C18 (2.1x100 mm, 2.2 μm) column with a linear gradient from 5 to 50% of acetonitrile in 0.3% of formic acid over 5 min at 200 μL/min and eluted into the mass spectrometer (Exploris 480, Thermo Scientific). HDX-MS data were processed with HDX Workbench software. A comprehensive CSV file with the full set of HDX data is provided at FigShare.

5. Photoisomerization Quantum Yield

The quantum yield of compound **11b** was found using procedures previously described.[2] A CoolLED pE-4000 light source and a liquid light guide (CoolLED Ltd., Andover, England) illuminated 200 or 300 μL of 60 μM compound **11b** in a quartz cuvette at 37C. To measure the quantum yield of the *trans*-to-*cis* conversion, 365 nm light (10 nm full width at half maximum) illuminated a sample of 100% *trans* **11b** from the top of the cuvette while absorbance measurements were taken at 365 nm through the quartz window (Figure S9). Initial and final concentrations of the compound were calculated from absorbance measurements using the Beer-Lambert law.[3] HPLC enabled estimation of the molar absorption coefficients needed to calculate concentrations of compound **11b**. HPLC measurements were taken after illuminating 50 μM **11b** for 3 minutes with 365, 405, 435, and 460 nm light and without illuminating 50 μM **11b**. Because azobenzene compounds reach a wavelength-dependent photostationary state after illumination for long periods,[2] these measurements revealed the equilibrium concentrations of *trans* and *cis* **11b**. Absorbance measurements were taken at 365 nm after the same illumination protocol used for the HPLC measurements, and the combination of absorbance and HPLC measurements a relationship between absorbance and concentrations of *trans* and *cis* **11b** (Figure S9). Estimates of molar absorptivity were found by sweeping across possible coefficient values and choosing the values that minimized the sum of squared percentage error between actual and predicted absorbances.

Detailed procedure

In their work on photoisomerization quantum yield, Stranius and Börjesson used an equation to show how the quantum yields of a molecule's two photoisomers impact the molecule's isomerization rate (Stranius & Börjesson, 2017). In the equation (reproduced below), $[X]$ is the molarity of isomer X, ϕ_X is the quantum yield of isomer X, I is the photon flux, β_X is the fraction of photons absorbed by isomer X, N_A is the Avogadro constant, V is the volume of the sample, and k_t is the rate of thermal relaxation. For the compounds in this study, isomer A is the *trans* isomer, and isomer B is the *cis* isomer.

$$\frac{d[A]}{dt} = -\frac{\phi_A \cdot I \cdot \beta_A(t)}{N_A \cdot V} + \frac{\phi_B \cdot I \cdot \beta_B(t)}{N_A \cdot V} + k_{t,B\rightarrow A}[B]$$

The final righthand term in the rate equation represents the rate of *cis*-to-*trans* thermal relaxation. Omitting this term in this study minimally impacted the accuracy of the equation because of the photothermal properties of the compounds in this study. Molecular half-life determines the rate of thermal relaxation, and the molecules in this study have half-lives of hours. The same molecules reached photostationary states within a few minutes of constant illumination, indicating a much faster rate of photoswitching than of thermal relaxation. Omitting the thermal relaxation term simplifies the isomerization rate equation to the form shown below.

$$\frac{d[A]}{dt} = -\frac{\phi_A \cdot I \cdot \beta_A(t)}{N_A \cdot V} + \frac{\phi_B \cdot I \cdot \beta_B(t)}{N_A \cdot V}$$

Finding the quantum yields of the two photoisomers using this simplified equation requires knowing the fraction of photons absorbed by each photoisomer of the compound under analysis. Stranius and Börjesson provided the following equations for the fraction of photons absorbed by the two photoisomers (Stranius & Börjesson, 2017).

$$\beta_A(t) = \frac{[A] \cdot \varepsilon_A}{[A] \cdot \varepsilon_A + [B] \cdot \varepsilon_B}(1 - 10^{-Abs(t)})$$

$$\beta_B(t) = \frac{[B] \cdot \varepsilon_B}{[A] \cdot \varepsilon_A + [B] \cdot \varepsilon_B}(1 - 10^{-Abs(t)})$$

Data gathered through HPLC and UV-Vis spectroscopy enabled the determination of isomer concentrations from absorbance measurements. In a sample consisting of the two photoisomers in solution, one can write an equation for absorbance using the Beer-Lambert law. In the equation, l is the photon path length.

$$Abs(t) = l \cdot \varepsilon_A \cdot [A] + l \cdot \varepsilon_B \cdot [B]$$

Fitting the absorbance equation to absorbance data from a sample yielded molar absorptivity values for the two photoisomers present in the sample. The molar absorptivities and the concentrations determined for the photoisomers through HPLC enabled calculation of the quantum yields for the two photoisomers. Determining the quantum yields involved another round of ordinary least squares regression. This regression seeks to minimize the error between the simplified isomerization rate equation and the first-order time derivative of an absorbance measurement taken on the sample under test. These regressions were performed in MATLAB.

6. Molecular Modeling and MM/GBSA and quantum mechanical calculation.

The energy analysis was done with Schrodinger's 2022.3 suite (Schrödinger, LLC, New York, NY) using standard conditions except where otherwise indicated. Ligand parameters were generated for OPLS4 using Schrodinger's force field builder tool for all ligands considered. Both the *cis* and *trans* isomers, of both enantiomers, of **11b** were generated and converted to 3D using ligprep. Reference compound LSN2148936 was prepared in the same way. The *cis* isomers of **11b** were found to be higher in energy than the *trans* using the OPLS4 forcefield after full conformational search of other degrees of freedom and using the water model incorporated in Macromodel, and these results were confirmed by geometry optimization using a B3LYP-D3/6-31G** quantum mechanical calculation (Table S3); thus, photoisomerization should be needed to form significant amounts of the *cis* isomer. These structures were docked to a GLIDE docking[4–6] model prepared from the 3B0T.pdb structure,[7] using standard conditions except that during protein preparation the water molecules 425 and 498, located near the cyclohexanediol moiety of the vitamin D3 analogue present in the structure, were retained, and non-default parameters were chosen as listed in Table S4. Docking was followed by MM/GBSA geometry optimization using the OPLS4 forcefield of both the ligand and the protein. The best docking scores and MM/GBSA binding energies[8] found for the top-scoring poses (by MMGBSA dG bind energy and by docking score) are reported for each ligand in Table S5, along with the comparable data for vitamin D3 itself, which docks in a pose superposed to 0.943A RMSD on the ligand present in 3B0T.pdb (Vitamin D3 and the ligand of 3B0T differ in only one heavy atom in the structure, a CH2 group in VD3 being replaced with an oxygen).

7. IL23-induced psoriatic-like animal model

Adult C57BL/6N (Envigo Rms Spain SL., Sant Feliu de Codines, Spain) male and female mice bred in the animal facility of University of Barcelona (Campus of Bellvitge) weighing 25–35 g were used. The University of Barcelona Committee on Animal Use and Care (CEEA) approved the protocol. Following the approved experimental protocol all animals were supervised daily to assess signs of adverse effects during treatment. A retrospective analysis of the protocol demonstrated that no corrective measures (i.e. use of analgesics) were needed. Animals were housed and tested in compliance with the guidelines provided by the Guide for the Care and Use of Laboratory Animals [15] and following the European Union directives (2010/63/EU). Mice were housed in groups of five in standard cages with ad libitum access to food and water and maintained under a 12 h dark/light cycle (starting at 7:30 AM), at 22 °C and 66% humidity

(standard conditions). All animal experimentation was carried out by a researcher blind to drug treatments.

The IL23-induced mouse model of psoriasis was performed using C57BL/6N mice, as previously described [5]. The experimental approach consisted in a 5 consecutive days protocol. Briefly, mice were anesthetized by the i.p. administration of a ketamine (100 mg/kg)/xylazine (10 mg/kg) mixture and ear thickness was measured using a digital calliper. Then recombinant mouse IL23 (500 ng) or PBS were i.d. injected into the left or right ears, respectively, by using a Hamilton syringe during three consecutive days (days 1, 2, and 3). At days 2, 3 and 4 animals were treated intraperitoneally (i.p.) with vehicle (saline, 0.9% w/v NaCl) or drugs (LSN2148936; 0.005 mg/kg or **11b**; 0.5 mg/Kg) 20 min before being anaesthetised. Light irradiation of the corresponding ear was conducted before IL-23 administration by using a custom-made 9 × 4 light-emitting diode (LED) matrix (12 × 9 cm) placed at a height of 8 cm above the animals' heads. Upon that approach, a light regime of 420 nm or 520 nm wavelength consisted continuous light-irradiation at 1.18 mW/cm2 or at 7.64 mW/cm2 intensity LED, respectively, was performed during 8 min. Contralateral ear was covered in order to be protected from the collateral light-irradiation.

Serum calcium measurement in mice

After the final day of the experimental protocol, mice were euthanized to measure serum calcium levels. A total of 30 serum samples were analyzed. To ensure a painless and ethical procedure, animals were euthanized by administering a lethal dose of ketamine/xylazine (100 mg/kg ketamine, 10 mg/kg xylazine, i.p.).

Blood samples were collected via cardiac puncture immediately after euthanasia and placed in BD Vacutainer® 4 mL LH lithium heparin tubes (68 IU, green cap, Ref: 368884) to prevent clotting. Samples were then centrifuged at 1500 g for 10 minutes at room temperature, and the plasma fraction was carefully transferred to new Eppendorf tubes. All samples were immediately frozen at -80°C until analysis to ensure sample stability and prevent degradation.

Calcium determination method

Total calcium (tCa) concentration was determined using the Arsenazo III colorimetric method (OSR 60117, Beckman Coulter, Ireland), a well-established technique for accurate calcium quantification. The analysis was performed using the AU480 automated chemistry analyzer

(Beckman Coulter, Germany) following the manufacturer's protocol. Calcium measurements were expressed in mg/dL. All results were analyzed to assess inter-sample variability. Samples were stored at -80˚C until analysis to minimize potential degradation.

Histochemistry

Tissue samples from mice were immediately frozen in liquid nitrogen and maintained at -80˚C until embedded in OCT (Sakura Tissue-Tek® OCT Compound, Ref: 4583). Samples were then cryosectioned at 5 μm thickness using a Leica cryostat (Leica CM1950, Leica Biosystems, Germany). Sample processing for hematoxylin and eosin (H&E) staining was performed according to standard procedures. Images were acquired using a Zeiss AXIO Observer 7 inverted fluorescence motorized XY microscope and analyzed with the FIJI open-source platform for biological image analysis.

The serum analyses were performed at the Clinical Biochemistry Service (Edifici V, UAB Campus, Bellaterra, Cerdanyola del Vallès, Spain).

Supplementary Tables

Table S1. Pharmacological activity of **11b** isomers.

Compound	Activity (pEC_{50})	Activity (E_{max})
**11b** Isomer 2	-7.11 ± 0.10	76 ± 3.00
**11b** Isomer 1	-7.53 ± 0.06	83 ± 2.85

Table S2. MMGBSA residue-based interaction energies (kcal/mol) for key residues in the best MMGBSA pose found for various ligands.

Ligand	R274	W286	H305	H397
VD3	-14.58	-11.39	-9.25	-9.16
LSN2148936	-16.18	-13.22	-11.29	-8.74
Cis S 11b	-14.56	-11.65	-12.17	-9.3
Trans S 11b	-14.84	-5.85	-9.73	-7.71

Table S3. Relative energies of *cis* and *trans* **11b** using various models.

Isomer	MM solution phase relative energy (kcal/mol)	Solution phase relative energy (kcal/mol)#	Gas phase relative energy (kcal/mol)#
Cis	16.20	5.58	4.64
Trans	0.0	0.0	0.0

B3LYP-D3/6-31G**

Table S4. GLIDE docking conditions applied.

MAX_ITERATIONS	300
MAXKEEP	8000
MAXREF	600
NENHANCED_SAMPLING	2
POSES_PER_LIG	10
POSTDOCK_NPOSE	100
RINGCONFCUT	4.5
SCORING_CUTOFF	150.0

Table S5. GLIDE Docking scores and MMGBSA dG Bind (binding energy, OPLS4) values for the best poses found according to each score.

Ligand	Docking Score (glide_gscore)	MMGBSA calculated binding energy (dG_bind) kcal/mol
Vitamin D3	-14.122	-116.27
Vitamin D3	-13.820	-124.08
LSN2148936	-12.110	-77.16
LSN2148936	-11.128	-90.47
11b *cis* S	-11.171	-88.74
11b *cis* S	-10.844	-90.69

11b *cis* R	-11.003	-86.08
11b *cis* R	-10.082	-88.23
11b *trans* S	-9.679	-75.58
11b *trans* S	-8.371	-79.20
11b *trans* R	-9.163	-68.97

Table S6. Proportions of *cis* and *trans* at the photostationary state in DMSO. Calculated using the absorbance spectrums in S1 to S3 and the method described by Ernst Fischer (1967).

Cmp		Wavelength						
		365	380	405	420	470	500	530
10a	%trans	6,30%	8,17%	39,58%	41,95%	70,85%	74,46%	79,44%
	%cis	93,70%	91,83%	60,42%	58,05%	29,15%	25,54%	20,56%
10b	%trans	2,74%	3,52%	34,00%	37,28%	65,11%	64,35%	68,03%
	%cis	97,26%	96,48%	66,00%	62,72%	34,89%	35,65%	31,97%
10c	%trans	3,00%	4,38%	38,86%	40,29%	69,47%	73,18%	78,66%
	%cis	97,00%	95,62%	61,14%	59,71%	30,53%	26,82%	21,34%
10d	%trans	3,70%	4,68%	37,78%	40,01%	70,18%	74,79%	79,97%
	%cis	96,30%	95,32%	62,22%	59,99%	29,82%	25,21%	20,03%
11a	%trans	5,48%	7,19%	39,46%	41,91%	70,20%	73,41%	78,78%
	%cis	94,52%	92,81%	60,54%	58,09%	29,80%	26,59%	21,22%
11b	%trans	3,13%	4,38%	36,36%	39,53%	68,70%	67,19%	70,86%
	%cis	96,87%	95,62%	63,64%	60,47%	31,30%	32,81%	29,14%
11c	%trans	2,78%	5,28%	38,64%	40,74%	68,51%	71,82%	77,30%
	%cis	97,22%	94,72%	61,36%	59,26%	31,49%	28,18%	22,70%
11d	%trans	3,21%	4,42%	38,58%	45,31%	71,89%	70,26%	74,41%
	%cis	96,79%	95,58%	61,42%	54,69%	28,11%	29,74%	25,59%
12a	%trans	2,94%	4,39%	36,36%	39,55%	66,94%	65,97%	69,80%
	%cis	97,06%	95,61%	63,64%	60,45%	33,06%	34,03%	30,20%
12b	%trans	3,81%	4,85%	37,70%	40,04%	70,39%	74,85%	80,28%
	%cis	96,19%	95,15%	62,30%	59,96%	29,61%	25,15%	19,72%
12c	%trans	3,05%	4,31%	40,26%	43,87%	71,05%	69,52%	73,13%
	%cis	96,95%	95,69%	59,74%	56,13%	28,95%	30,48%	26,87%
12d	%trans	3,08%	4,23%	36,23%	39,87%	67,86%	66,82%	70,53%
	%cis	96,92%	95,77%	63,77%	60,13%	32,14%	33,18%	29,47%

Supplementary Figures

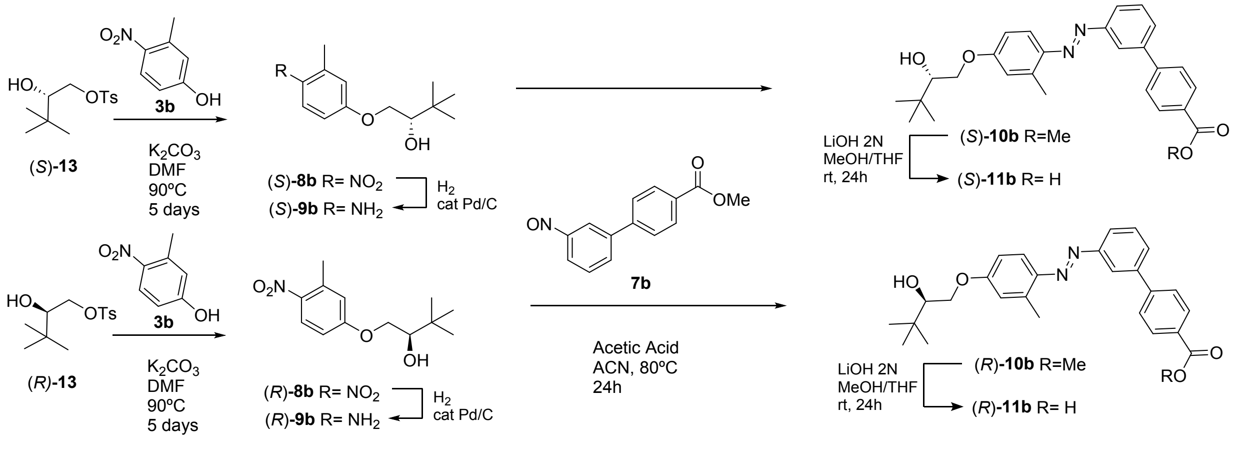

Scheme S1. Synthesis of enantiomeric nitroalcohols 8

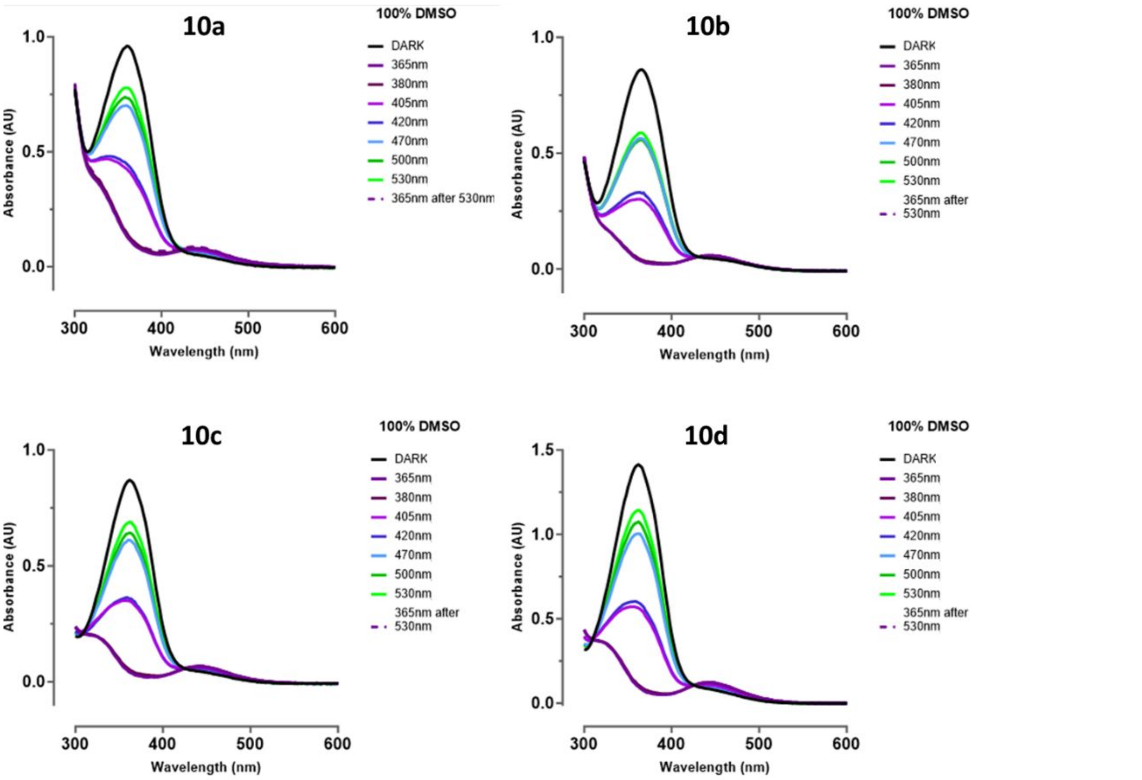

Figure S1. Photochemical properties of ester derivatives in DMSO. UV-Vis absorption spectra of compound 10a-d under different light conditions.

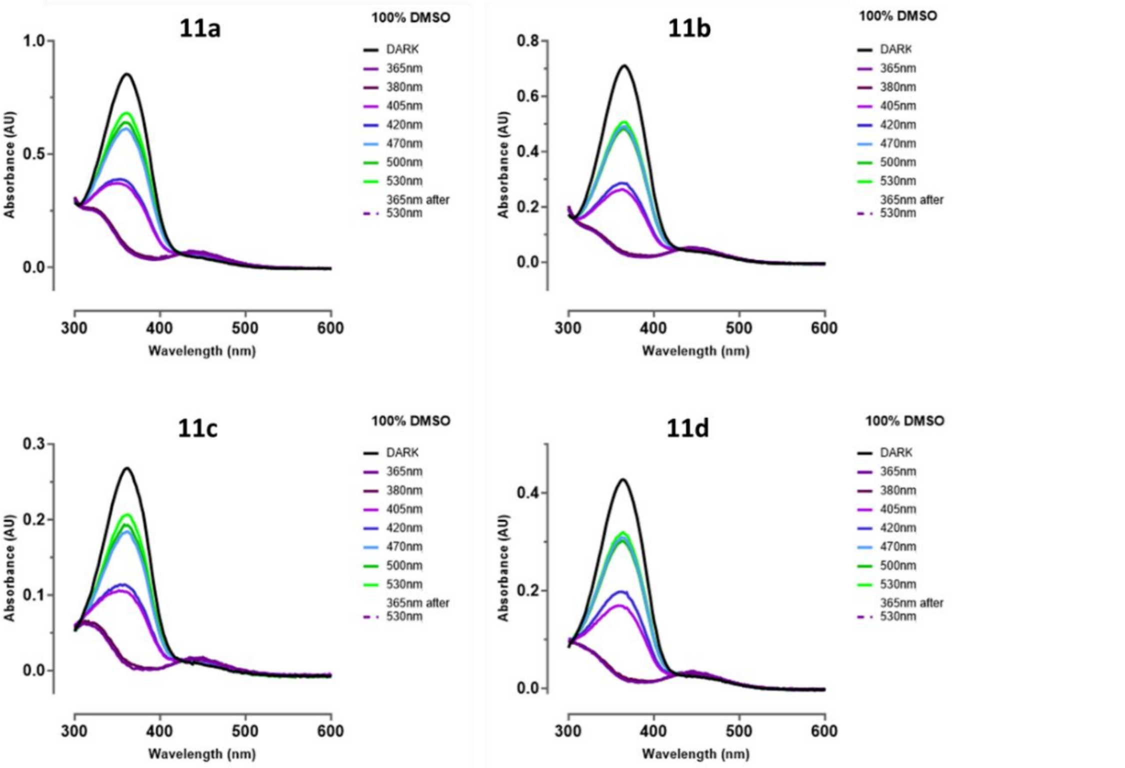

Figure S2. Photochemical properties of carboxylic acid derivatives in DMSO. UV-Vis absorption spectra of compound 11a-d under different light conditions.

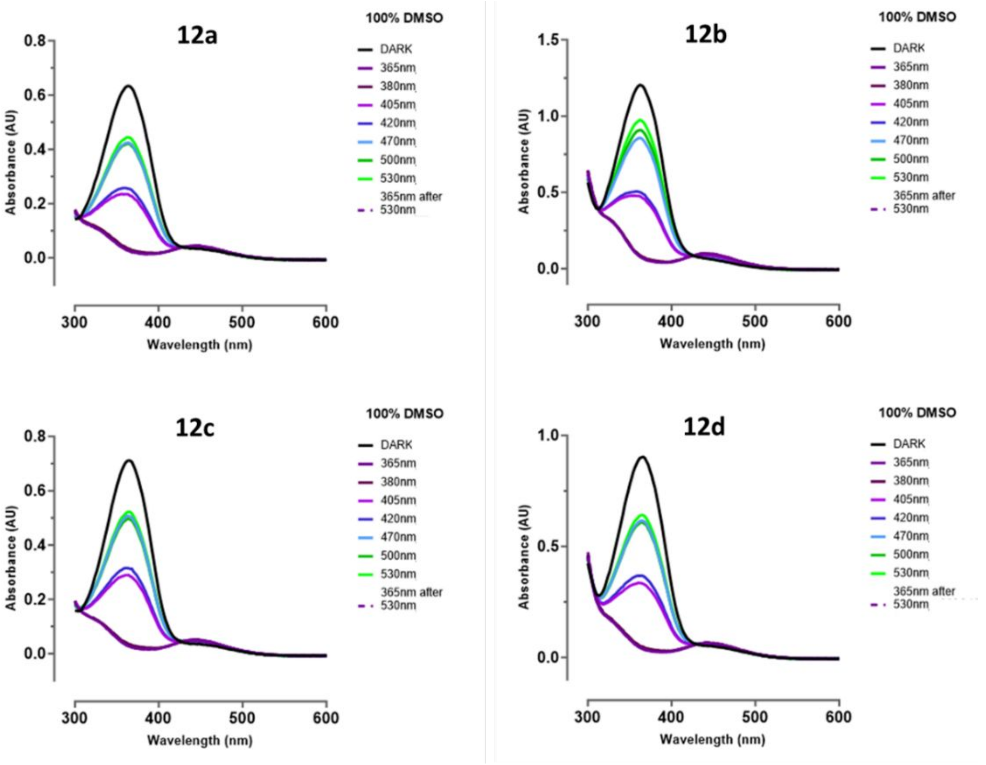

Figure S3. Photochemical properties of alcohol derivatives in DMSO. UV-Vis absorption spectra of compound 12a-d under different light conditions.

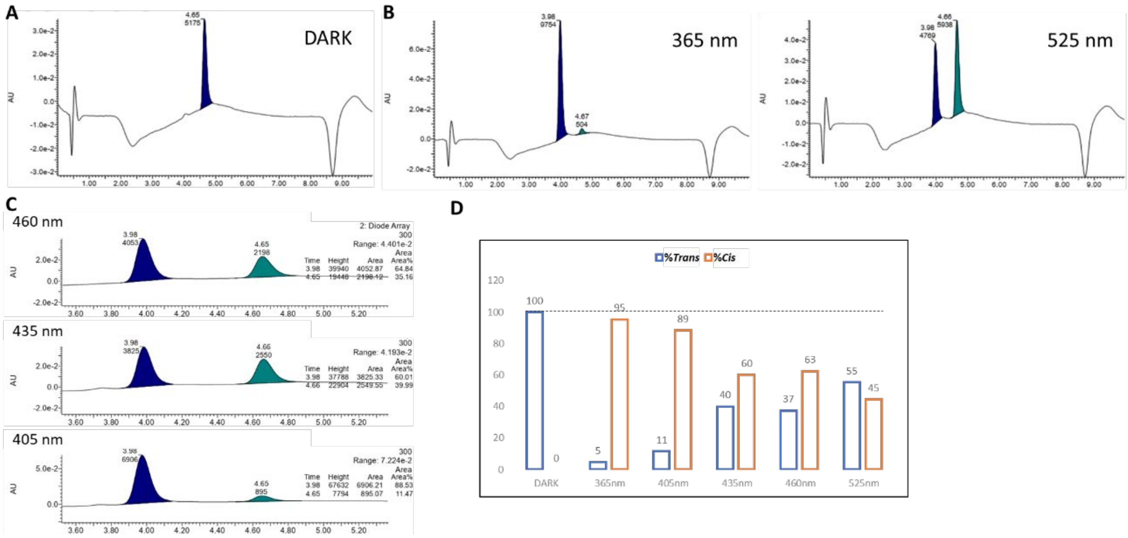

Figure S4. Photostationary state areas of 11b determined by HLPC in the assay buffer for pharmacological experiments (0,6% DMSO). (A) Measurement in the dark and after illumination with light at (B) 365 nm, 525 nm, and (C) 405 nm, 435 nm and 460nm. (D) Isomeric proportions of **11b** under the different light conditions.

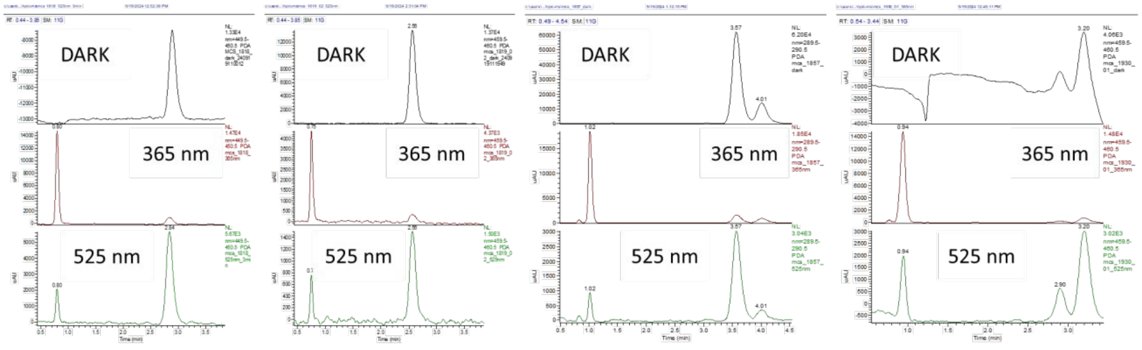

10a **10b** **10c** **10d**

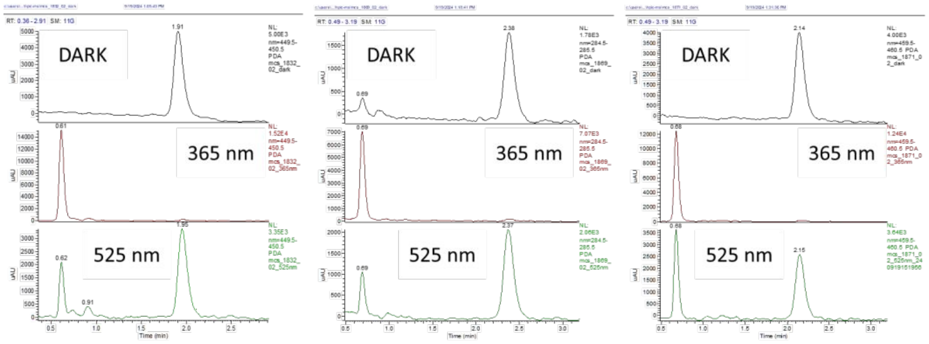

11a **11c** **11d**

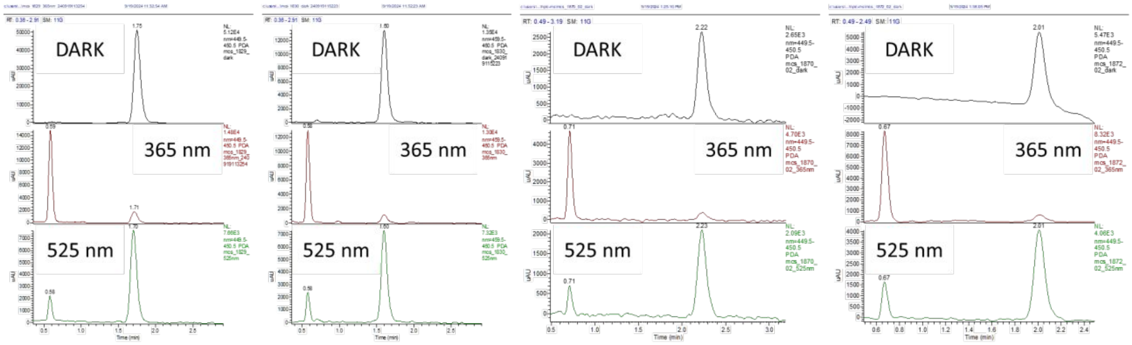

12a **12b** **12c** **12d**

Figure S5. Photostationary state areas of the compound series determined by HLPC in the dark and after illumination at 365 nm and 525nm in the assay buffer for pharmacological experiments (0,6% DMSO). Measurement in the dark and after illumination with light at 365 nm and 525 nm.

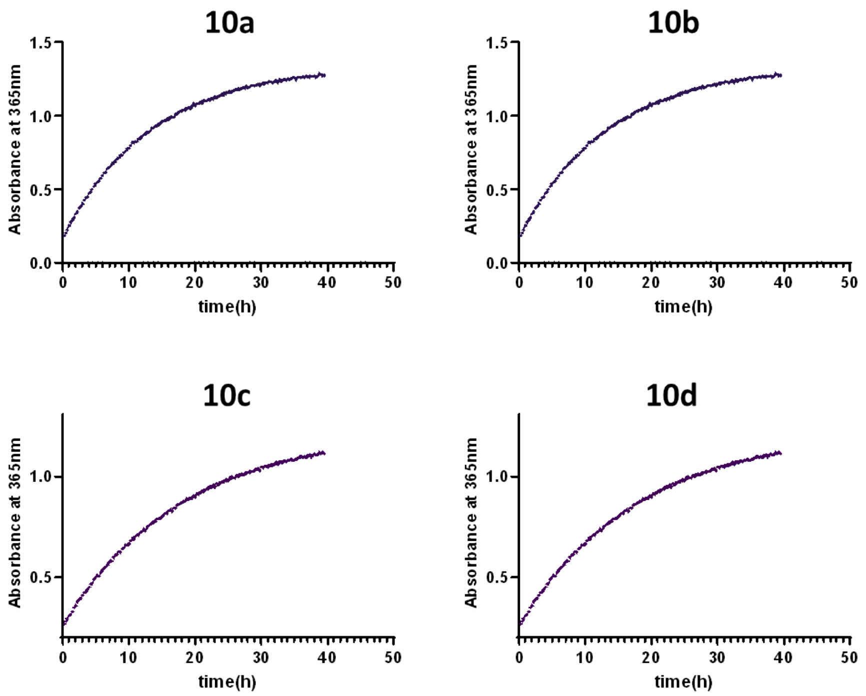

Figure S6. Thermal relaxation of ester derivatives in DMSO. Half-lifetime estimation of compounds 10a-d, absorbance was measured at 365 nm.

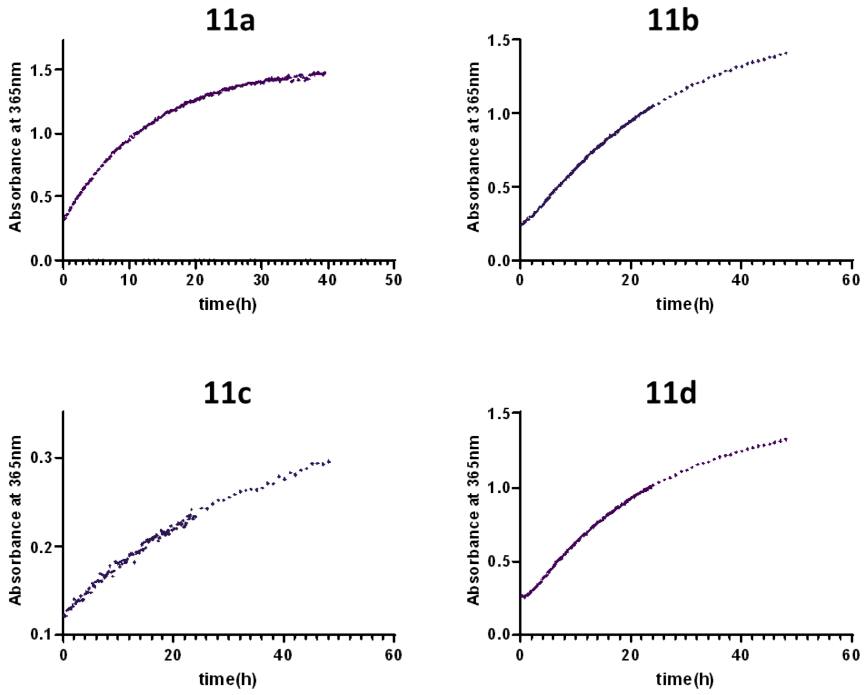

Figure S7 Thermal relaxation of carboxylic acid derivatives in DMSO. Half-lifetime estimation of compounds 11a-d, absorbance was measured at 365 nm.

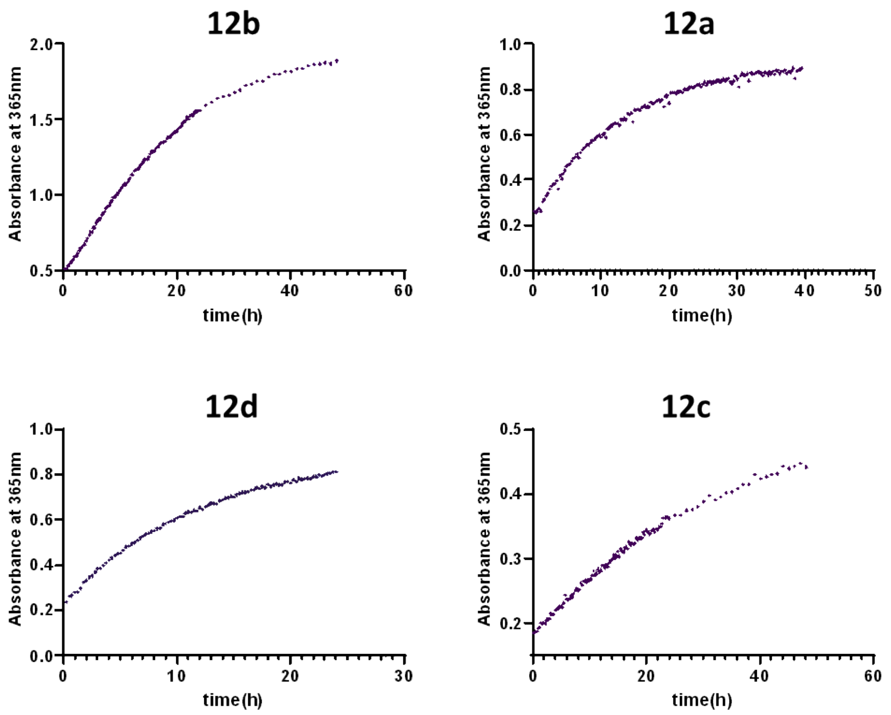

Figure S8. Thermal relaxation of alcohol derivatives in DMSO. Half-lifetime estimation of compounds 12a-d, absorbance was measured at 365 nm.

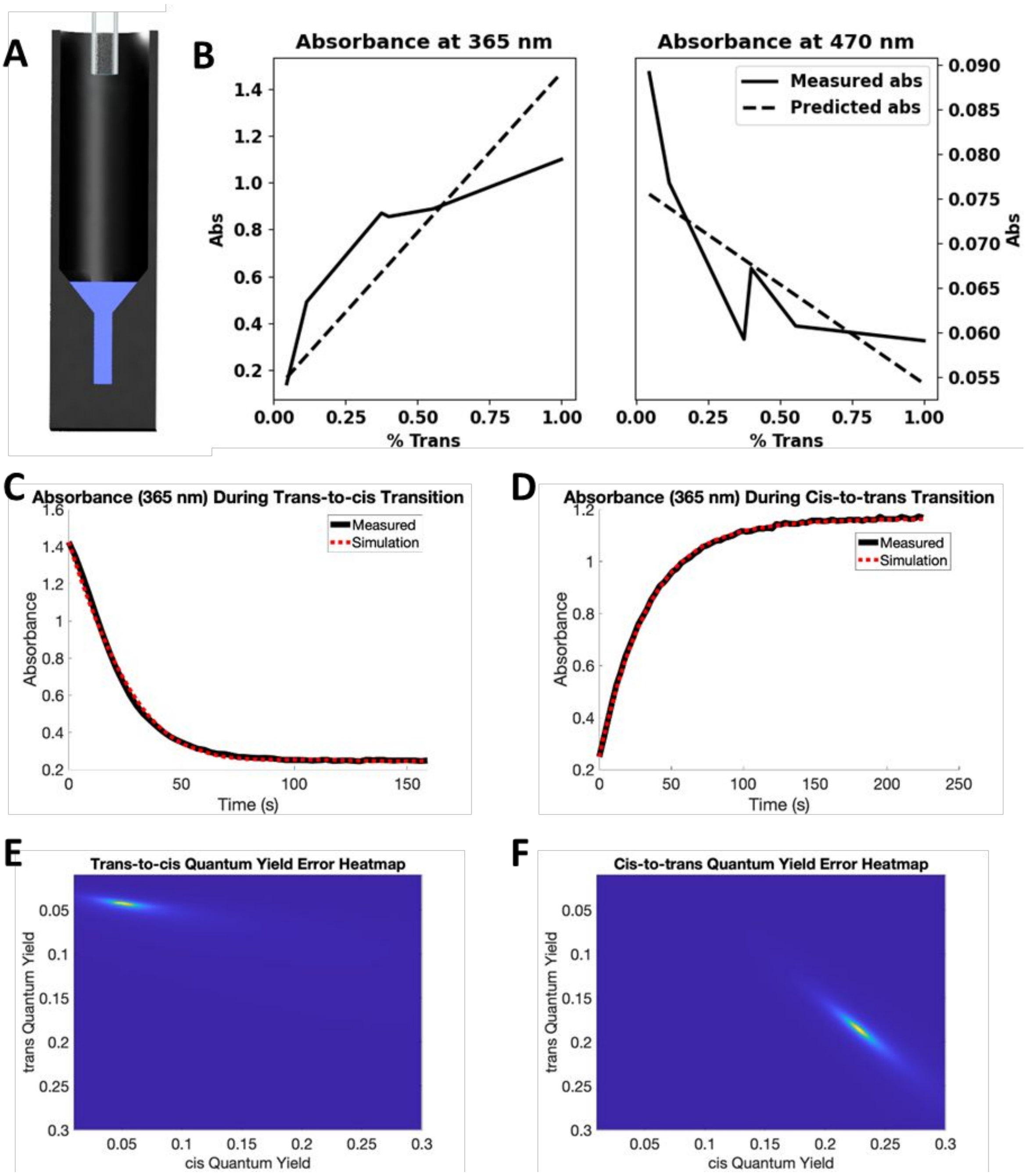

Figure S9. Photoisomerization Quantum Yield calculation. (A) 3D rendering of the cross section of the cuvette with the liquid light guide (translucent gray) and test solution (blue), made with Autodesk Fusion 360. (B) Measured absorbances of 50 µM compound **11b** (solid lines) were used to calculate *trans* and *cis* molar absorptivity coefficients at 365 nm and 470 nm: $\varepsilon_{trans,365}$ = 29395 $M^{-1}cm^{-1}$, $\varepsilon_{cis,365}$ = 2110 $M^{-1}cm^{-1}$, $\varepsilon_{trans,470}$ = 1086 $M^{-1}cm^{-1}$, $\varepsilon_{cis,470}$ = 1530 $M^{-1}cm^{-1}$. (C) and (D) show the real recorded absorbance measurements (taken at 365 nm) while 365 nm (E) or 470 nm (F) excitation light was output from the liquid waveguide. C and D qualitatively show the error maps (lighter colors show lower error) used to determine optimal quantum yield values.

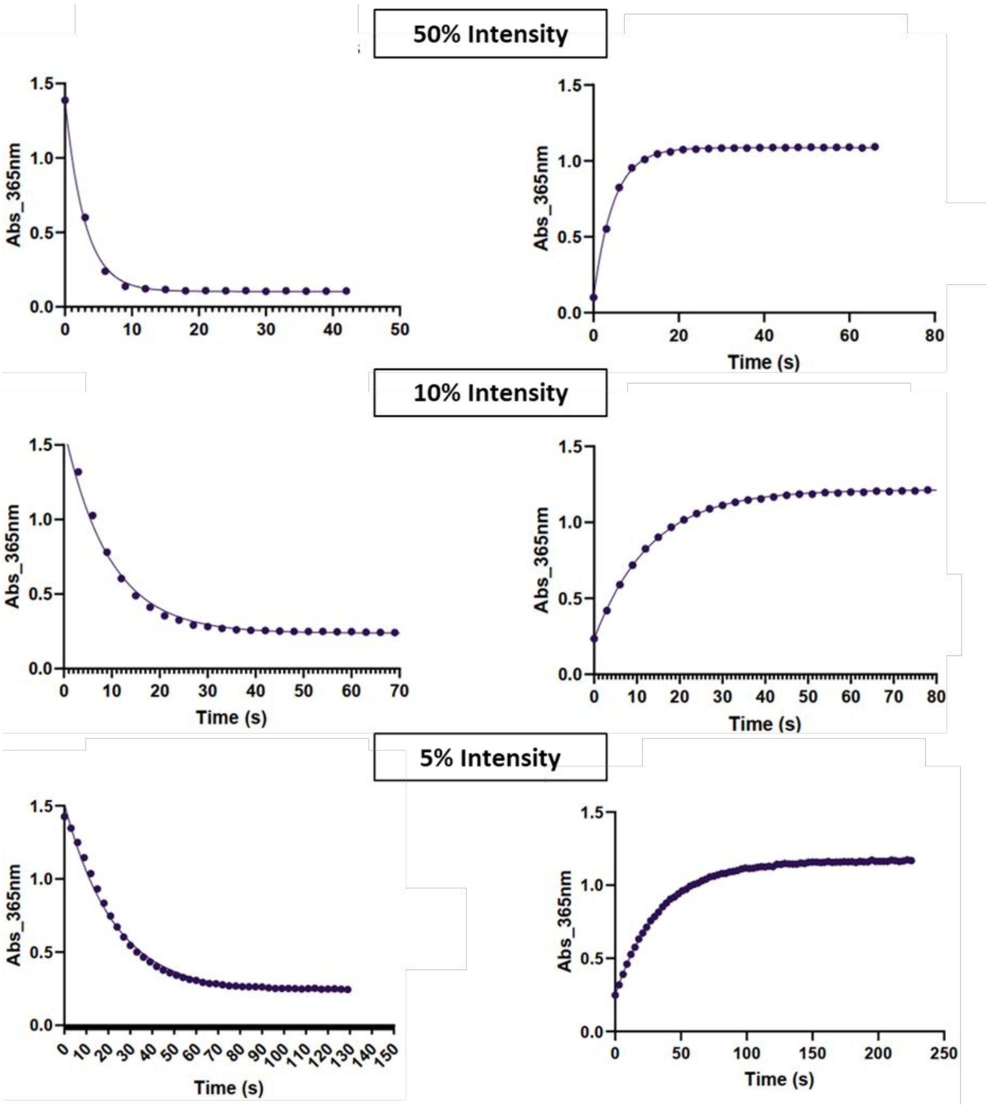

Figure S10. Photophysical properties of 11b under constant illumination. Photoisomerization under constant illumination with light at 365 nm (violet shade) and 470 nm (blue shade). Light was applied with a CoolLED pe-4000 at 50% intensity that corresponded to 0.75 mW/mm2 for the light at 365 nm and 0.56 mW/mm2 for the light at 470 nm; 10% intensity corresponded to 0.15 mW/mm2 for the light at 365 nm and 0.12 mW/mm2 for the light at 470 nm; 5% intensity corresponded to 0.06 mW/mm2 for the light at 365 nm and 0.05 mW/mm2 for the light at 470 nm.

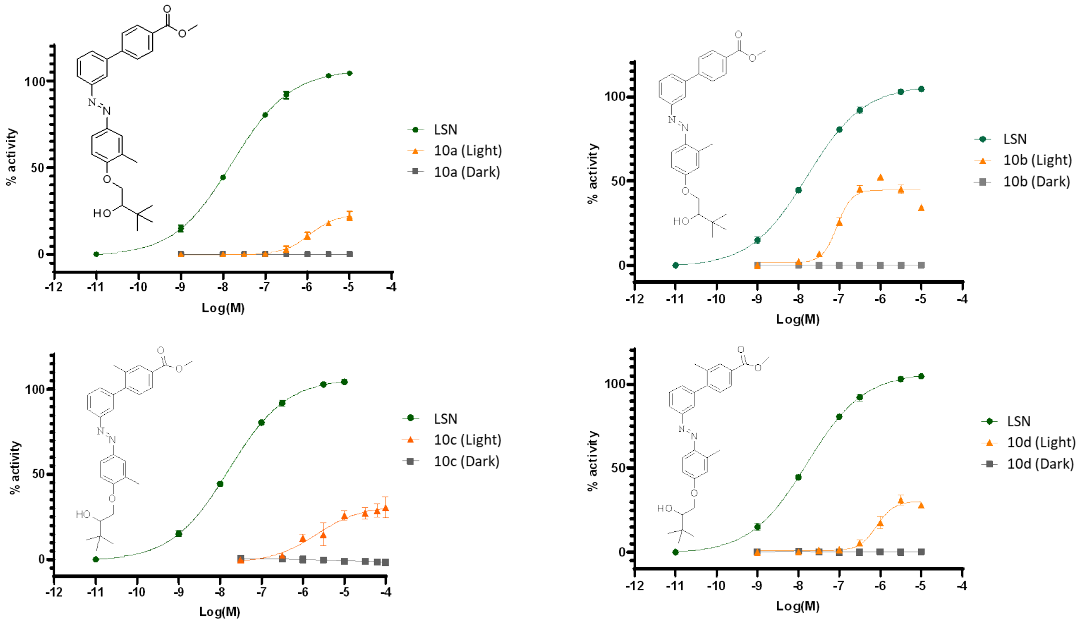

Figure S11. Concentration-response curves of ester derivatives. Dose-response curves of compounds 10a-d in the dark and under constant light (365 nm). The non-photoswitchable compound LSN2148936 was assayed as a reference.

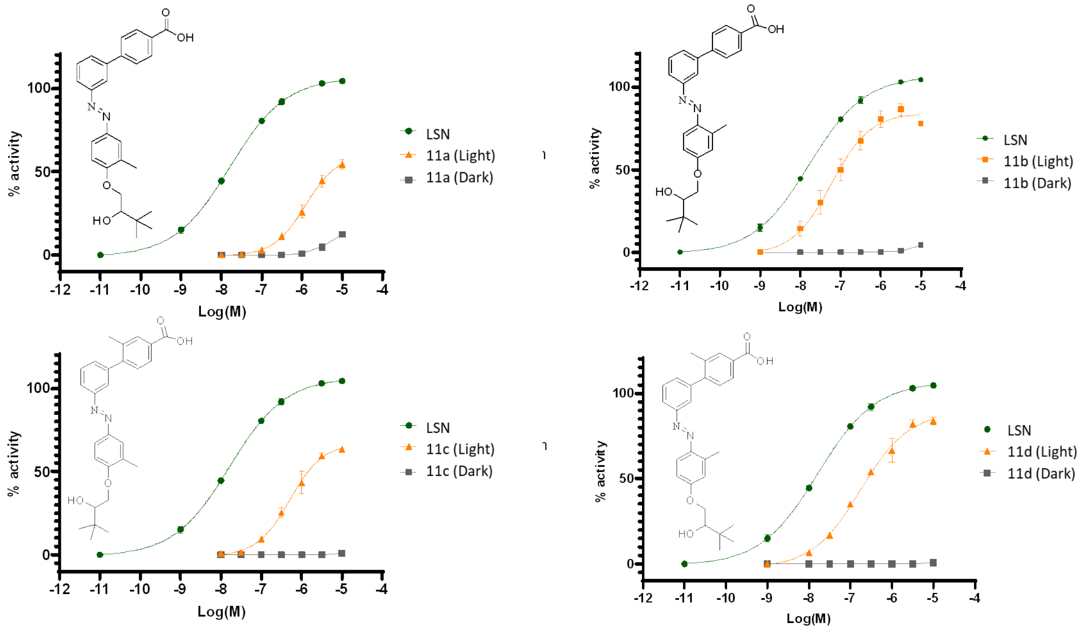

Figure S12. Concentration-response curves of carboxylic acid derivatives. Dose-response curves of compounds 11a-d in the dark and under constant light (365 nm). The non-photoswitchable compound LSN2148936 was assayed as a reference.

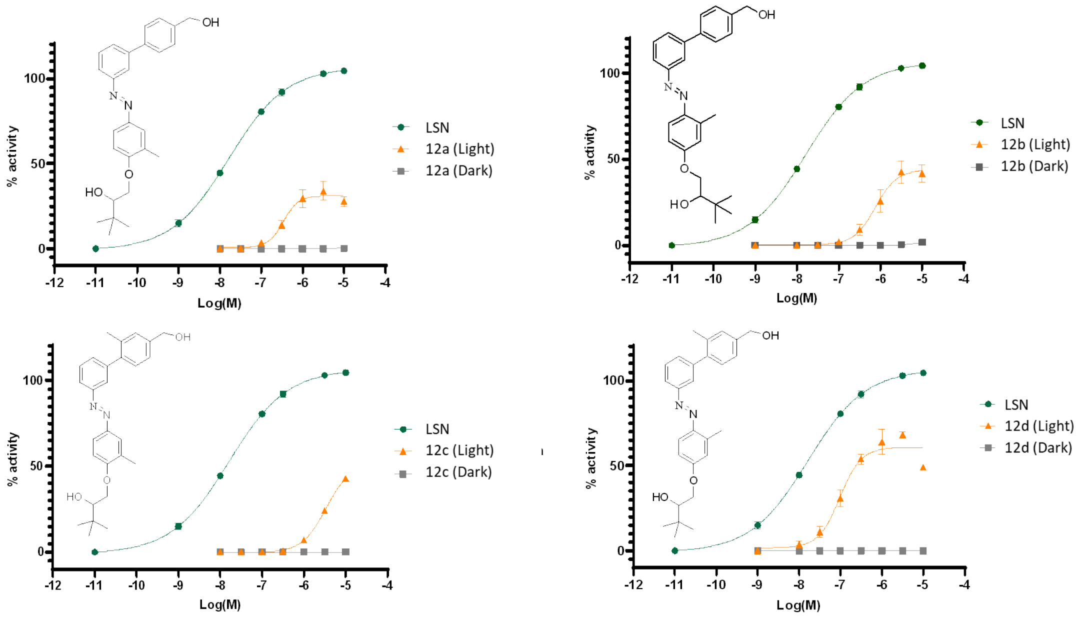

Figure S13. Concentration-response curves of alcohol derivatives. Dose-response curves of compounds 12a-d in the dark and under constant light (365 nm). The non-photoswitchable compound LSN2148936 was assayed as a reference.

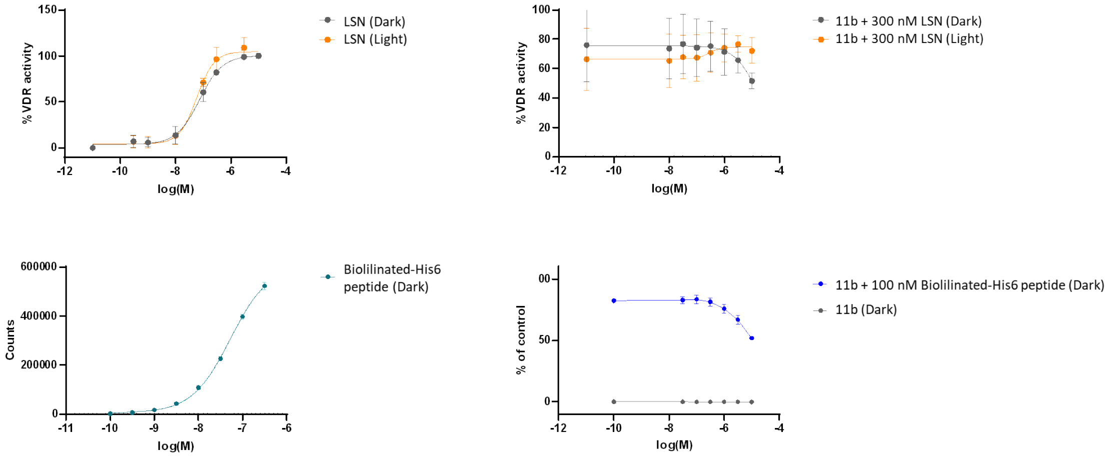

Figure S14. Pharmacological controls. Light does not affect LSN and 11b does not act as antagonist. Dose-response curves of compound LSN2148936 was assayed in the dark and under constant light at 365 nm(up-left panel). Several concentrations of compound **11b** with LSN2148936 at 300nM demonstrated that the compound does no act as antagonist in the dark or under illumination at 365 nm (up-right panel). Several concentrations of compound **11b** in the absence of VDR and RxR demonstrate that the increase of signal is due to their interaction (down-right panel). A slight decrease of the signal is observed for the highest concentrations.

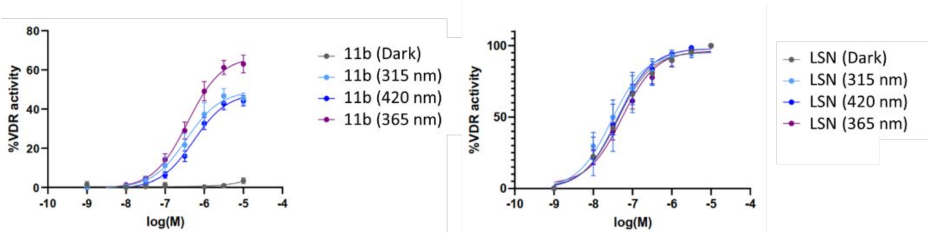

Figure S15. Pharmacological activity of 11b and LSN2148936 upon irradiation with different light wavelengths. Dose-response curves of compound **11b** and LSN2148936 in the dark and under constant light at 365 nm, 315 nm and 420 nm.

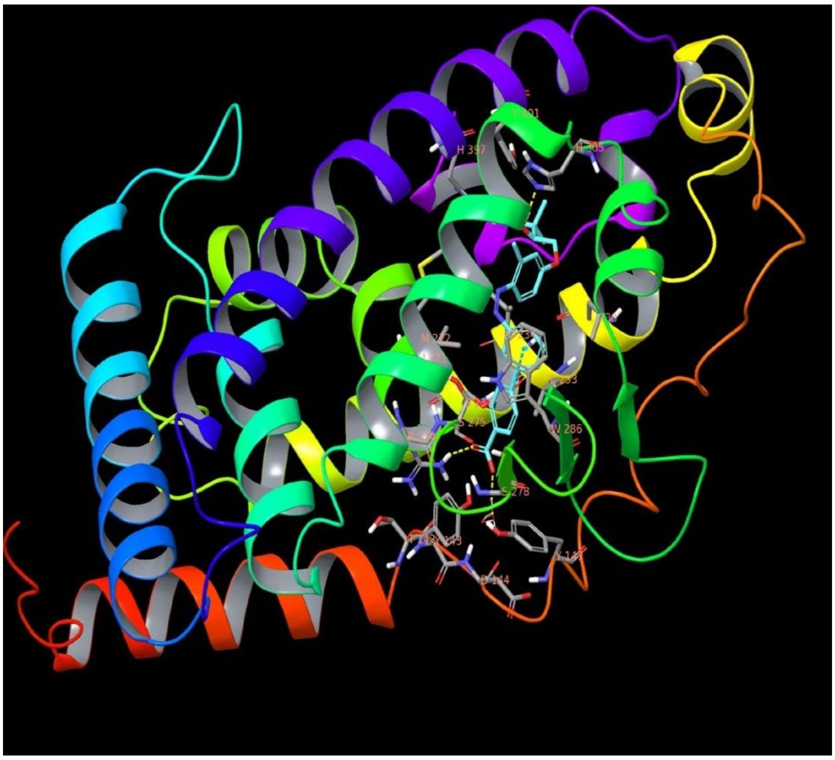

Ligand	MMGBSA pose	MD ligand interaction diagram
<u>(S)-*cis*-</u><br>**<u>11b</u>**	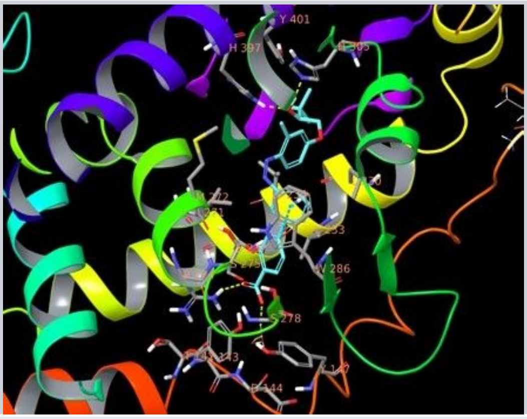	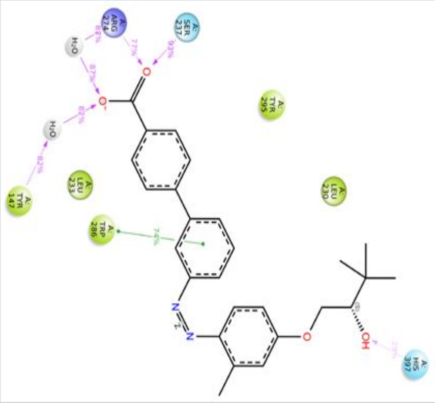
(R)-*cis*-<br>**11b**	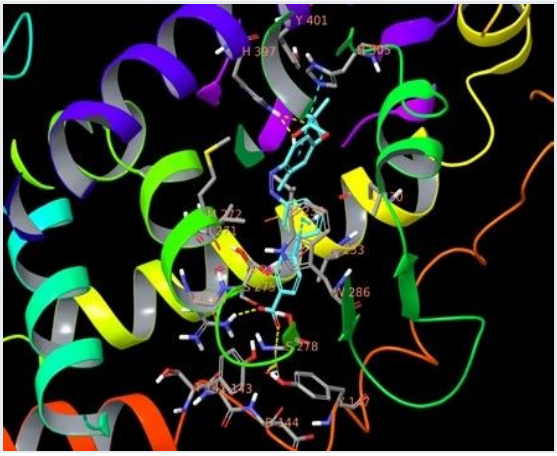	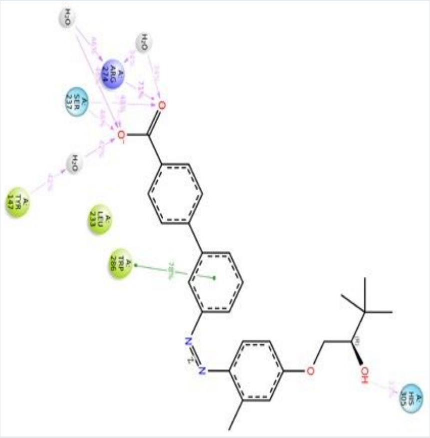

(S)*trans*-**11b**	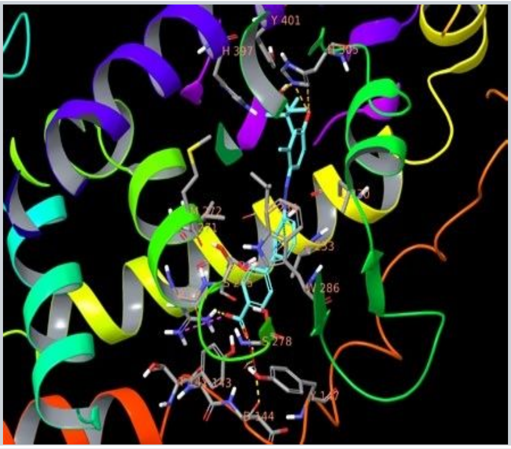	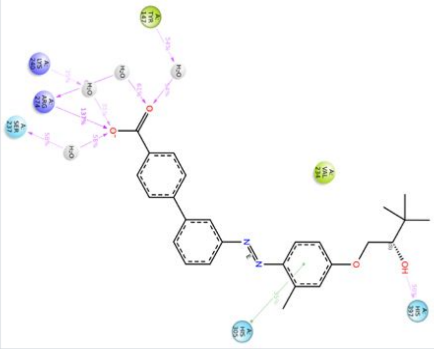
(R)*trans*-**11b**	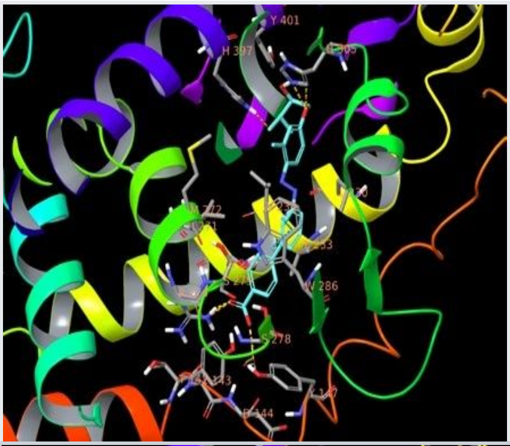	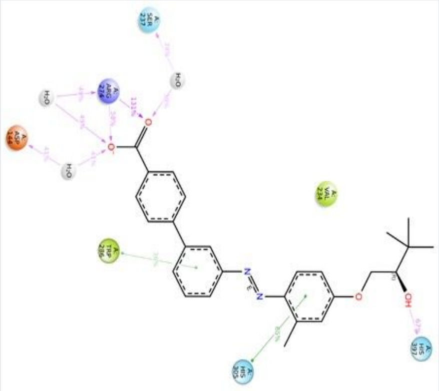
LSN	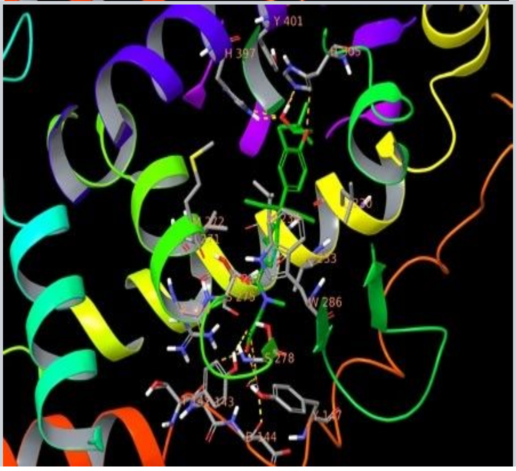	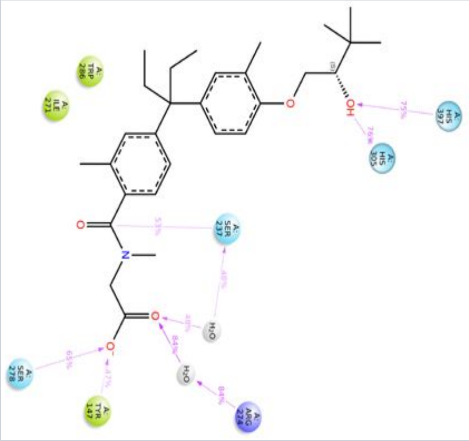

Figure S16. Optimal binding modes of various ligands to a model of VDR (derived from the structure with PDB code: 3B0T). (Top) General orientation of the following detailed figures illustrating (S)-cis-**11b.** (Table) Key interacting residues are shown, illustrating how *cis* **PhotoVDRM** (**11b**) and **LSN2148936** (**LSN**) occupy the binding pocket and interact with key residues while trans (S)-**PhotoVDRM** adopts a different binding mode, illustrated by the MMGBSA optimum poses of each ligand (second column) and the ligand interaction diagrams generated using 500ns of MD simulation (Desmond, standard SPC water box, 150mM NaCl). The stronger interaction of the *cis* form with Trp286 is clear. The R and S forms of each of the double-bond isomers show binding interactions similar to each other. However, the distinction in activity between any pair of *cis* and *trans* enantiomers is markedly pronounced (in particular, see the larger number of interactions with hydrophobic (green) residues in the ligand interaction diagrams vs. the *trans* form), consistent with the *cis*/*trans* isomerization being far more important than R/S.

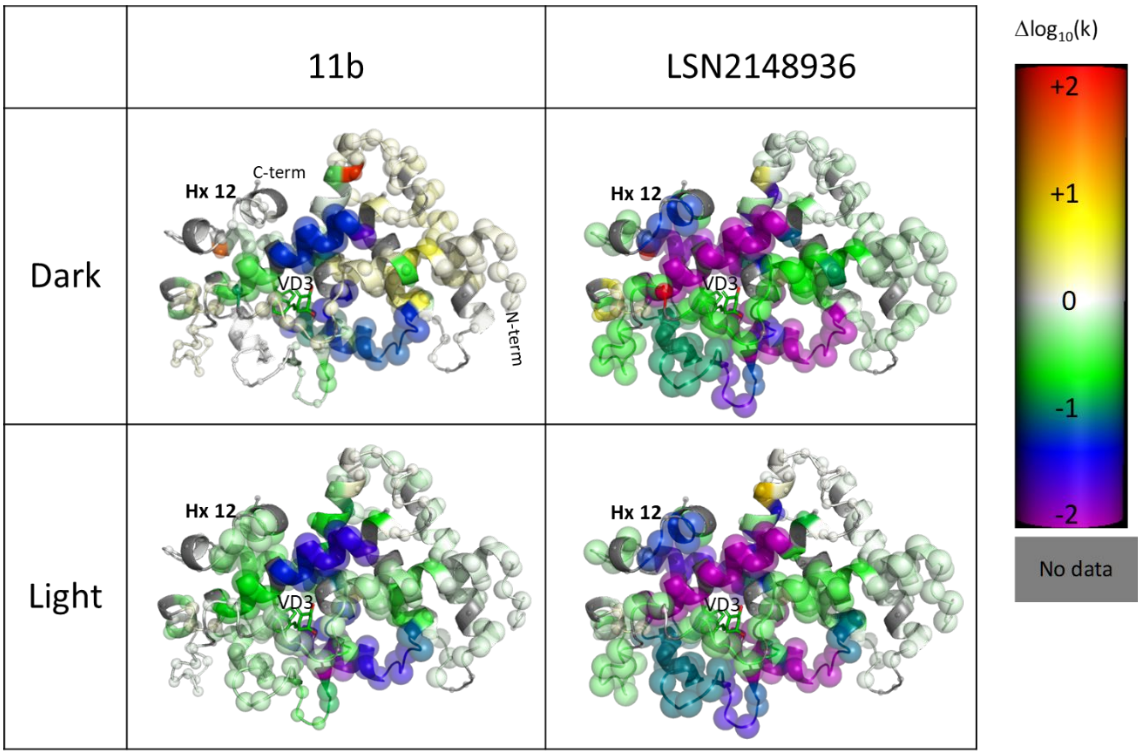

Figure S17. Differential deuterium uptake of VDR-LBD in the presence of ligand and different light conditions. Difference in log of rate constants (log10(k) - see color scale) for H/D exchange of VDR-LBD in the presence of racemic **11b** (left) or LSN2148936 (right), in the dark (top row), in the light (bottom row), vs. the equivalent apo experiment. Note the larger regions with green, blue and purple coloring in the bottom row for **11b** vs the top row, and the lack of any significant difference for LSN. The greater protection afforded by the photoisomerized *cis* **11b** vs the *trans*, particularly in Helix 12, whilst only small artefactual differences are seen for LSN. All panels show the VD3 molecule modeled on the basis of the ligand present in 3B0T purely as illustrative of the location of the canonical binding pocket. In each panel, the larger the spheres, the higher the confidence that the delta-log10(k) value is different from zero.

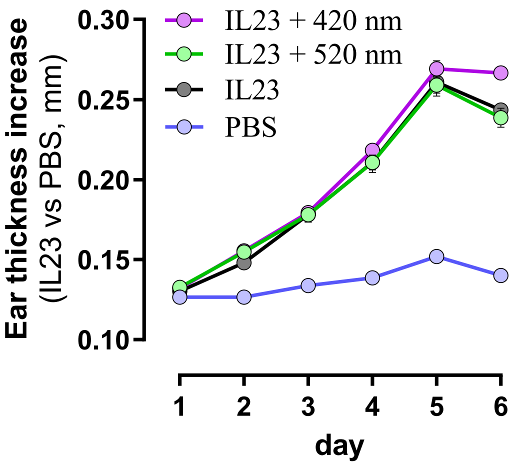

Figure S18. Controls of mouse psoriasis model. Mice ear thickness determination in animals administrated (i.p.) with vehicle in the dark and under 420 or 520 nm light. Each day, ear thickness

was measured in millimeters and represented as percentage of ear thickness increase using the first measurement of PBS-administrated ear as control.

REFERENCES

(1) Espada, A.; Haro, R.; Castañon, J.; Sayago, C.; Perez-Cozar, F.; Cano, L.; Redero, P.; Molina-Martin, M.; Broughton, H.; Stites, R. E.; Pascal, B. D.; Griffin, P. R.; Dodge, J. A.; Chalmers, M. J. A Decoupled Automation Platform for Hydrogen/Deuterium Exchange Mass Spectrometry Experiments. *J Am Soc Mass Spectrom* 2019, *30* (12), 2580–2583. https://doi.org/10.1007/S13361-019-02331-2.

(2) Stranius, K.; Börjesson, K. Determining the Photoisomerization Quantum Yield of Photoswitchable Molecules in Solution and in the Solid State. *Scientific Reports 2017 7:1* 2017, *7* (1), 1–9. https://doi.org/10.1038/srep41145.

(3) Swinehart, D. F. The Beer-Lambert Law. *J Chem Educ* 1962, *39* (7), 333–335. https://doi.org/10.1021/ED039P333.

(4) Friesner, R. A.; Murphy, R. B.; Repasky, M. P.; Frye, L. L.; Greenwood, J. R.; Halgren, T. A.; Sanschagrin, P. C.; Mainz, D. T. Extra Precision Glide: Docking and Scoring Incorporating a Model of Hydrophobic Enclosure for Protein-Ligand Complexes. *J Med Chem* 2006, *49* (21), 6177–6196. https://doi.org/10.1021/JM051256O.

(5) Friesner, R. A.; Banks, J. L.; Murphy, R. B.; Halgren, T. A.; Klicic, J. J.; Mainz, D. T.; Repasky, M. P.; Knoll, E. H.; Shelley, M.; Perry, J. K.; Shaw, D. E.; Francis, P.; Shenkin, P. S. Glide: A New Approach for Rapid, Accurate Docking and Scoring. 1. Method and Assessment of Docking Accuracy. *J Med Chem* 2004, *47* (7), 1739–1749. https://doi.org/10.1021/JM0306430.

(6) Halgren, T. A.; Murphy, R. B.; Friesner, R. A.; Beard, H. S.; Frye, L. L.; Pollard, W. T.; Banks, J. L. Glide: A New Approach for Rapid, Accurate Docking and Scoring. 2. Enrichment Factors in Database Screening. *J Med Chem* 2004, *47* (7), 1750–1759. https://doi.org/10.1021/JM030644S.

(7) Berman, H. M.; Westbrook, J.; Feng, Z.; Gilliland, G.; Bhat, T. N.; Weissig, H.; Shindyalov, I. N.; Bourne, P. E. The Protein Data Bank. *Nucleic Acids Res* 2000, *28* (1), 235–242. https://doi.org/10.1093/NAR/28.1.235.

(8) Li, J.; Abel, R.; Zhu, K.; Cao, Y.; Zhao, S.; Friesner, R. A. The VSGB 2.0 Model: A next Generation Energy Model for High Resolution Protein Structure Modeling. *Proteins* 2011, *79* (10), 2794–2812. https://doi.org/10.1002/PROT.23106.
