# Supplementary material for: Light-Regulated Agonists Spatiotemporally Activating the Vitamin D Receptor Mitigate Psoriasis-like Inflammation in Mice without Inducing Hypercalcemia
Source: ACS Cent Sci. 2025 Oct 21;11(12):2340–52. doi: 10.1021/acscentsci.5c00987 (PMC12746157; doi:10.1021/acscentsci.5c00987)
Supplement: Supplementary file 2 [file oc5c00987_si_002.pdf]

oc-2025-00987c.R1

Name: Peer Review Information for "Light-regulated agonists spatiotemporally activating the Vitamin D receptor mitigate psoriasis-like inflammation in mice without inducing hypercalcemia"

First Round of Reviewer Comments

Reviewer: 1

Comments to the Author

The present manuscript, submitted by Rovira, Venkatesh, Amadeu, and co-workers, reports a small series of photoswitchable compounds targeting the vitamin D receptor (VDR). While the strategy of modifying a receptor agonist with an azobenzene moiety to enable light-induced isomerization is not novel, I find this work potentially interesting for the readers of ACS Central Science.

On the one hand, the authors determine the binding mode of the hit compound 11b using HDX-MS, a state-of-the-art technique for probing protein conformational changes and ligand-binding sites. On the other hand, they provide evidence that the compound reduces inflammation in a mouse model without affecting plasma calcium levels.

Overall, I believe the manuscript is of sufficient quality to warrant publication. However, I do have a few minor comments for the authors.

1. Modeling section. The authors present simple molecular docking for the binding of the photoswitchable molecule and the parental agonist. In addition to that, they ran some binding energies using the MMGBSA method. I consider this model quite simplistic because the orientation of residue W286 with respect to the bound ligand can vary if the authors consider the dynamics of the receptor in the presence of the different ligands. Indeed, in Figure 3 I cannot agree with 'only the cis photoisomer of both enantiomers

positions the central ring planar and at a suitable distance from W286 to form attractive staggered stacking interactions'. The author revisit and clarify which are clearly the differences for the key interactions of the cis- and trans-isomers of 11b.

2. Scheme 1. The authors should ensure that all building blocks are consistently oriented throughout the chemical synthesis, avoiding any rotations or flips of structures. For example, compounds 9 and 10 appear misaligned, and the position of the NO moiety in compound 7 differs from its orientation in compound 10.

Reviewer: 2

Comments to the Author

Rovira *et al*. describe an impressive and comprehensive discovery story of photoswitchable VDR agonists. Relying on a purely chemical concept, the best compound was taken all the way to *in vivo* studies, where it performed well without a known calcium-related side e ect. The di erence between *trans* and *cis* states is remarkable and of note in the photopharmacology field.

The breadth of techniques used is impressive, and include photochemical and photophysical characterization of racemate and enantiomers, QM calculations, molecular modelling and protein H/D exchange experiments. The data accompanying the paper in the main text and SI is highly appropriate. Proper controls are taken along all into *in vivo*, i.e., LSN2148936 which was also exposed to illumination (which should not give a di erence for this non-photoswitchable compound).

I therefore think this article is of interest to the broader readership of ACS Cent Sci. I would recommend publication with minor revisions. I list my suggestions below:

1) Page 6 - Design: The design section was a bit unclear to me. The authors could be more specific in their design strategy to enhance the clarity. I would advise to insert a Figure graphically showing the design strategy and also including the structure of calcitriol. Additionally, I was wondering why esters, alcohols and acids are all used in parallel, with the latter of course being deprotonated. Moreover, what was the rationale behind the methyl substitutions and why only at selected aromatic positions. Lastly, it would help to already mention the residues in the VDR pocket that are crucial for target

interactions, especially because the authors later proceed to look at the protein in more detail with their modelling studies.

2) Page 6/7 - Synthesis: The synthesis section is arranged in a slightly odd fashion. I would recommend starting with the order as also dictated by molecule numbers. Perhaps the authors could consider moving Scheme S1 and S2 from the SI to the manuscript and combine it with Scheme 1. Also, I would suggest that the authors add yields to the scheme.

3) Page 8 – Table 1: To me, it is not clear why PSS values were determined at 12 degrees (legend Table 1) and what bu er the authors used for their photochemical characterization. Likewise, no specific bu er was mentioned on Page 9 – line 24, and Page 10 – line 38.

4) Page 11 - Table 2: With the structure showing the trans configuration but the data being for the PSS_{cis}, this table may lead to confusion. Although most of the trans compounds are inactive in this assay (Fig S11-13), it would help the reader to at least give the lowest concentration at which the trans compounds were tested, e.g. by adding a column ”Activity trans” with values listed being ‘<5.00’ for easy visual comparison for the reader. Additionally, I would advise to make it clear in the current headers of the table that that data shown is the Activity at PSS_{365}. I would also recommend adding to the legend of this table if the SD/SEM is reported and the number of repetitions performed, as in Figure 2.

5) Page 12 – Figure 2B: Figure 2B to me has little purpose, as after initial screening all compounds were taken to full dose-response curves anyway (Table 2 and Figures S11-S13). I would recommend removing Fig 2B, as it is slightly distracting now.

6) Page 13/14 - Modelling: A concern I have involves the docking of (S)-11b. The authors propose π-π stacking of the ligand with W286 as the key di erence in binding mode between trans and cis. However, this stacking interaction seems to be absent in the pose for control LSN2148931. Also, it feels to me that this π-π interaction alone cannot solely explain the huge di erence in pEC_{50} (7.23) for the PSS_{365} versus that of the trans (pEC_{50} < 5.0 judging from Fig. S11). Could the di erence in activity that the authors report, perhaps also come from a shape mismatch for the trans, given that the co-crystallized ligand (maxacalcitol) in the utilized PDB is bent? More generally, it seems there may be an inherent risk of overinterpretation if one tries to model a virtually inactive compound such as the trans? Lastly, I would suggest adjusting Figure 3, by showing clearly the essential interactions (including hydrogen bonding interaction) and an appropriate color scheme as the color of the ligand and protein are quite similar, making it harder to see.

7) Page 14 - HDX: Given the broad readership, I would suggest the authors to introduce the reader a bit more to the concept of HDX-MS as this may be a technique not everyone is familiar with. A bit more explanation on the unfamiliar terms RxR and TRAP220 could be provided.
8) Page 16-17 – in vivo: Could the authors comment on the decision to use the racemate in vivo? Of course, for in vitro assays using the racemate is less of a problem, since the authors show that both enantiomers both have similar in vitro activity. But in vivo other biochemical events are perhaps not so stereochemically indi erent and this can potentially a ect the results.
9) Page 16-17 – in vivo: The lack of calcium side-e ects (Fig. 5D) is very impressive. Do the authors have a hypothesis about why their compound does not show this sidee ect, while LSN2148936 does show this side-e ect?
10) Page 16-17 – in vivo: The statement that 11b is "unable to preclude the IL-23-induced psoriatic-like phenotype" in vivo for PSS_{525} does not seem optimal, as Figure 5C shows some residual e ects (green curve), which in turn is to be expected as Table 1 lists a PSS_{525} value of 55 for 11b, meaning 45% active cis is still present. I would advise to rephrase this to something like "only partially reduces the IL-23-inuduced psoriatic-like phenotype"
11) Wavelenghts: Throughout the manuscript, di erent wavelengths are used for key compound 11b. However, it is not always completely clear why a specific wavelength is chosen in the di erent situations. The authors use 365 nm for in vitro work, but use 420 nm in their in vivo work. While there are likely valid reasons for this and while in vitro PSS and in vivo PSS percentages will likely not correlate quantitatively, PSS values at 420 nm are not reported in the manuscript. If I look at Figure 1A and Figure S4D and use some eyeballing/extrapolation, the di erences in PSS values at 420 and 525 nm (both wavelengths used for in vivo) do not seem very large. I would advise the authors to justify their choice of wavelengths for in vivo (include a referral to Figure S4D) and explain why the pure trans isomer was excluded from in vivo studies. It will be helpful to add the PSS values to Fig S4D.

Typos/graphical adjustments:

1) General: Text can be polished here and there in terms of grammar. I advise to avoid colloquial language such as "game-changing".
2) Page 5 – line 22/23: IL-2 is listed twice

3) Page 7 - Scheme 1: The bond angles of compound 9a/b, the ester 10a-d and carboxylic acid 11a-c need some addressing. Also, the same holds true for the X groups on Page 11 – Table 2. The bond angle of the ethereal oxygen atom can be improved in Scheme 1, Table 2 and Figure 2.
4) Page 8 – Table 1 & Page 9 – line 4: Given that the PSS for 11d was determined by HPLC, it would be more appropriate to report it as '>99%' rather than '100%'.
5) Page 9 – line 10: The text refers to the "thermal half-life at room temperature" while the table legend showed that the thermal half-life was measured at 37 degrees.
6) Page 12- line 13 & 14: pEC50 needs subscript
7) Page 12 – Figure 2: resolution of chemical structures is suboptimal and needs addressing
8) Page 13 – line 3: Figure 15S -> Figure S15
9) Page 13 – line 21: 1.30 A ->1.30 Å
10) SI: several figures, such as Figure S16A, appear to have suboptimal resolution.
11) SI: chemical analyses should be included for key compound 11b (i.e., HPLC chromatograms and NMR spectra)

But again, I want to compliment the authors for a very nice and comprehensive story that shows that photopharmacology has matured from a curiosity-driven area to a field in which it starts to provide real potential benefits, especially in skin-related diseases where light penetration is not as much an issue compared to e.g. CNS diseases.

Reviewer: 3

Comments to the Author

The manuscript entitled"Light-regulated agonists spatiotemporally activating the Vitamin D receptor mitigate psoriasis-like inflammation in mice without inducing hypercalcemia" by Rovira et al. presents the design, synthesis, and biological evaluation of a library of azobenzene-based ligands capable of light-controlled activation of the vitamin D receptor (VDR). The authors identify compound 11b as the most promising candidate based on its favourable photochemical properties (cis % 100 but trans % 61) and its biological activity, with a pEC_{50} comparable to that of the reference non-secosteroidal VDR agonist LSN21148936 (AKA VDRM2) according to luminescent proximity homogeneous assays. In

addition, molecular modeling and HDX-MS studies were employed to investigate the binding mode, isomer effect and conformational changes associated with VDR activation. Finally, the in vivo efficacy of 11b is evaluated in a mouse model of psoriasis, where localized light exposure triggers a therapeutic anti-inflammatory effect without inducing systemic hypercalcemia, unlike the control LSN21148936. In my opinion, the manuscript has potential for ACS Central Science due to its originality, multidisciplinary nature and impact of the topic. To my knowledge, light-controllable VDR agonists have not been reported yet, and they have translational impact, especially for dermatological indications such as psoriasis.

I have some points that require clarification, and I encourage the authors to address them. In addition, I would like to suggest a few experiments and revisions that could further strengthen the manuscript.

1) The rationale behind the design of the ligand library is difficult to follow, especially for readers who are not experts in VDR. In the introduction, the authors limit the precedents to calcipotriol as a VDR agonist, but do not explain more structurally similar ligands, such as LSN21148936 (VDRM2), which is later used as a control. It would be helpful to better contextualise the design strategy using the key scaffolds and introduce a new figure (the reference to Fig 3, as in the original manuscript do not really fit) so that the synthetic rationale and chemical evolution of the molecules (e.g., functional groups retained or altered, introduction of the photoswitch). Along the same lines, the selection of 11b as a candidate (Fig. 1) comes too early and breaks the flow of the story. I recommend having first the activity experiments to justify the highlight of the characterisation of 11b.

2) Due to the simplicity of the photoswitchable molecules, it would be terrific if the authors could address the selectivity, at least of the best candidate and compare it with the reference. These experiments would also help to dispel any doubts about the non-expected similarities between enantiomers. I do not quite understand how the authors justify these results therefore some extra clarification would be highly desirable. Both molecular dynamics calculations and HDX must be completed with the enantiomers.

3) In vitro activity assays: Azobenzene is a quenchers; please verify that this is not creating any artefact in your assay. Please, include data without normalisation. It is hard to understand how some of the pEC50 values could be calculated with such accuracy when, in many cases, the plateau is not reached. Please revisit table 2.

4) The HDX difference between 11b and LSN21148936 is significant and needs extra clarification. What are the used concentrations?

5) To avoid misleading conclusions, the results should be complete, including the trans irradiation, given the concerning 60% of the isomer. Also, if only one cycle can be performed, the general text should tone down.

6) It looks to me that there are inconsistencies in the general tendency of the activity compounds according to Table 2 and Figure 2b; for instance, I would expect a higher value for 12b (there are more). How did the authors explain these discrepancies? Is the chosen concentration the most appropriate? Please also include the time of irradiation, which is also an important factor for toxicity (please revisit the statement: "used for phototherapy and non-phototoxic visible illumination at 420 nm modulated the activity of compound 11b"

7) Figure 5: B readers who are not experts with these experiments would need some extra explanation of those figures; you may consider using arrows, etc., to guide the interpretation. C significant differences (*) should be explained as in D; Are the ** at 520 nm correct?

Minor comments:

a) Table 1 -> What is the composition of "buffer"? Why 12 degrees of temperature? Which light source?

b) I would tone down "we developed innovative experimental methods based on HDX-MS". Implementation of the in situ irradiation should be incorporated in the supporting information.

c) Why do not all compounds have the HRMS? It would be helpful to know which compounds are new and which ones have been described.

d) Figure S5, which is the identity of the extra peaks that appear in 11a, 10c, 10d?

e) Although a reference to the quantum yield determination was included, the readers would appreciate a detailed protocol in the supporting information of this manuscript. Also include the calculation of all the compounds to verify consistency.

f) The calculation of lifetimes is unclear; please clarify it.

g) Table 2, please include the irradiation time

h) Figure S14 "biolilinated-His6" has not been appropriately introduce in the main manuscript

i) Figure 2E, please specify buffer composition

j) Figure S11 twice 10b compound

Reviewer: 4

Comments to the Author

Rovira, Krishnan, Llebaria et al. report the design, synthesis, and biological validation of light-regulated vitamin D receptor (VDR) agonists for the treatment of psoriasis-like inflammation. The researchers incorporated a photoswitchable azobenzene moiety into VDR ligands, thus converting them into photohormones that allow for precise spatiotemporal control of drug activation using (365/525 nm) light cycles.

The lead compound, 11b, is only minimally active in the dark (trans form) but is converted to an active agonist (cis form) upon illumination with UV or visible blue light, wavelengths already used in dermatology. Spectroscopic studies confirmed robust photoisomerization, with thermal half-lives exceeding 6 hours in the cis state, allowing sustained activity after activation. The isomerization kinetics as a function of light intensity are informative and useful. However, the photostationary state (PSS) of 11b at 525 nm shows only 55% trans (in solution), raising the question of why the remaining 45% cis does not result in substantial receptor activation. This point should be discussed in more depth. What was the PPS at 460 nm and why was this wavelength not chosen?

Innovative hydrogen/deuterium exchange mass spectrometry (HDX-MS) combined with molecular modeling revealed that the cis form of 11b engages critical VDR residues (e.g., W286) essential for receptor activation, whereas the trans form does not. This explains the light-dependent pharmacological switch. It would be interesting to know whether this represents a case of an “efficacy switch.” Perhaps the authors could address this in the discussion.

In vivo, systemic administration of 11b in mice followed by localized light irradiation on psoriatic skin lesions significantly reduced inflammation without causing systemic hypercalcemia, a common and dangerous side effect of traditional VDR agonists. This

demonstrates that light can be used to selectively activate the drug at diseased sites, offering a major safety advantage.

Prior art has been appropriately cited. However, the sentence “Photopharmacological agents have been proposed for several nuclear receptors” should be corrected to read: “Photopharmacological agents have been demonstrated for several nuclear receptors,” and the references reordered to highlight the pioneering work of Trauner and Merk starting in 2019 (cf. references 12 and 13).

Overall, this work establishes the first photopharmacological approach to light-controlled activation of the vitamin D receptor in vivo, opening avenues for safer, localized treatments of skin diseases like psoriasis. The approach may be relevant to other nuclear receptor targets and diseases where topical or spatially restricted therapy is advantageous and constitutes and important advance int the development of Photopharmacology.

Author's Response to Peer Review Comments:

See the attached file.

We have uploaded a new version for the manuscript and the supplememtary information, as well as a point by point response to reviewers and the two files tracking the changes in the new version compared to the first orginal submission (for both manuscript and supporting information).

Amadeu Llebaria Soldevila Barcelona, August, 29th 2025

Prof. Dr. Leticia Gonzalez
Senior Editor
ACS Central Science
gonzalez-office@centralscience.acs.org

Journal: ACS Central Science

Manuscript ID: oc-2025-00987c

Original Submission Date: 30-May-2025

Title: "Light-regulated agonists spatiotemporally activating the Vitamin D receptor mitigate psoriasis-like inflammation in mice without inducing hypercalcemia"

Author(s): Rovira, xavier; Espada, Alfonso; Serra, Carme; Catena, Juanlo Lorenzo; Lopez-Cano, Marc; Panarello, Silvia; Pérez-Albaladejo, Elisabet; Broughton, Howard; Cano, Leticia; Aijeren, Hans; Khan, Sadid; Álvarez-Montoya, Paula; Muñoz, Lourdes; Font, Joan; Trapero, Ana; Rivero,
Pablo; Li, Yangrong; Ma, Donghui; Lin, Yinxiang; Ma, Linda; Dodge, Jeffrey; Dai, Mingji; Irazoqui,
Pedro; Ciruela, Francisco; Krishnan, Venkatesh; Llebaria, Amadeu

----

Please, note that answers and comments are highlighted in blue

RESPONSE TO THE EDITORIAL OFFICE

Formatting Needs:

Author List: Please include the email address(es) of the corresponding author(s) on the first page of the manuscript.

Supporting Information: If the manuscript is accompanied by any Supporting Information for Publication, a brief description of the supplementary material is required in the manuscript, before the reference list. The appropriate format is: Supporting Information. Brief statement in non-sentence format listing the contents of the material supplied as Supporting Information. Please list each supporting item individually.

*Examples of sufficient descriptions: “Supporting Information: 1H NMR spectra for all compounds” or “Additional experimental details, materials, and methods, including photographs of experimental setup.”

*Examples of insufficient descriptions: “Supporting Information: Figures S1-S3” or “Additional figures as mentioned in the text.”

A brief description of the supporting information has been added to the manuscript.

Supporting Information: Please number all pages in the following format: S1, S2, S3, etc.

All pages of the Supporting Information have been numbered using the specified format.

Synopsis: ACS Central Science requires a brief synopsis. The synopsis should be no more than 200 characters (including spaces) and should reasonably correlate with the Table of Contents (TOC) graphic. The synopsis is intended to explain the importance of the article to a broader readership across the sciences. Please place your synopsis in the manuscript file after the TOC graphic and label as “Synopsis.”

TOC Graphic: Include a TOC graphic illustrating the significance of the paper. The TOC graphic should be something that is representative of your entire work. Color schemes or illustrations typically make good choices. The TOC graphic must be original and free from any copyright issues. Confirm that all text is legible. Present the TOC graphic on the last page of the manuscript by itself. Please label the TOC as “TOC Graphic”. A caption describing the TOC is not needed.

Please see more information/guidelines for TOC Graphics at the following link:
http://pubsapp.acs.org/paragonplus/submission/toc_abstract_graphics_guidelines.pdf?

Synopsis and TOC Graphic have been included at the end of the revised manuscript.

Additional note to the editor:

To facilitate the identification of the best-performing compound in future studies, we have replaced its original numbering (11b) with the name “photoVDRM”.

A new author, Dr Pablo Rivero, has been included after the revisions done, which required several original experimental contributions from him that deserve authorship. All the other authors have been informed accordingly and accepted this addition.

POINT BY POINT RESPONSE TO THE REFEREES

Reviewer: 1

Recommendation: Publish in ACS Central Science after minor revisions noted.

Comments:
The present manuscript, submitted by Rovira, Venkatesh, Amadeu, and co-workers, reports a small series of photoswitchable compounds targeting the vitamin D receptor (VDR). While the strategy of modifying a receptor agonist with an azobenzene moiety to enable light-induced isomerization is not novel, I find this work potentially interesting for the readers of ACS Central Science. On the one hand, the authors determine the binding mode of the hit compound 11b using HDX-MS, a state-of-the-art technique for probing protein conformational changes and ligand-binding sites. On the other hand, they provide evidence that the compound reduces inflammation in a mouse model without affecting plasma calcium levels. Overall, I believe the manuscript is of sufficient quality to warrant publication. However, I do have a few minor comments for the authors.

We thank the reviewer for his/her positive comments.

1. Modeling section. The authors present simple molecular docking for the binding of the photoswitchable molecule and the parental agonist. In addition to that, they ran some binding energies using the MMGBSA method. I consider this model quite simplistic because the orientation of residue W286 with respect to the bound ligand can vary if the authors consider the dynamics of the receptor in the presence of the different ligands. Indeed, in Figure 3 I cannot agree with 'only the cis photoisomer of both enantiomers positions the central ring planar and at a suitable distance from W286 to form attractive staggered stacking interactions'. The author revisit and clarify which are clearly the differences for the key interactions of the cis- and transisomers of 11b.

We have changed the language in the main text to reflect more closely the common feature of interaction with W286, which is that the region around that residue and, particularly, the indole ring is packed by hydrophobic groups (the perhydroindane in calcitriol, the alkyl chains in our reference molecule, and the aromatic ring we have commented upon in 11b). We have made it clearer that we are contrasting the cis and trans 11b isomers, rather than making a general comment on stacking as being a driving force for receptor binding and/or activation. We have also added material to the S16 figure in the supporting information to show the frequent interactions observed between the cis and trans forms of R and S 11b during unbiased molecular dynamics simulations starting from the docked poses, and we have improved the resolution of the images as far as possible.

2. Scheme 1. The authors should ensure that all building blocks are consistently oriented throughout the chemical synthesis, avoiding any rotations or flips of structures. For example, compounds 9 and 10 appear misaligned, and the position of the NO moiety in compound 7 differs from its orientation in compound 10.

Scheme 1 has been modified as recommended by the reviewer.

Additional Questions:

Quality of experimental data, technical rigor: Top 10%

Significance to chemistry researchers in this and related fields: Top 10%

Broad interest to other researchers: Top 10%

Novelty: Top 10%

Is this research study suitable for media coverage or a First Reactions (a News & Views piece in the journal)?: Yes

Reviewer: 2

Recommendation: Publish in ACS Central Science after minor revisions noted.

Comments:

Rovira et al. describe an impressive and comprehensive discovery story of photoswitchable VDR agonists. Relying on a purely chemical concept, the best compound was taken all the way to in vivo studies, where it performed well without a known calcium-related side effect.

The difference between trans and cis states is remarkable and of note in the photopharmacology field. The breadth of techniques used is impressive, and include photochemical and photophysical characterization of racemate and enantiomers, QM calculations, molecular modelling and protein H/D exchange experiments. The data accompanying the paper in the main text and SI is highly appropriate. Proper controls are taken along all into in vivo, i.e., LSN2148936 which was also exposed to illumination (which should not give a difference for this nonphotoswitchable compound).

I therefore think this article is of interest to the broader readership of ACS Cent Sci. I would recommend publication with minor revisions.

We thank the reviewer for his/her positive comments.

I list my suggestions below:

1) Page 6 - Design: The design section was a bit unclear to me. The authors could be more specific in their design strategy to enhance the clarity. I would advise to insert a Figure graphically showing the design strategy and also including the structure of calcitriol. Additionally, I was wondering why esters, alcohols and acids are all used in parallel, with the latter of course being deprotonated. Moreover, what was the rationale behind the methyl substitutions and why only at selected aromatic positions.

We agree with the reviewer that a clearer explanation of the rationale behind the molecular design helps clarify the strategy for developing photoswitchable VDR agonists. Therefore, we have added both an explanation and an additional figure to the revised manuscript as suggested.

Lastly, it would help to already mention the residues in the VDR pocket that are crucial for target interactions, especially because the authors later proceed to look at the protein in more detail with their modelling studies.

Later in the article there is a description of the most important amino acid residues responsible for the interaction of calcitriol together with a reference in this respect: “However, only the cis isomer accomplishes the well-established interactions of the natural hormone calcitriol, which engages the amino acids W286, L233, R274, H397 and Y401” and “Rochel, N.; Wurtz, J. M.; Mitschler, A.; Klaholz, B.; Moras, D. The Crystal Structure of the Nuclear Receptor for Vitamin D Bound to Its Natural Ligand. Mol Cell 2000, 5 (1), 173–179.”.

Additionally, a new figure of the interaction of the ligand with those important residues and an extra explanation has been introduced upon a suggestion of another review.

2) Page 6/7 - Synthesis: The synthesis section is arranged in a slightly odd fashion. I would recommend starting with the order as also dictated by molecule numbers. Perhaps the authors could consider moving Scheme S1 and S2 from the SI to the manuscript and combine it with Scheme 1.

Following reviewer’s suggestion, a new Scheme 1, moving Scheme S1 and S2 from the SI to the manuscript and being ordered by molecule numbers has been included.

Also, I would suggest that the authors add yields to the scheme.

We believe that, since these are general schemes in which the same reaction yields up to four different compounds, the resulting yields are different. In any case, the yield for each compound can be found in the Supporting Information.

3) Page 8 – Table 1: To me, it is not clear why PSS values were determined at 12 degrees (legend Table 1) and what buffer the authors used for their photochemical characterization. Likewise, no specific buffer was mentioned on Page 9 – line 24, and Page 10 – line 38.

We thank the reviewer for pointing this out. The value was indeed incorrect and has been corrected in the revised version of the manuscript (“12°C” ❼ “21°C”).

The buffer used is specified in the supporting information (methods section) but we agree with the reviewer that this is an important condition that should be mentioned in the table legend and other sections of the manuscript. Therefore, we have modified the text accordingly.

4) Page 11 - Table 2: With the structure showing the trans configuration but the data being for the PSScis, this table may lead to confusion. Although most of the trans compounds are inactive in this assay (Fig S11-13), it would help the reader to at least give the lowest concentration at which the trans compounds were tested, e.g. by adding a column "Activity trans" with values listed being '<5.00' for easy visual comparison for the reader. Additionally, I would advise to make it clear in the current headers of the table that that data shown is the Activity at PSS365. I would also recommend adding to the legend of this table if the SD/SEM is reported and the number of repetitions performed, as in Figure 2.

We thank the reviewer for this relevant comment. We have added a note on the table caption to clarify the conditions and statistics applied to perform the pharmacological experiments for clarity.

5) Page 12 – Figure 2B: Figure 2B to me has little purpose, as after initial screening all compounds were taken to full dose-response curves anyway (Table 2 and Figures S11-S13). I would recommend removing Fig 2B, as it is slightly distracting now.

We believe this panel is informative, as it illustrates the procedure followed to identify the most active compound and highlights the range of activities observed across the entire set of developed compounds. It also demonstrates the rich structure–activity relationship (SAR) revealed through slight structural modifications, which may inspire future optimization efforts. Therefore, following an internal discussion prompted by the comment of the reviewer, the authors have decided to retain this panel in the manuscript.

6) Page 13/14 - Modelling: A concern I have involves the docking of (S)-11b. The authors propose π-π stacking of the ligand with W286 as the key difference in binding mode between trans and cis. However, this stacking interaction seems to be absent in the pose for control LSN2148931. Also, it feels to me that this π-π interaction alone cannot solely explain the huge difference in pEC50 (7.23) for the PSS365 versus that of the trans (pEC50 < 5.0 judging from Fig. S11). Could the difference in activity that the authors report, perhaps also come from a shape mismatch for the trans, given that the co-crystallized ligand (maxacalcitol) in the utilized PDB is bent? More generally, it seems there may be an inherent risk of overinterpretation if one tries to model a virtually inactive compound such as the trans? Lastly, I would suggest adjusting Figure 3, by showing clearly the essential interactions (including hydrogen bonding interaction) and an appropriate color scheme as the color of the ligand and protein are quite similar, making it harder to see.

We have explained the large pEC_{50} difference as being likely to be due to changes in both affinity and efficacy; we agree that the difference in binding expected from the predicted bound conformation would be unlikely to provide such a large difference on its own. We have also adjusted the text to more clearly reflect our view that the important interactions are hydrophobic, and that these include a stronger pi-stacked interaction for the cis than for the trans or the reference molecule. We have added some diagrams based upon molecular dynamics which show the most frequent interactions observed between ligand and protein which support and to some extent quantify these observations.

7) Page 14 - HDX: Given the broad readership, I would suggest the authors to introduce the reader a bit more to the concept of HDX-MS as this may be a technique not everyone is familiar with. A bit more explanation on the unfamiliar terms RxR and TRAP220 could be provided.

We thank the reviewer for this relevant recommendation. To help readers to clearly understand, the sentence "Strikingly, HDX-MS experiments revealed changes in protection that were significantly stronger in the experiment with light, and also showed some interesting qualitative differences vs. the dark state (Figure 4 and S17)" was changed to "Strikingly, HDX-MS experiments revealed changes in protection to exchange backbone NH protons (typically observed when ligand interaction modulate the conformational state and dynamic behavior of the protein) that were significantly stronger in the experiment with light, and also showed some interesting qualitative differences vs. the dark state (Figure 4 and S17)". Additionally, recent literature, including a review, dealing with the application of HDX to drug discovery has been included as a reference (54 to 56). On the other hand, in the introduction (page 4) we describe RxR (retinoid-X receptor), a partner of the VDR protein and on page 8, TRAP220 is defined as a fragment of the transcriptional coactivator, known in the literature as the TRAP220 subunit.

8) Page 16-17 – in vivo: Could the authors comment on the decision to use the racemate in vivo? Of course, for in vitro assays using the racemate is less of a problem, since the authors show that both enantiomers both have similar in vitro activity. But in vivo other biochemical events are perhaps not so stereochemically indifferent and this can potentially affect the results.

This is a relevant comment from the reviewer, since in medicinal chemistry it is known that different isomers may lead to different biological activities. The exploration of this possibility is guaranteed in future studies and we have added a sentence in the discussion section on this regard. However, this research would require to synthesize enantiomerically pure ligands and, therefore, the investigation of new synthetic routes suitable for to this purpose. Moreover, testing the different compounds in vivo would highly increase the amount of work and the number of animals, that would also require additional proper controls. Indeed, the antipsoriatic effect found may come from the any of the two isomers of both. However, this is highly specific of the VDR activity and, therefore, the authors think that this question is beyond the scope of the current research, which aims to demonstrate the possibility to develop photoswitchable VDR agonists with in vivo activity devoid of systemic side effects.

9) Page 16-17 – in vivo: The lack of calcium side-effects (Fig. 5D) is very impressive. Do the authors have a hypothesis about why their compound does not show this side effect, while LSN2148936 does show this side-effect?

We were also surprised by these results, as we expected a reduction but not a complete elimination of calcemic effects. Our hypothesis is that the concentration of compound locally activated is not very high but enough to reduce inflammation. It is known that VDR activation derives in a gain-of-function long lasting effects, which may explain why only a mild, short and punctual action over this receptor may be sufficient for these impressive results. Then, subsequent dilution of the activated drug reduces its concentration sufficiently to prevent systemic effects. Further studies with this compound are planned to try to shed light on this phenomenon, which may help to define the underlying mechanism and, we hope, new therapeutic interventions.

10) Page 16-17 – in vivo: The statement that 11b is "unable to preclude the IL-23-induced psoriatic-like phenotype" in vivo for PSS525 does not seem optimal, as Figure 5C shows some residual effects (green curve), which in turn is to be expected as Table 1 lists a PSS525 value of 55 for 11b, meaning 45% active

cis is still present. I would advise to rephrase this to something like "only partially reduces the IL-23-inuduced psoriatic-like phenotype"

We have modified the sentence as suggested by the reviewer.

11) Wavelenghts: Throughout the manuscript, different wavelengths are used for key compound 11b. However, it is not always completely clear why a specific wavelength is chosen in the different situations. The authors use 365 nm for in vitro work, but use 420 nm in their in vivo work. While there are likely valid reasons for this and while in vitro PSS and in vivo PSS percentages will likely not correlate quantitatively, PSS values at 420 nm are not reported in the manuscript. If I look at Figure 1A and Figure S4D and use some eyeballing/extrapolation, the differences in PSS values at 420 and 525 nm (both wavelengths used for in vivo) do not seem very large. I would advise the authors to justify their choice of wavelengths for in vivo (include a referral to Figure S4D) and explain why the pure trans isomer was excluded from in vivo studies. It will be helpful to add the PSS values to Fig S4D.

We agree with the reviewer and, therefore, an additional table has been included in the supporting information providing the proportion of *cis* and *trans* for each molecule as calculated using the absorbance spectrums in Figures S1 to S3 in DMSO and the method described by Ernst Fischer (1967). We acknowledge that this is just an approximation of the proportion that will be found in vivo but we think it is informative since a reversal in the proportions of ligand 11b is found for the critical wavelengths pointed by the reviewer. Additionally, a sentence was been added to justify the use of 420 nm as a photoactivation wavelength: "In this model, we used the visible 420 nm light to induce photoconversion since we demonstrated that this wavelength could produce up to 60% PhotoVDRM cis activating VDR (Table S6 and Figure S15) and slightly improves tissue penetrability". Finally, the pure trans isomer was excluded from the study because the animals used for the experiment have to be maintained for their wellbeing in light/dark conditions established by Institutional Animal Care and Use Committee. In these conditions, the ambient light may activate the compound and alter the results. Therefore, the research group performing the experiments leaded by Prof. Ciruela, with an ample track record on *in vivo* photopharmacology, recommended performing the study using two external lights that would help control the illumination conditions and avoid alterations and misinterpretations. With all these limitations in mind, further studies are guaranteed upon a better control of the animal conditions in future research. However, a modification of the protocols and provably the animal maintenance room will be required along with the committee approval, which will take time since these are basic rules and instances for the animal experimentation of our institutions.

Typos/graphical adjustments:

1) General: Text can be polished here and there in terms of grammar. I advise to avoid colloquial language such as "game-changing".

The text has been reviewed by a native English speaker, and colloquial language has been removed or revised.

2) Page 5 – line 22/23: IL-2 is listed twice

This mistake has been corrected.

3) Page 7 - Scheme 1: The bond angles of compound 9a/b, the ester 10a-d and carboxylic acid 11a-c need some addressing.

Scheme 1 has been modified as recommended by the reviewer.

Also, the same holds true for the X groups on Page 11 – Table 2. The bond angle of the ethereal oxygen atom can be improved in Scheme 1, Table 2 and Figure 2.

Table 2 has been modified as suggested by the reviewer.

4) Page 8 – Table 1 & Page 9 – line 4: Given that the PSS for 11d was determined by HPLC, it would be more appropriate to report it as '>99%' rather than '100%'.

We agree with the reviewer and we have modified the table and text accordingly.

5) Page 9 – line 10: The text refers to the "thermal half-life at room temperature" while the table legend showed that the thermal half-life was measured at 37 degrees.

This mistake has been corrected. We thank the reviewer for pointing it out.

6) Page 12- line 13 & 14: pEC50 needs subscript

This mistake has been corrected. We thank the reviewer for pointing it out.

7) Page 12 – Figure 2: resolution of chemical structures is suboptimal and needs addressing

Figure 2 has been modified as was suggested by the reviewer.

8) Page 13 – line 3: Figure 15S -> Figure S15

This mistake has been corrected. We thank the reviewer for pointing it out.

9) Page 13 – line 21: 1.30 A ->1.30 Å

This mistake has been corrected. We thank the reviewer for pointing it out.

10) SI: several figures, such as Figure S16A, appear to have suboptimal resolution.

Figure S16 has been modified, and the resolution of the images has been enhanced.

11) SI: chemical analyses should be included for key compound 11b (i.e., 12 chromatograms and NMR spectra)

NMR spectra (1H and 13C) for compound 11b have been included in the SI

But again, I want to compliment the authors for a very nice and comprehensive story that shows that photopharmacology has matured from a curiosity-driven area to a field in which it starts to provide real potential benefits, especially in skin-related diseases where light penetration is not as much an issue compared to e.g. CNS diseases

We sincerely thank the reviewer for the thorough and useful revision, as well as this very positive and encouraging consideration.

Additional Questions:

Quality of experimental data, technical rigor: Top 10%

Significance to chemistry researchers in this and related fields: Top 10%

Broad interest to other researchers: Top 10%

Novelty: Top 10%

Is this research study suitable for media coverage or a First Reactions (a News & Views piece in the journal)?: Yes

Reviewer: 3

Recommendation: Major revisions required.

Comments:
The manuscript entitled "Light-regulated agonists spatiotemporally activating the Vitamin D receptor mitigate psoriasis-like inflammation in mice without inducing hypercalcemia" by Rovira et al. presents the design, synthesis, and biological evaluation of a library of azobenzene-based ligands capable of light-controlled activation of the vitamin D receptor (VDR). The authors identify compound 11b as the most promising candidate based on its favourable photochemical properties (cis % 100 but trans % 61) and its biological activity, with a pEC_{50} comparable to that of the reference non-secosteroidal VDR agonist LSN21148936 (AKA VDRM2) according to luminescent proximity homogeneous assays. In addition, molecular modeling and HDX-MS studies were employed to investigate the binding mode, isomer effect and conformational changes associated with VDR activation. Finally, the in vivo efficacy of 11b is evaluated in a mouse model of psoriasis, where localized light exposure triggers a therapeutic antiinflammatory effect without inducing systemic hypercalcemia, unlike the control LSN21148936. In my opinion, the manuscript has potential for ACS Central Science due to its originality, multidisciplinary nature and impact of the topic. To my knowledge, light-controllable VDR agonists have not been reported yet, and they have translational impact, especially for dermatological indications such as psoriasis. I have some points that require clarification, and I encourage the authors to address them. In addition, I would like to suggest a few experiments and revisions that could further strengthen the manuscript.

We thank the reviewer for his/her positive comments.

1) The rationale behind the design of the ligand library is difficult to follow, especially for readers who are not experts in VDR. In the introduction, the authors limit the precedents to calcipotriol as a VDR agonist, but do not explain more structurally similar ligands, such as LSN21148936 (VDRM2), which is later used as a control. It would be helpful to better contextualise the design strategy using the key scaffolds and introduce a new figure (the reference to Fig 3, as in the original manuscript do not really fit) so that the synthetic rationale and chemical evolution of the molecules (e.g., functional groups retained or altered, introduction of the photoswitch).

We thank the reviewer for this useful suggestion. We have added new text and a figure to explain the rational followed for the design of molecules. We believe this will now be much clearer for the reader.

Along the same lines, the selection of 11b as a candidate (Fig. 1) comes too early and breaks the flow of the story. I recommend having first the activity experiments to justify the highlight of the characterisation of 11b.

We appreciate the comment of the reviewer. However, following an internal discussion, we believe that the evaluation of the photochemical properties of the molecules should be presented first. While it is true that the detailed characterization of the entire compound library, provided in the Supporting Information, may not be evident to the reader, given that Figure 1 focuses on the main candidate, this order reflects the logic of our research. The photochemical characterization is a necessary step prior to conducting in vitro pharmacological assays under optimal light conditions. Therefore, we have decided to retain the current order of the figures and hope that this is acceptable to the reviewer.

2) Due to the simplicity of the photoswitchable molecules, it would be terrific if the authors could address the selectivity, at least of the best candidate and compare it with the reference. These experiments would also help to dispel any doubts about the non-expected similarities between enantiomers. I do not quite understand how the authors justify these results therefore some extra clarification would be highly desirable. Both molecular dynamics calculations and HDX must be completed with the enantiomers.

Regarding similarities between enantiomers, the lack of an effect here can be justified by an enhanced Fig 3 and Fig S16 with more detailed information on hydrogen bonding around those centers. We have also added a diagram based upon the frequency of observed interactions during MD simulations of each cis and trans forms of the R and S enantiomers of 11b which shows the enhanced hydrophobic interactions for the cis form and suggests qualitatively that the interactions with the R and S enantiomers may reasonably be expected to be similar.

We agree with the reviewer that studying the selectivity of compounds is a very interesting and relevant question. However, we believe that such a complex study goes beyond the scope of the current work. The results obtained from specific in vitro assays using purified proteins, along with the observed in vivo anti-inflammatory effects, closely resembling those of the reference compound LSN2148936, to our opinion provide sufficient evidence of the activity of compound 11b on VDR and its potential as an antipsoriatic agent. Nevertheless, comprehensive future studies with this compound or other related azobenzenes are warranted to thoroughly investigate the molecular mechanisms underlying receptor activation upon binding of the different drugs presented in this work. To state this clearly in the article a sentence has been added in the "Results and Discussion" section: "LSN2148936 is highly selective for VDR and the pharmacological responses are attributed to the activation of VDR and not off-target effects of other steroid receptors, such as the glucocorticoid. Future experiments using enantiomerically pure photoVDRM will be conducted to determine whether each isomer exhibits specific effects."

3) In vitro activity assays: Azobenzene is a quenchers; please verify that this is not creating any artefact in your assay. Please, include data without normalisation.

The possibility of quenching as well as the possibility of light to interfere with controls has been studied and is presented not normalized in Figures S14 and S15. Moreover, there is a sentence in the main text stating that there's a difference between the control ligand and our azobenzenes that may be due to these artifacts found above 3 µM.: "In contrast, compound 11b artificially decreased the signal of the

measurement at high concentrations above 3 µM in the absence of proteins (Figure S14). These results could explain the decrease in the Emax observed for compound 11b in comparison to LSN2148936.”.

It is hard to understand how some of the pEC50 values could be calculated with such accuracy when, in many cases, the plateau is not reached. Please revisit table 2.

All pharmacological curves were fitted using log(agonist) vs response (three parameters) function. This information was added in the supporting information in the methods section. This is a restrictive equation that reasonably allows to fit curves with defined the first inflection and mid points when the upper asymptotes are not represented. However, we agree with the reviewer that for compound 12c the curve is probably more difficult to fit. Therefore, we have modified Table 2 to illustrate that for this compound the pEC50 and Emax could not be determined properly.

4) The HDX difference between 11b and LSN21148936 is significant and needs extra clarification. What are the used concentrations?

The concentrations used in the HDX experiments were 100 µM for each compound and 10 µM for the VDR protein (supporting information). We selected these concentrations to ensure higher occupancy of small molecules based on their affinity. Subtle changes in ligand structure have been shown to produce unique effects on protein HDX rates. Therefore, the results obtained may be interpreted as the compound 11b (photoVDRM) binding less tightly to VDRLBD and activating the receptor less efficiently compared to the reference full agonist LSN2148936. This is somehow argued in the manuscript in the following sentence: “A closer examination shows greater protection of Helix 12 in the experiment with the compound 11b under illumination conditions, slightly lower but similar to LSN2148936 and significantly higher than the protection induced by trans 11b in the absence of light”. In other words, although 11b and LSN2148936 showed the same protection pattern, the strength of LSN2148936 protection is significantly greater. However, we did not observe significant differences in the activation of VDR in vitro and in vivo. Therefore, other possibilities may also be valid. For instance, the two ligands might promote distinct protein dynamics that both lead to active conformational landscapes. We agree with the reviewer that this is a very interesting question and further studies will be necessary to thoroughly investigate the molecular mechanisms underlying receptor activation upon binding of the different drugs presented in this work, all producing antipsoriatic effects. Nonetheless, for clarity, the previously mentioned sentence has been changed by the following:”A closer examination shows greater protection of Helix 12 in the experiment with the compound 11b under illumination conditions, lower in magnitude but following a similar trend to LSN2148936 and significantly higher than the protection induced by trans 11b in the absence of light”.

5) To avoid misleading conclusions, the results should be complete, including the trans irradiation, given the concerning 60% of the isomer. Also, if only one cycle can be performed, the general text should tone down.

The HDX-MS experiments are intended to evaluate the VDR dynamics upon interaction with the compound in its different configurations. We agree with the reviewer that it would be extremely interesting to evaluate the protein conformational landscapes on different conditions. However, as mentioned in the question, a mixture of cis and trans is promoted upon illumination with green light, which is not optimal for this type of biophysical methods that require concentrated and mostly pure

species. Indeed, this might be one of the reasons why we obtained different results between the active 11b cis compound upon UV illumination and the control LSN2148931. Further studies are planned to precisely evaluate the VDR mechanism of activation that will require technical development, synthesis of new derivatives, new methods for the analysis after different times of illumination, the purification of compound configurations and other considerations that will challenge the used of HDX-MS for photopharmacological studies. However, we believe that all this falls beyond the scope of the current study, which aims to demonstrate the feasibility of developing photoswitchable VDR agonists with in vivo activity, devoid of systemic side effects.

The word “reversible” has been removed from the manuscript to adjust conclusions with the results obtained.

6) It looks to me that there are inconsistencies in the general tendency of the activity compounds according to Table 2 and Figure 2b; for instance, I would expect a higher value for 12b (there are more). How did the authors explain these discrepancies? Is the chosen concentration the most appropriate?

As stated in the caption of the Figure 2, the concentration used for the screening was 10 µM and the activity values for the compound 12b: 51.30, 35.72 and 38.4. These values lead to a mean of 41.81 and an SD of 8.33, which is consistent with the value reported in table 2 (44 ± 5.37) that were extracted from curves in the supporting information (Figures S11-S13). These experiments were performed independently and some variation may be found, especially for compound with low activity in which the dose-response curve do not finish in the asymptote. We aimed in this study at finding compounds with low nanomolar activity. Therefore, we thought that using 10 µM concentration (2 x Log) for the screening was appropriate.

Please also include the time of irradiation, which is also an important factor for toxicity.

The time of irradiation has been added to the figure and table legends.

Please revisit the statement: “used for phototherapy and non-phototoxic visible illumination at 420 nm modulated the activity of compound 11b”

We have changed the sentence by:” Of note, we also demonstrated that UVB narrowband light (315 nm), used for phototherapy, and visible illumination at 420 nm modulated the activity of compound 11bPhotoVDRM but not LSN2148936”

7) Figure 5: B readers who are not experts with these experiments would need some extra explanation of those figures; you may consider using arrows, etc., to guide the interpretation. C significant differences (*) should be explained as in D; Are the ** at 520 nm correct? We thank the reviewer for this valuable comment. While we understand the concern regarding the interpretation of Figure 5B by non-expert readers, we would like to clarify the rationale to maintain the figure as shown.

We thank the reviewer for this valuable comment. While we understand the concern regarding the interpretation of Figure 5B by non-expert readers, we would like to clarify the rationale to maintain the figure as shown.

The H&E sections shown in panel B are intended as representative, qualitative illustrations of the inflammatory phenotype induced by IL-23 and its attenuation upon photopharmacological treatments. Importantly, the quantitative measurements of ear thickness presented in panel C were obtained in vivo using a digital caliper, across the entire ear (anterior to posterior epidermis), and not from histological preparations. For this reason, the measurements that could be derived from panel B are not directly comparable with the quantitative data presented in panel C.

To avoid any potential misinterpretation, we prefer not to include extra arrows or labels that might suggest quantitative measurements of epidermal thickness in panel B, since these images are shown only to illustrate overall histological features. In our view, adding extra indications could make readers conclude that these images were used for quantitative analysis, which was not the case

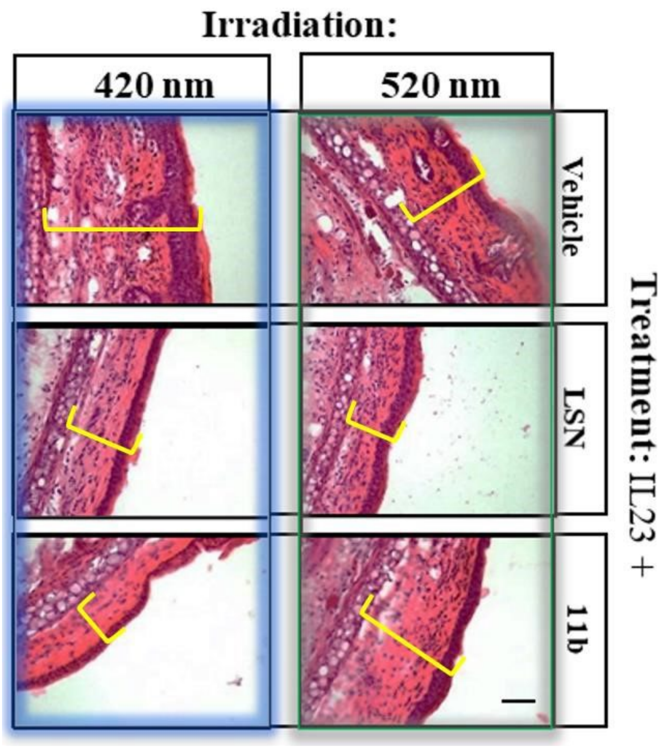

Minor comments:

a) Table 1 -> What is the composition of “buffer”? Why 12 degrees of temperature? Which light source?

The composition of the buffer and the light source has been indicated in the table. The temperature was a mistake that has been corrected in the revised version of the manuscript (“12°C” ❼ “21°C”).

b) I would tone down “we developed innovative experimental methods based on HDX-MS”. Implementation of the in situ irradiation should be incorporated in the supporting information.

We used the term “innovative” to highlight the novelty of applying HDX-MS (for the first time, to our knowledge) to study the action of photopharmacological agents binding its target. However, we agree with the reviewer that “innovative” might not be the best choice. Therefore, we replaced “innovative”

with “a modified HDX method” throughout the text. Additional details on the in situ irradiation are provided in the supporting information.

c) Why do not all compounds have the HRMS? It would be helpful to know which compounds are new and which ones have been described.

HRMS data for compounds 12c, 11b, and 10d have been included

d) Figure S5, which is the identity of the extra peaks that appear in 11a, 10c, 10d?

These compounds were obtained in small amounts and after the initial screening were not further investigated. We have not identified the structure of the extra peaks observed.

C

e) Although a reference to the quantum yield determination was included, the readers would appreciate a detailed protocol in the supporting information of this manuscript. Also include the calculation of all the compounds to verify consistency.

As suggested by the reviewer, a detailed protocol has been included in SI.

f) The calculation of lifetimes is unclear; please clarify it.

The “UV-Vis spectroscopy” section in the methods section has been completely revised. We thank the reviewer for raising this point.

g) Table 2, please include the irradiation time The time of irradiation has been added to the table 2.

h) Figure S14 “biolilinated-His6” has not been appropriately introduce in the main manuscript

We agree with the reviewer and a sentence has been added to the Supporting information for clarity:”Biotinylated-His6 peptide (PerkinElmer) was used as a quality control reagent for AlphaScreen® assays and to evaluate compound interaction with the method.”

i) Figure 2E, please specify buffer composition

We have identified buffer compositions throughout the article.

j) Figure S11 twice 10b compound

We thank the reviewer for pointing this out. We have corrected the figure.

Additional Questions:

Quality of experimental data, technical rigor: Top 10%

Significance to chemistry researchers in this and related fields: Top 10%

Broad interest to other researchers: Top 10%

Novelty: Top 10%

Is this research study suitable for media coverage or a First Reactions (a News & Views piece in the journal)?: No

Reviewer: 4

Recommendation: Publish in ACS Central Science after minor revisions noted.

Comments:
Rovira, Krishnan, Llebaria et al. report the design, synthesis, and biological validation of lightregulated vitamin D receptor (VDR) agonists for the treatment of psoriasis-like inflammation. The researchers incorporated a photoswitchable azobenzene moiety into VDR ligands, thus converting them into photohormones that allow for precise spatiotemporal control of drug activation using (365/525 nm) light cycles.

The lead compound, 11b, is only minimally active in the dark (trans form) but is converted to an active agonist (cis form) upon illumination with UV or visible blue light, wavelengths already used in dermatology. Spectroscopic studies confirmed robust photoisomerization, with thermal half-lives exceeding 6 hours in the cis state, allowing sustained activity after activation. The isomerization kinetics as a function of light intensity are informative and useful. However, the photostationary state (PSS) of 11b at 525 nm shows only 55% trans (in solution), raising the question of why the remaining 45% cis does not result in substantial receptor activation. This point should be discussed in more depth. What was the PPS at 460 nm and why was this wavelength not chosen?

The PSS of compound 11b at 460 nm is reported in the supporting information, Figure S4, panel D. We have modified the figure to easily identify the values for clarity. As the reviewer can observe, at 460 nm the proportion of cis is still 63%, therefore higher. However, we agree that the reduction of cis using wavelengths above 520 nm is not dramatic, thus raising the question pointed by the reviewer. There are several possible reasons why this may be occurring, which are not demonstrated in the current study and fall beyond its scope. One possibility is that light may affect the ligand differently when it is in solution compared to when it is bound to the receptor. Another, potentially concurrent, explanation is that the solution used for photochemical characterization may not accurately represent the biological environment encountered by the drug. Both of these hypotheses have been included in the Discussion section of the manuscript, as we agree they are worth mentioning.

Innovative hydrogen/deuterium exchange mass spectrometry (HDX-MS) combined with molecular modeling revealed that the cis form of 11b engages critical VDR residues (e.g., W286) essential for receptor activation, whereas the trans form does not. This explains the lightdependent pharmacological switch. It would be interesting to know whether this represents a case of an "efficacy switch." Perhaps the authors could address this in the discussion.

Additional text has been added to the manuscript describing the possibility of the *trans* to bind the receptor although with a different binding mode and lower affinity, which may lead to a concomitant

decrease in potency:" The considerable difference in EC_{50} and maximum observed effect between the *cis* and *trans* forms of PhotoVDRM given the relatively small difference in the docking pose and various energetic scores merits further comment. We believe that the trans form may bind to the receptor, albeit more weakly than the cis form, and that it is also less capable of activating the receptor, as reflected in the HDX data (vide infra). The combination of reduced affinity and efficacy likely contributes to the substantial rightward and downward shift of the curve."

In vivo, systemic administration of 11b in mice followed by localized light irradiation on psoriatic skin lesions significantly reduced inflammation without causing systemic hypercalcemia, a common and dangerous side effect of traditional VDR agonists. This demonstrates that light can be used to selectively activate the drug at diseased sites, offering a major safety advantage.

Prior art has been appropriately cited. However, the sentence "Photopharmacological agents have been proposed for several nuclear receptors" should be corrected to read: "Photopharmacological agents have been demonstrated for several nuclear receptors," and the references reordered to highlight the pioneering work of Trauner and Merk starting in 2019 (cf. references 12 and 13).

The proposed changes have been implemented.

Overall, this work establishes the first photopharmacological approach to light-controlled activation of the vitamin D receptor in vivo, opening avenues for safer, localized treatments of skin diseases like psoriasis. The approach may be relevant to other nuclear receptor targets and diseases where topical or spatially restricted therapy is advantageous and constitutes and important advance int the development of Photopharmacology.

We thank the reviewer for his/her positive comments.

Additional Questions:

Quality of experimental data, technical rigor: Top 10%

Significance to chemistry researchers in this and related fields: Top 10%

Broad interest to other researchers: Top 10%

Novelty: Top 10%

Is this research study suitable for media coverage or a First Reactions (a News & Views piece in the journal)?: Yes

oc-2025-00987c.R2

Name: Peer Review Information for "Light-regulated agonists spatiotemporally activating the Vitamin D receptor mitigate psoriasis-like inflammation in mice without inducing hypercalcemia"

Second Round of Reviewer Comments

Reviewer: 3

Comments to the Author

I want to thank the authors for satisfactorily addressing all my concerns. I therefore support the publication of their article

Author's Response to Peer Review Comments:

Dear Editor,

Please find the new files with the changes requested. I thank the reviewers for the appreciation of our work and the editorial office for the patience and consideration with the authors and t the help with the manuscript.

I think that the manuscript and accompanying files are ready for publication.

Yours sinecerely,

Amadeu Llebaria
